# Process and Information Needs When Searching for and Selecting Apps for Smoking Cessation: Qualitative Study Using Contextual Inquiry

**DOI:** 10.2196/32628

**Published:** 2022-04-14

**Authors:** Ylva Hendriks, Sebastiaan Peek, Maurits Kaptein, Inge Bongers

**Affiliations:** 1 Tranzo, Tilburg School of Social and Behavioral Sciences Tilburg University Tilburg Netherlands; 2 Jheronimus Academy of Data Science 's-Hertogenbosch Netherlands; 3 Research Unit Evidence Based Management of Innovation Mental Health Care Institute Eindhoven Eindhoven Netherlands

**Keywords:** mHealth and eHealth, contextual inquiry, decision-making, mobile app search and selection, apps for smoking cessation, mobile apps, mobile phone

## Abstract

**Background:**

Hundreds of apps are available to support people in their quest to quit smoking. It has been hypothesized that selecting an app from a sizable volume without any aid can be overwhelming and difficult. However, little is known about how people choose apps for smoking cessation and what exactly people want to know about an app before choosing to install it. Understanding the decision-making process may ultimately be helpful in creating tools to help people meaningfully select apps.

**Objective:**

The aim of this study is to obtain insights into the process of searching and selecting mobile apps for smoking cessation and map the range of actions and the accompanying reasons during the search, focusing on the information needs and experiences of those who aim to find an app.

**Methods:**

Contextual inquiries were conducted with 10 Dutch adults wanting to quit smoking by using an app. During the inquiries, we observed people as they chose an app. In addition, 2 weeks later, there was a short semistructured follow-up interview over the phone. Through convenience and purposive sampling, we included participants differing in gender, age, and educational level. We used thematic analysis to analyze the transcribed interviews and leveraged a combination of video and audio recordings to understand what is involved in searching and selecting apps for smoking cessation.

**Results:**

The process of finding smoking cessation apps is comprehensive: participants explored, evaluated, and searched for information; imagined using functions; compared apps; assessed the trustworthiness of apps and information; and made several decisions while navigating the internet and app stores. During the search, the participants gained knowledge of apps and developed clearer ideas about their wishes and requirements. Confidence and trust in these apps to help quitting remained quite low or even decreased. Although the process was predominantly a positive experience, the whole process took time and energy and caused negative emotions such as frustration and disappointment for some participants. In addition, without the participants realizing it, errors in information processing occurred, which affected the choices they made. All participants chose an app with the explicit intention of using it. After 2 weeks, of the 10 participants, 6 had used the app, of whom only 1 extensively.

**Conclusions:**

Finding an app in the current app stores that contains functions and features expected to help in quitting smoking takes considerable time and energy, can be a negative experience, and is prone to errors in information processing that affect decision-making. Therefore, we advise the further development of decision aids, such as advanced filters, recommender systems and curated health app portals, and make a number of concrete recommendations for the design of such systems.

## Introduction

### Background

It is well-established that the toxins in tobacco cause a range of diseases and disorders, often leading to death [1]. The World Health Organization estimates that tobacco kills up to half of its users, which adds up to >8 million people each year [2]. In addition to its major impact on mortality worldwide, tobacco use also results in a great number of morbidities [1]. Smokers die younger, age more quickly, and develop diseases of nonsmokers at a much younger age [3], decreasing the quality of life earlier in life.

Smoking cessation yields specific benefits of reducing fatal and nonfatal vascular, respiratory, and neoplastic (cancer) diseases [4]. Quitting cuts the risk of developing smoking-related diseases, such as lung cancer, by half [5] and increases life expectancy. Regardless of age, quitting smoking is always advantageous to one’s health. Smokers who successfully quit smoking before the age of 40 years avoid nearly all the increased mortality risks of continued smoking [4]. After the age of approximately 40 years, every year of smoking prevention saves an average of 3 months of healthy life [6]. Even stopping at the age of 60 years will gain a person 3 years of life expectancy [7].

Mobile apps, which are small software applications that run on mobile appliances, such as smartphones and tablets, are generally regarded as useful tools that aid people in their attempts to quit smoking for several reasons. For example, apps can provide highly individualized and intensive interventions [1,8-11]. Furthermore, apps have the ability to reach large audiences, which makes them cost-effective for both users and suppliers [1,8-11]. Moreover, apps can allow users to tailor interventions according to their personal needs [8]. Finally, apps can reach audiences who might not otherwise seek support [11], in part as apps allow for anonymity [12].

In addition, the persuasive technology literature shows that apps have certain characteristics that make them potentially suitable for supporting behavior change [12,13]. For instance, they can tirelessly continue to try to persuade users without getting annoyed or impatient. They are accessible at any time from any place and consequently able to support people in their behavior change even at night or in the privacy of their homes [1,8-10,12]. Furthermore, people sometimes view their smartphones as digital companions and effortlessly entrust personal information to them [14], thereby facilitating the aiding function of the technology. Finally, apps can present data and graphics, rich audio and video, animations, simulations, or hyperlinked content, enabling users to choose the modality of their preference [12], which could be beneficial for behavior change [15].

Apps for smoking cessation have their own individual characteristics and may vary in terms of usefulness and ease of use [16,17]; user interface design components, such as navigation, interaction, and appearance [18]; and technical quality [19]. In addition, apps for smoking cessation differ in their approach with regard to the content and its delivery. Hence, there is a fair amount of variation in the main functions of apps [20] and the degree to which apps adhere to clinical guidelines [21] and contain tailoring features [22] or behavior change techniques [16].

### Challenges in Searching and Selecting Health Apps

As iOS and Android together account for >99% of the market share in mobile operating systems [23], the Google Play Store and the Apple App Store are, by far, the largest app marketplaces in the field. The total number of apps offered by both these stores is enormous: the Google Play Store offers >3 million apps for potential users, and the Apple App Store has approximately 1.8 million available apps [24]. Although the exact number of available smoking cessation apps is unknown, a person who searches for an app in the Google Play Store, for example, receives the maximum number of search results—250 apps.

Both the Google Play Store and the Apple App Store offer a variety of information cues for each app, such as title, price, rating (stars), ranking (the order in which search results appear in a list), reviews, descriptions, categories, permissions, and the number of installations (only in the Google Play Store). In general, app developers create most of the provided information cues (ie, logo, title, and screenshots). However, the ratings are created by users. In addition, a special type of information in the search results list is the ranking of results. Ranking refers to the order in which the app store presents search results. App store algorithms determine this order, and the exact underlying factors are unclear. However, research suggests that ranking is a reflection of app success, which is, in turn, determined by factors such as the number of languages supported, package size, app release date [25], free app offers, high volume, high user review scores, and continuous quality updates [26]. Although the provided information cues may be informative, tools to guide users through the massive number of results seem to be lacking [27]. At this moment, the visitor cannot use advanced search, filtering, or sorting options in either store. The immense supply of health apps, combined with the lack of tools for refined searching, creates a situation where choosing an app based on anything other than popularity could be considered a challenge.

### Related Research

Quantitative studies on uptake, which is the act of downloading and installing smartphone apps in general, have shown that apps with a low price, high ranking, many reviews, and high ratings have the most installations [28] and that high ratings associate more strongly with downloads if customers show a degree of unanimity in their ratings [29]. This implies that these are important information cues for people when choosing apps in general. Diverse qualitative studies have confirmed these findings. In these studies, participants indicated that they relied the heaviest on ratings, reviews, screenshots, and ranking when choosing various kinds of apps, including apps for smoking cessation [30-32]. Low price or the ability to try an app free of charge are important [32,33], as are the recommendations of others [33,34], preferably given by trusted sources [32].

Specifically, for smoking cessation apps, a few studies have shed light on what people consider important, desirable, or attractive features of smoking cessation apps and which functions people believe to increase engagement. Examples include ease of use, receiving feedback, goal setting, social sharing, competition, and reminders [31,33].

Owing to a recent think-aloud study [35], we now know more about potential users’ views on factors such as capability, opportunity, and motivation influencing the uptake of health apps. In this study, Szinay et al [35] found that participants considered searches for health and well-being apps to be difficult, with some calling it a minefield. Furthermore, it was shown that during the search, people pay attention to the look and design, costs, and perceived utility of apps, among others, but primarily to the opinions of others.

These studies provide clear insights into what people generally consider important about apps and which information cues people use before downloading and installing an app. Nevertheless, to the best of our knowledge, it is still unknown how all these insights come together in the process of searching for and selecting health apps in general and apps for smoking cessation in particular. As we know little about the process, we can presently only make assumptions about what the combination of the large supply and lack of tools means for people who want to choose health apps.

### Objective

The current gap in the body of knowledge on what people do, experience, and need during the search for mobile apps for smoking cessation creates a need to better understand the process of selecting apps. Understanding the diverse information needs and decision-making processes may ultimately be helpful in creating tools to help people meaningfully select apps. What do people do and experience when searching for an app for smoking cessation? Which information is important to people when choosing an app? How do people use the available information cues in app stores (such as the Google Play Store and the Apple App Store) to obtain the desired information? This study addresses these questions by means of contextual interviews during which people choose an app for smoking cessation. This qualitative approach gives us the opportunity to elicit in situ detailed information to create a rich image based on actual behavior and people’s spoken thoughts while in action.

## Methods

### Study Design

Contextual inquiry is a technique for gathering field data by conducting field interviews with users and studying a task while it is performed in the everyday context. Directly observing the performance of the task enables the revelation of habitual and unconscious practices and is easier for participants as they do not have to articulate their practices [36,37]. A typical contextual interview, similar to a regular interview, begins with an introduction and some general questions about the participant’s situation and then moves on to observation of, and discussion about, the task under study. The researcher not only observes the participant’s actions but also pays attention to verbal clues and body language [37]. The distinctive characteristics of a contextual inquiry are the principles of apprenticeship and partnership. In a contextual inquiry, the researcher explicitly assumes the role of *apprentice* and recognizes the respondent as an expert in her or his task. Taking on this role creates a mindset that is focused on curiosity, inquiry, and learning [36]. This mindset is related to working in partnership, which facilitates true collaboration between the interviewer and the respondent to understand the task and motivation of the respondent [36]. This means that the researcher shares thoughts and confusion with the participant on the spot, thus inviting the participant to work together to understand what is happening and why.

Although contextual inquiry originates from and is typically used in contextual design projects [38-40], the method can also be applied to eHealth research [41] on, for instance, mental health [42], healthy eating [43], and persuasive technologies that facilitate healthy lifestyles [44].

### Sampling of Participants

We recruited people who wanted to quit smoking, were interested in using an app to do so, and did not currently have or use such an app. Having used a smoking cessation app in the past was not a reason for exclusion. Additional inclusion criteria were (1) owning a mobile device, (2) knowing how to download apps, and (3) being fluent in Dutch.

We recruited participants through posters and social media and by approaching people (who were smoking cigarettes) on the streets in diverse locations in the Netherlands. In addition, we recruited participants through email within our own network. Finally, we used the snowball sampling technique by asking participants at the end of the interview whether they knew someone who might also be interested in participating. To reach our goal of understanding the diverse ways in which people search for smoking cessation apps, we purposively aimed to create variations in age, educational level, and gender.

We created a simple webpage (Qualtrics) in which those interested could leave an email address. Every channel of recruitment contained a link to this webpage. We acquired 20 leads for potential participants whom we sent an information letter. We contacted every lead after a few days to check for interest in participating in the study. Of these 20 individuals, 5 (25%) no longer reacted to our messages, and 5 (25%) had decided not to participate. The reasons stated were not wanting to quit smoking or not wanting to use an app to quit after all, no interest in participating, or practical reasons. Of the 20 individuals, 10 (50%) participated in the study. During analyses of the data, we found that we had reached saturation and, therefore, decided not to recruit additional participants (see the *Strengths and Limitations* section).

### Procedure and Data Collection

Some weeks before each interview, we sent participants an information letter, informed them about the use of audio and video recordings, and scheduled an appointment for the interview.

#### Contextual Inquiry (Interview)

Interviews were conducted face to face (one on one) at a location chosen by the participant. Of the 10 participants, we interviewed 5 (50%) in their homes, 3 (30%) at the university, and 2 (20%) at their workplaces. No one else was present besides the participant and researcher, except for in 1 interview. Researcher SP conducted the interview, and researcher YH, who conducted the other 9 interviews, was present as an observer. Interviews were recorded using a digital voice recorder. During the search for an app, the screens of the participants’ appliances were shared with the researcher’s laptop (using Mobizen). The footage was recorded using the Microsoft PowerPoint function *Insert Screen Recording*. This captured both footage and sound. The researcher also took notes during the interviews to mainly facilitate revisiting certain remarks and provide a recap at the end of the interview. An interview guide was used to maintain consistency between and direction during the interviews.

Every session started with an introduction explaining the purpose of the study, talking about expectations, asking permission for recording, and answering participants’ questions. Participants subsequently provided informed consent on paper.

The introduction was followed by a semistructured interview in which we collected data on age, educational level, and smoking behavior of the participants by asking them. We also talked about prior experiences with eHealth apps, especially for smoking cessation, and about prior experiences with quitting attempts. To get a feel for the motivation of each participant to quit smoking, we used motivation rulers for smoking cessation [45]. On a scale of 0 to 10, we asked participants to indicate the extent to which they considered quitting important, how ready they felt to quit, and how confident they were about quitting. Importance, readiness, and confidence have been associated with smoking behavior change and higher scores, especially on *confidence*, indicating a greater likelihood of attempting to quit [45].

Subsequently, in the contextual interview, we collected data on the process of searching and selecting apps for smoking cessation. We instructed people to search for an app in the way they normally would if we were not present and gave no further instructions on where to start or how to go about the task. We told participants that the task would be completed as soon as one found an app that they considered good, adding that deciding there were no good apps and downloading nothing was also a valid option. We asked the participants to tell us aloud what they were doing, thinking, and feeling. We also asked questions about the task during the search, such as “what is your feeling, when you look at this app?” or “why did you go back to the search results?” (Multimedia Appendix 1 [36,45,46]).

After the participants made their final choice for an app, we jointly created a summary of the entire search process. Doing this together with the participant served as a means of checking our interpretations. By sharing our interpretations and being honest about interpersonal cues, we aimed to create a valid understanding [47]. In addition, questions we did not ask during the search to not interrupt the participant could be asked here. Before closing off, we informed the participants about the follow-up procedure and planned the date for a follow-up phone interview.

The length of the full sessions (from introduction to completion) varied from 50 minutes to 2 hours and 40 minutes, with an average of 1.5 hours (SD 34 minutes). The duration of the actual searches ranged from 17 minutes to 1 hour and 40 minutes (average 46, SD 26 minutes). It is important to note that this does not necessarily reflect *pure search time,* as, during the search, participants frequently explained their choices and voiced their ideas and thoughts. Therefore, search time is more related to the verbosity of the participant rather than to, for example, the number of apps that were reviewed.

#### Follow-up Phone Interviews

After 2 weeks from the contextual inquiry, we called the participants over the phone for a final, short semistructured interview. The researcher called the participant at the agreed-upon time. Before starting the interview, we once again asked permission to record the conversation. To do this, we used a digital voice recorder and an Olympus Telephone Pick-up Microphone. Again, we used an interview guide for the topics we wanted to address. Telephone interviews lasted between 10 and 34 (average 19, SD 9) minutes.

In the follow-up interviews, we collected data on the realization of expectations about the chosen app. Some topics we touched upon were as follows: did the participant use the app, and did the app meet the expectations of the participant, given what the participant had learned about the app during the search? In addition, we asked participants whether they had quit smoking (for topics, see Multimedia Appendix 2). Finally, we used the follow-up interview as an opportunity to come back to things participants had said or done during the contextual inquiry, which needed further clarification.

Afterward, all participants received a €15 (US $16.35) gift voucher via mail as a token of gratitude for their participation.

### Data Analysis

Audio recordings of the interviews and the phone interviews were transcribed verbatim using the f4 transcription software. We used Microsoft PowerPoint to create the so-called *process charts* in which we combined corresponding screenshots and participant quotes. These visualizations enabled us to link images on the participants’ screens to what people said at that moment (Multimedia Appendix 3). A particular strength of these visualizations was the possibility of seeing that sometimes participants misread or misinterpreted information on their screens. Quantifiable information, such as the number of apps that participants looked at and the scores on the motivation rulers for smoking cessation, was transferred to Microsoft Excel sheets. From there, we translated some data into categories. For instance, we converted information about the number of cigarettes smoked per day into three categories: light smokers (does not smoke daily), moderate smokers (<20 cigarettes/day), and heavy smokers (≥20 cigarettes/day) [48].

Thematic analysis [49] was used to analyze the transcripts (Textbox 1) and was supported by the use of qualitative data analysis software (Atlas.ti 8 [ATLAS.ti Scientific Software Development GmbH]) and process charts.

**Description of steps in data analysis.**

**Stage and description**

- *Familiarization with the data:* YH transcribed data, read and reread every transcript while listening to the recordings, and created extensive notes and memos on everything that attracted attention. We created, for instance, a memo about the observation that during the search, multiple participants wondered whether certain app features were suitable for them and whether they could see themselves using them.
- *Generating initial codes:* YH marked all possibly relevant text fragments to condense the data and clear out noise. In this step, YH also complemented memos and created new ones. The first transcript was coded independently by both SP and YH. The 2 versions were discussed in detail, and agreement was reached on what and how to code. A final single coded version was created. The remaining transcripts were coded by the first author (YH) while regularly conferring with the second author (SP).
- *Searching for themes:* YH and SP identified the initial main themes, such as starting situation of participants, navigational patterns, and use of information cues to structure the remainder of the analysis process.
- *Reviewing themes:* YH reviewed the initial themes by going through every transcript and process chart, one theme at a time, selecting text snippets and systematically creating headings and ordering fragments under the headings (open coding [50]).
- *Defining and naming themes:* In this step, to refine ideas about the themes and the narrative of the data, YH rearranged the headings, reorganized the text fragments, and reduced the number of headings (axial coding [50]).
- *Producing the report:* YH created the arrangement of the report using the themes on the final classification as headings. The final data analysis was interwoven with the writing process, meaning that we continuously alternated between writing, checking data, adjusting paragraphs, rearranging text, and selecting vivid and appropriate extracts to clarify the report of the results. Multiple iterations of the report were shared, discussed, and refined by all authors. For full, transparent reporting of this study, we used the Standards for Reporting Qualitative Research [51] (Multimedia Appendix 4, [51,52]).

### Ethical Approval

Ethical approval for the study was obtained from the institutional review board of the YH’s university—the ethics review board of the Tilburg School of Social and Behavioral Science (reference EC-2018.92).

## Results

### Overview

By analyzing the data from the interviews and contextual inquiries, we identified several facets that play a role in the search for smoking cessation apps (Textbox 2). For the sake of readability and clarity, the report in the *Results* section is structured according to the process steps, and the themes or subthemes are addressed in the description of the process step they relate to. Furthermore, the *Principal Findings* section contains a descriptive overview and summary of the themes or subthemes.

The remainder of the *Results* section is organized as follows: we start with a description of our participants, their experience with attempts to quit smoking in the past, as well as their previous experiences with smoking cessation aids and eHealth in general. Then, we describe the identified steps of the search process and search thoroughness. Next, we describe the results per process step, focusing on, among others, participants’ information needs, actions, decisions, the reasons for those decisions, and participants’ search experience. We then describe the transformation of knowledge, wishes and requirements, and confidence in smoking cessation apps throughout the search and across the process steps.

**Facets of searching for a smoking cessation app: themes and subthemes.**

**Major themes and subthemes**

- Search process
  Extensiveness and thoroughness
  Decision moments
  Differences and similarities between process steps
- Information needs
  Information cue use
  Functioning of apps
  Trustworthiness and personal relevance of the information
  Availability of information
- Information processing and decision-making
  Activities, cognitive processes, and cognitive load
  Availability of information
  Errors in information (processing)
- Transformations
  Knowledge
  Wishes and requirements
  Confidence in apps

### Sample Descriptive

The average age of the 10 participants was 41.2 (SD 8.7; range 26-59) years; 6 (60%) were women, and 4 (40%) were men. Of the 10 participants, 4 (40%) had higher education, 4 (40%) had middle education, and 2 (20%) had lower education. Every participant had started smoking as a teenager, at an average age of 16 (SD 1.8; range 13-18) years. This means that the participants had been smoking for 10 to 45 (mean 25, SD 9.3) years. Our sample of 10 participants comprised 4 (40%) heavy smokers, 5 (50%) moderate smokers, and 1 (10%) light smoker. Half of the participants mentioned stress relief as their main reason for smoking. Other reasons were having something to do at certain moments, enjoyment of the taste or the act of smoking, and regarding *being a smoker* as something positive (self-image). Most said that they probably kept smoking as it was a habit and an addiction.

### Quitting Smoking

All 10 participants had made serious attempts to quit smoking in the past: 5 (50%) participants made one attempt, 4 (40%) participants made between 2 and 6 attempts, and 1 (10%) participant reported trying 20 times. Some memories of quitting attempts in the past were positive. For example, one of the participants recalled the freedom she felt to be independent of tobacco. Another remembered the fun, game-like aspect of no one noticing that he had quit. However, most recollections of quitting attempts in the past were negative. People remembered how hard it was to quit, how ill-tempered and irritated they felt, and the guilt and shame when the quitting attempt eventually failed. Some participants specifically mentioned losing faith in their own capability to quit and being afraid of trying again:

I sooooo want to quit smoking, If I had to give it a number it would be a 10, but I am terrified to fail again.

participant 5

Reasons for wanting to quit again were health (10/10, 100%), the sake of the children (5/10, 50%), and general negative aspects of smoking such as costs, bad smell, and social disapproval. For most participants, the *health reason* was merely a rational, calculated consideration, as most of the participants did not experience any health problems at the time of the interview:

Yes, you see, if I continue smoking the chance of diseases and such is big, so then...But right now I’m fit and healthy. So in the short term that is not a motivation, but in the long run it is.

participant 7

On the motivation rulers, the participants scored an average of 7.5 (SD 1.35; range 5-10) on the importance of quitting smoking, an average of 6.8 (SD 2.08; range 4-10) on the readiness to quit, and an average of 5.7 (SD 3.55; range 0-10) on being confident that they will quit in the next attempt.

### Experience With Smoking Cessation Aids and Apps

Almost every participant had tried some form of smoking cessation aid in the past, ranging from hypnotherapy, acupuncture, and laser therapy to medication, chewing gum, and nicotine patches. Overall, 6 participants had used a smoking cessation app on previous quitting attempts. All participants had fairly low expectations of the benefits of all these aids. Everyone seemed to feel that quitting is something you need to do by yourself, that it is going to be hard no matter what, and that these aids can be *a helping hand* at most. This sentiment also applied to apps for smoking cessation. Although people found certain functions in smoking cessation apps somewhat useful or motivating, there were more comments on negative aspects, such as the inability of the app to engage them, having to pay to get access to more content, and a lack of interesting functions.

### Search and App Selection Process

#### Overview

**Figure 1:**
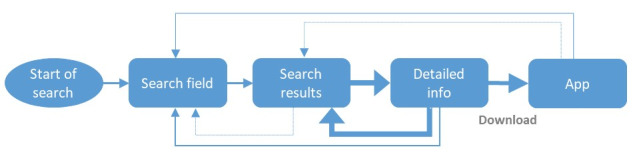
App selection process flow for smoking cessation apps. Thicker lines indicate more common occurrences.

The basic steps in the search process were the same for all participants (Figure 1): every search started with entering a search query, which led to a set of results. The next step was to choose a result to obtain detailed information. Subsequently, participants decided to either return to one of the earlier steps or move on to downloading an app. Every participant opened the downloaded apps before deciding to either choose the app or continue the search. All searches ended with participants choosing at least one app they intended to use during their quit attempts.

Although every participant’s search fitted this general process, we also saw some differences. First, we could discern 2 levels of complexity in search flows. Of the 10 participants, 6 showed a simple linear flow. They went from search queries to results, inspected between 2 and 7 different detailed app information screens, and subsequently chose 1 or 2 apps to use. The remaining 4 participants showed a more complex, elaborate flow, with more loops back to the previous process steps, using more search queries, exploring more app information screens, and downloading and discarding more than one app (Table 1).

In addition to the difference in the complexity of the process flow, participants differed from each other in search thoroughness. Some participants (2/10) only scrolled a maximum of 10 apps down in the search results list, whereas other participants (3/10) examined apps in the top 20, and half (5/10) scrolled down even further, sometimes as far as 90 apps down the list. In addition, some participants (7/10) went back to an information screen they had already seen to gain new insights, whereas others (3/10) never revisited app information screens (Table 1).

**Table 1:** Differences in search and app selection process among participants (N=10)^a^.

Number	Process flow	Process flow					Search thoroughness	
		Number of different search queries	App information screens (n=85), n (%)	Apps downloaded and opened (n=19), n (%)	Apps chosen for use (n=12), n (%)	Rank of app scrolled to		Revisiting information screens
1	Complex	3	10 (12)	3 (16)	1 (8)	9		Yes
2	Linear	1	2 (2)	1 (5)	1 (8)	12		No
3	Linear	1	6 (7)	1 (5)	1 (8)	12		Yes
4	Linear	1	5 (6)	1 (5)	1 (8)	60		No
5	Complex	4	25 (29)	1 (5)	1 (8)	92		Yes
6	Complex	4	9 (11)	2 (11)	2 (17)	21		Yes
7	Linear	1	7 (8)	2 (11)	2 (17)	28		No
8	Complex	3	11 (13)	6 (32)	1 (8)	16		Yes
9	Linear	1	5 (6)	1 (5)	1 (8)	96		Yes
10	Linear	2	5 (6)	1 (5)	1 (8)	10		Yes

^a^App store could be either the Google Play Store (2, 4, 6, 9, and 10) or the Apple App Store (1, 3, 5, 7, and 8).

#### Start of Search

The start of the search differed among participants in 2 ways. First, the participants used different devices for the search. Of the 10 participants, 1 started the search on a laptop (switching to a smartphone later on), 2 used a tablet, and the remaining participants searched for an app on a smartphone. Participants starting the search on a laptop or tablet indicated that they thought a bigger screen was somewhat easier for searching and reading.

The second difference was related to the place where the participants started their search. Most participants (8/10) went straight to the app store, whereas some (2/10) participants started their journey in a web browser, using a search engine to visit ≥1 website to gain information about apps for smoking cessation before going to an app store:

Because I don’t know what I’m looking for, it’s nice to spend some time online reading. That gives me some “language”, some inkling of what to think of, and after that, I go on to the list of apps.

participant 6

#### Search Field

Every participant began by using a search function (either an app store search field or a search engine such as Google). Of the 10 participants, 9 started the search with a Dutch query, and 1 initially used English terms (Multimedia Appendix 5). Seven participants clicked on a query offered by the autosuggestion. Four participants returned to the search function later in the process to use another query (using different terms or switching to English or Dutch) in an attempt to filter the search results (eg, on free or skin) or to search directly for a specific app (by name).

#### Search Results

After entering a search query, users received a number of results. All participants who searched the Google Play Store and those who searched the Apple App Store with an English search term received only smoking cessation apps in the results. However, Apple users who used a Dutch query received a mix of smoking cessation apps and other unrelated apps, such as Stop Motion apps. One of the participants scrolled down to the 32nd app in the search results list; of the 32 apps, 17 (53%) were not smoking cessation apps.

The information per app in the list of search results in the Google Play Store was limited to a logo, title, rating (number of stars), price (if applicable), and developer name. The Apple App Store additionally provided screenshots but omitted the developer’s name. Some participants (4/10) indicated that they thought the information was scarce. For example, they stated that all the ratings were basically the same, and thus unhelpful, and that the other information was hardly useful for making a proper choice. In addition, some remarked that a small text about the functionality of the apps and a means of filtering the results on price were lacking.

The decision people made in this process step was to click or skip an app in the search results list. Most participants (8/10) mentioned that they based their decision on ≥1 available information cue. People most commonly used screenshots (only in the Apple App Store), ratings, price, and the name of apps; however, click-or-skip decisions were also based on logos and developer names. Some participants (2/10) systematically opened app detail pages by first clicking the first app, then the second app, and so on. Hence, these participants did not use any information cues for their click-or-skip decisions.

For the participants who explicitly mentioned using the information cues, we discerned 3 main reasons for clicking or skipping apps. The first was a positive or negative evaluation of some aspects of the app that was reflected directly in the information cues. For example, this was an evaluation of the design of the app based on screenshots, the popularity of the app based on rating, or the trustworthiness of the developer based on developer name (ie, Trimbos Institute). Sometimes, screenshots and app names provided some information about the functionality of an app, on which people based a click-or-skip decision. For example, the term *audiobook* in app names could attract or put off participants. Overall, people clicked an app when the evaluation was positive (design attractive, developer a trustworthy party, rating high, and desirable functionality) and skipped apps when the evaluation was negative (app costs money, design unattractive, rating low compared with other apps, and functionality undesired).

The second reason to click on apps was to check something. For example, several participants clicked on apps to check whether the app was, in fact, a smoking cessation app. In addition, half of the participants indicated some confusion over whether they had already opened detailed information for certain apps at some point during the search. To check this, they clicked or reclicked an app in the search results. Furthermore, one of the participants clicked apps out of curiosity and a wish to check what an app was about (triggered by such things as *hypnosis* in the app name, a combination of a trustworthy source and low rating, or a funny name or concept). Finally, one of the participants clicked some apps because of a personal conviction that “one has to check something out to judge it” (participant 10), although the information in the search results did not trigger a particularly positive evaluation of the app.

The third reason for clicking or skipping an app was based on the participant’s imagined idea about the working of the app. On the basis of the available information, some participants immediately formed a picture of how the app would work and subsequently clicked on the apps they thought were right for them and skipped the apps they evaluated negatively. In some cases, this interpretation of information led to a decision to skip based on nothing more than a logo, app, or developer name:

The little man here, this one, the green one, kicking his cigarette...I would click that one sooner than this woman with “Quit Buddy”. [...] She’s going to ask you nicely all the time, I think, or in any case, [she is going to tell you] “well done” all the time. All the time these motivational things. I couldn’t take that very well, I think. But that’s my first insight. Yeah, I don’t know, that [other] one kicks your ass, I guess.

participant 4

Here: “David Crane, PhD”. Somehow that has a weird, nasty...[...] somehow I don’t really trust that. Like: here comes [...] mister PhD who will tell us..., He is being paid to promote this. [...] In my head that just turns into something negative. Yeah, that’s a personal thing, that I think “what an exaggerated fuss”. But, I was like...I have three more to choose from, so I’m just not going to look at this one.

participant 10

#### Detailed Information (App Information Screens)

Clicking an app in the list of search results led to a screen with detailed information about a specific app. The information on these screens was far more elaborate than the information in the search results list, containing, among others, screenshots, a description, reviews and additional information about the developer, version, and permissions.

Participants used between 3 and 12 different information cues to gather information about apps. The main sources of information were descriptions, screenshots, reviews, and ratings; however, some participants also considered, for example, the ratio between the number of reviews and ratings, date of the last update, and developer response to reviews. A few participants paid attention to the number of installations (only Google Play Store), and none looked at information about permissions. Some participants showed a clear preference for textual information, others for visual information, and most used both. Most of the time, participants browsed the information; however, sometimes, they went in active search of particular information about, for example, costs or user-friendliness.

While going through the detailed app information screens, participants performed several actions. The most important actions were as follows: (1) they explored information about the functioning of smoking cessation apps, (2) some participants tried to assess the trustworthiness and personal relevance of the information itself, (3) participants formed opinions about diverse functions and characteristics, (4) some participants also imagined what using certain functions would be like for them in practice, and (5) everyone eventually decided to either download an app or leave the detailed app information screen and continue the search. We describe each action in more detail in the following sections.

The primary action on the detailed app information screens was *exploring the information about the functioning of smoking cessation apps* to create a mental image of smoking cessation apps in general and of specific apps in particular. First and foremost, all participants paid attention to what these apps do and how they work by focusing on information about the specific functions of apps, such as time, cigarette, and money counters; challenges; badges; and chat functions. Furthermore, most participants tried to determine whether apps functioned well technically and whether other users were positive or negative about the apps in general and about certain functions in particular. Another important information need was *the price of a free app*. Many participants wanted to know what *hidden costs* were associated with the free apps. These participants were looking for information about the difference between free and paid versions of the same app; whether one had to start paying over time; and whether paying for an upgrade would get them extra functionality, quality, or just the elimination of annoying advertisements and pop-ups offering upgrades. Finally, several participants looked for information about the quality and professionalism of apps to estimate their trustworthiness. Cues for a trustworthy app could be the name of the developer (known institutions and familiar names generally inspired trust), beautiful design of the app, mention of a scientific foundation, or reactions by the developer to reviews:

There’s always a reaction [from developer to reviews] too, right. They always give a...That’s definitely positive. Professional. Like, at least he’s involved in his own app and taking it seriously.

participant 1

For some participants, a second action while examining the detailed app information screens was trying to *assess the reliability of information itself*. Half of the participants were engaged in estimating reliability to some degree, which was particularly complicated for reviews:

But then again, I don’t really know how that works [...] actually, with apps and with reviews. [...] Yes, [I don’t find it credible] that there are so many. [...] I don’t really believe that all of that is true, what it says there. Of course, it’s also just that it could be someone from Vietnam, who gets paid to write reviews there. I think so. Or, I don’t know from which country...

participant 4

Furthermore, at some point, half of the participants tried to *estimate whether the information was relevant to them* in their search for an app:

“I made a back-up and put it back.” [...] Oh, that’s just someone who doesn’t know how to [...] transfer that to their new phone...That is not applicable to me. She was actually more critical of her inability to install a new phone than of the app itself. [...] That’s not a review of the app. Yeah, so then I think, yes, I can sit and read all that nonsense, but it comes down to how it ultimately feels and pleases me in terms of use.

participant 10

Third, while examining the detailed app information screens, all participants *formulated opinions about functions and characteristics*. These opinions varied from person to person. There was consensus on some functions: the counters and badges were positively regarded by many participants, and the inability to choose one’s own quitting date, even if it were in the future, was regarded negatively by most participants. Opinions varied greatly regarding some functions or characteristics:

I’m more one for shock therapy, like: “Stop, stop now! You’re getting cancer!”, like that. [...] seeing a rotten toe, or something, you know, getting eye cancer from it, that sort of stuff. That impresses me, you know [...] So you have to motivate me, or yeah, punish me, motivate me with my health.

participant 9

Yeah, you know what the crazy thing is? Yeah, that sounds terrible, I don’t know if you’ve heard it before, but you know the heart attacks and the lungs, yeah, that doesn’t motivate me. Is that bad? [...] This is a very threatening one, with the number of deaths since you stopped smoking and...But that’s not my motivation. [...] they’ve gone out of their way here to make you very afraid in any case, but that doesn’t work for me.

participant 5

Paying for apps was a topic on which all but one of the participants gave their opinion. Of the 10 participants, 4 (40%) were prepared to pay for an app but only if it bought them the extra functionality they desired or if it was a guarantee for a high-quality app; 3 (30%) were not strictly unwilling to pay for an app but thought the free ones would do just fine; and for 2 (20%) participants, paying for smoking cessation apps was an absolute *no go*.

Fourth, next to exploring functions, assessing the reliability and relevance of information, and forming opinions, a number of participants *imagined what using certain functions would be like in practice*. They tried to imagine how and in which situation they would use a specific function:

“Track your cravings and learn how they can get better over time”. So apparently, I can register when I’m craving a cigarette. That’s kind of interesting because then I can measure it for myself...I know where my weaknesses lie, but [...] I find it interesting because I do think it is fun to do self-examination [...] I do think it’s a nice feature, but...I dont think I will make very active use of it, if it’s, like, half past one in the morning and I think “I feel like having a cigarette”, I don’t think I will grab my phone and think, “Half past one in the morning, I’m sitting here on a terrace [...], a glass of wine in my hands and I feel like having a cigarette now. Ohh...” I don’t think I’m going to do that.

participant 8

Finally, at some point in the search, every participant had to *decide to either leave the detailed app information screen or download the app*. This choice was the result of the four abovementioned actions: exploring and imagining resulted in a mental image of smoking cessation apps; opinions about the functions, characteristics, and trustworthiness of the apps; and an assessment of the reliability and personal relevance of information, resulting, in turn, in decisions to either download the app or leave the screen.

In total, the 10 participants opened and left 85 information screens (range 2-25). In some cases, the reason for leaving a detailed app information screen would be practical, such as wanting to see more apps, comparing some apps with others, or an app turning out not to be a smoking cessation app. However, most of the time, people left these screens as the assessment of (some aspects of) the app came out negative. The most common reason for appraising an app negatively was finding a particular function or feature in the app unappealing, unhelpful, or not in accordance with (developing) wishes or requirements. In addition, doubts about the reliability of the app or a certain approach played a role in the negative assessment. Furthermore, bad reviews from others or a small number of reviews and ratings often caused participants to assess an app negatively and leave the detailed app information screen. For half of the participants, the presentation of information in itself played a role at some point. For the participants, language and spelling errors, poor (automated) translations, and a perceived cluttered structure of text or screenshots were the reasons for leaving a detailed app information screen and continuing the search.

At some point, every participant chose to download an app. The first app was downloaded after participants had viewed, on average, 5 detailed app information screens (range 1-9). Overall, 4 participants downloaded ≥1 app (Table 1). Overall, 2 of them (participants 1 and 8) downloaded multiple apps (3 and 6 apps, respectively) to find a specific desired requirement. One of the participants (participant 7) downloaded 2 apps wanting to view them both *live* and then decide which one to keep. One of the participants (participant 6) downloaded an app that he wanted to listen to *just for fun in preparation* in addition to the one he planned to use during the quit attempt. Most participants indicated that they were downloading apps as part of the search process to explore the apps to see what they were like in practice.

#### The App

All participants opened the apps that they downloaded. Two participants (participants 3 and 6) decided, immediately after opening them, not to explore the chosen apps on the spot. One of them wanted to enter data privately after the interview, and the other did not want to start the trial period at that particular moment. The remaining 8 of participants explored their downloads.

All participants started exploring by clicking on the menu options and buttons to see (and discover) what the app did, how it worked, and what the possibilities were. Exploring the apps resembled the exploration of the information on the detailed app information screens, in the sense that participants stated what they liked or not and what they thought would be helpful. Similarly, the participants imagined whether and how they could potentially use certain functions in practice. In addition to exploring, a number of participants actively sought the functions or features they desired.

Exploring the first download led to the decision to either keep or discard the app. Of the 8 participants, 5 remained (or became more) enthusiastic about their first download after exploration and chose to keep the apps (participants 2, 4, 7, 9, and 10). One participant (participant 5) ran into an *Upgrade to Premium* pop-up, which discouraged proper exploration of the app and made her continue the search without discarding the app. This participant went back to the app store and looked at 17 more detailed app information screens before returning to the initial download and exploring it more thoroughly. After the second exploration, the participant concluded that the app was truly the best one she had encountered and that it actually met her wish or requirement. Of the 8 participants, 2 (participants 1 and 8) discovered something they really disliked about their first app during the exploration and decided to discard the app and continue their search:

I don’t even get to choose tomorrow! Or do I have to...? It says here: “Last year.” So I can go into the past, but I MUST stay in the now. [...] I don’t have a choice. I can’t say I want to stop next week because I’m starting medication now for example. [...] They just assume...I want to download the app and they just assume “now you don’t smoke anymore”. Yes, now I’m already inclined to...I’m curious how that works in the other apps. Whether they also just say “bam”...[...] Well, what irritates me most, or bothers me, is that I am not allowed to choose when I want to stop. [...] I’m just going to find another one.

participant 1

From that point on, these 2 participants (participants 1 and 8) changed their way of searching. They had chosen their first downloads as, based on the information they had viewed on the detailed app information screens, they found certain features fun and attractive, could imagine them as helpful, and found the design appealing. After they came across the aspects in their first downloads that were so objectionable (the setting of the quit date in the future and the costs of the app), their search turned into a hunt, really only paying attention to that one requirement. Both decided to keep a downloaded app as soon as they found one that met the requirements.

Choosing to keep an app (and thus stopping the search) was related, in the first place, to satisfaction with certain characteristics and functions but also to a sense of saturation. Half of the participants indicated that they felt they had *explored enough apps*. For some Apple users, this meant that they felt they had viewed the full range of products as the app store returned a limited number of relevant results for a Dutch search query. For a few participants, saturation occurred as their search had taken quite some time, and they had viewed a lot of information. One of the participants was saturated after reviewing a self-pronounced delimited set of the first 10 apps in the search results list.

For a number of participants, in addition to satisfaction with the functions and features and saturation, feeling certain emotions played a role in choosing and discarding an app. Several participants were simply excited enough about the app they had downloaded, opened, and explored to stop searching. One of the participants was surprised to have eventually found exactly what she was looking for. Two participants were tired of searching; 2 others were extremely frustrated during the search and were so relieved when they had finally found something that met their needs that they immediately ended the search:

Whaa! Help. What frustrations...My god. [...] Uhm so no, now I’m like...[...] But what I’ll try one more time is to enter “quit smoking” now instead of...See if I get completely different results now. [...] We’ve already seen this one, we’ve also seen that one, we’ve also seen that one...Not this one. [...] [I]m not seeing anything annoying yet, so. I have my health things, I have my milestones. And apparently this is free so then...great. Okay, well, we have an app. And I don’t want to think about it any further now [laughs].

participant 8

#### End of Search

Eventually, every participant ended the search with at least one app and the intention to use it during the next cessation attempt. More than half of the participants felt that they could not still properly judge the app and its usefulness before using it for some time. Several participants indicated that if through use, they would discover that they did not like the chosen app after all, they would have no problem getting rid of the app, switching to another app, or starting to look for other support tools (such as medication or e-cigarettes) for the cessation attempt. This low threshold for discarding the app seemed to be related to the apps being free.

Looking over the process as a whole, across the separate process steps, we observed additional factors that played a role in the choices people made, such as ranking and rating, feelings, and errors in information processing. We describe each factor in more detail in the following sections.

First, the roles of both *ranking* and *rating* in making choices were somewhat ambiguous. Apart from one participant, none literally named *ranking* as important information in their search. Moreover, half of the participants scrolled down further than rank 20 in the search results, and approximately a quarter of the viewed detailed app information screens were those of apps with a ranking >20 (maximum 94). Thus, during the search process, ranking did not seem to play a role for our participants. However, the apps that the participants ultimately chose to use were all in the top 10 in terms of ranking; therefore, ranking did seem to be of influence on the outcome. Similarly, for *rating*, although many participants also viewed information screens of apps with very low ratings (range 2.3-5) and of apps with no rating (because of too few reviews), for some participants, we observed that rating played an important role in the choice of clicking or skipping apps in the search results overview. Moreover, the average rating of the chosen apps was 4.5 (range 3.9-4.8) stars, whereas the average rating of all viewed apps (that had ratings) was 4.3. Once participants arrived on the detailed app information screens, rating seemed less important for some as their focus was drawn to functionalities, design, or other features that excited them or that they considered important.

Second, in addition to rational arguments for choosing to click or skip, leave a detailed information screen, or download or discard an app, almost every participant indicated somewhere in the process that they made a certain decision as something did or did not feel right. For example, one of the participants did not have a good feeling about a particular app while reading the information in the search results and on the detailed information screen. He associated the developer’s name with a treatment for alcohol addiction, the app came across as American (“not my favorite...um, people, in terms of attitude and behavior and such” [participant 10]), and he found the use of the word PhD in the developer’s name annoying, as well as the mix of Dutch and English in the description. Strictly speaking, none of these things had anything to do with the content or quality of the app; however, nonetheless, they discouraged him from choosing the app.

Finally, we observed the influence of errors in information and information processing on the decisions participants made throughout the process. For all participants, somewhere in the search process, something went *wrong*. It could be that people missed something in the information, did not read it properly, misinterpreted it, or misremembered it. In addition, the information itself was sometimes unclear, incomplete, or hard to find. As a result, people occasionally drew wrong conclusions and made wrong assumptions. A number of times, we observed that choices (click, skip, download, or discard) were based on a judgment that was formed on information that was misread, misinterpreted, misunderstood, or misremembered or as information could not be found.

In many cases, these kinds of decisions did not necessarily have any kind of impact. For example, one of the participants (participant 3) mixed up all kinds of information she had seen and read. After making the choice, she mentioned that she thought usability was important, as well as the large number of reviews (as to her, that was an indication of many downloads and, thus, popularity, which she considered important). She remembered reading in the reviews of the app she chose that the app was user-friendly. However, the recorded images showed that none of the reviews said anything about user-friendliness. She also remembered that one of the apps she had not chosen had very few reviews. However, the images showed that, of the 6 apps this participant reviewed, the one she referred to was one of the apps that had the most reviews, and the app she had chosen turned out to be one of the apps with the fewest reviews. Thus, it seemed that this participant had misremembered that *negative* features belonged to apps she did not choose, and features she found positive belonged to her chosen app.

In some cases, errors in information (processing) led to a profoundly negative experience or an inferior choice of app. For instance, one of the participants (participant 8) had a very frustrating search caused by not reading carefully and as certain information was hard to find. She mistakenly wrote off several apps that were fully compliant with her requirement for a free app with certain basic functionality. Another participant (participant 10) who did not have a good feeling about a particular app wrongfully assumed the things that made him feel bad about the app (the app was not American but British, eg, and the mix of Dutch and English in the description was caused by an app store functionality and not chosen by the developer). If the participant had not made these errors in information processing and had not written off the app for these reasons, he would have had a higher quality app in this one than the one he ultimately chose.

Thus, over the whole process of ranking and rating, feelings and errors in information processing had some influence on the choices people made. On the other hand, we observed that privacy-related information was not important for any of the participants anywhere in the process. None of the participants viewed the information about permissions on the detailed app information screens. After opening the downloaded apps, almost all participants instantly agreed with their privacy policies, terms, and conditions. A total of 2 participants first quickly scrolled through the text before giving consent but also immediately indicated the futility of that action:

Yes, actually I always just “agree” [laughs]. I don’t quite feel like reading all the way through, that ehh. [...] I just think, it’ll be fine.

participant 4

I did read for a while, but then I couldn’t choose anything there. I mean, that was it, so yes, I couldn’t do anything else there except click on it because otherwise I couldn’t continue.

participant 9

#### After 2 Weeks

**Figure 2:**
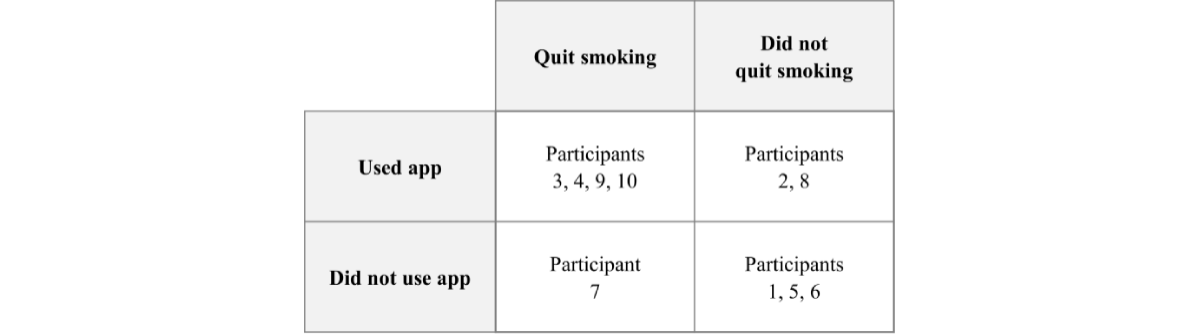
App use and quitting success after 2 weeks.

After 2 weeks from choosing an app, of the 10 participants, 6 (60%) had used the app to some extent, of whom 4 (67%) had also quit smoking (Figure 2). Alternatively, one of the participants had quit smoking without using the app. Finally, 3 participants had not used the app and had not quit. Of these 3 participants, 2 had already indicated at the end of the interview that, because of personal circumstances, they were not confident that they would actually start their quit attempt right after the interview (both scored 1 on the confidence ruler), and 1 had not managed to start the quitting attempt, although she was rather enthusiastic about the app and had been moderately motivated to quit during the interview (score of 5 on the confidence ruler).

The 6 participants who had actually used their app had enjoyed occasionally using some functions in the app (the distraction game and the motivation cards) or viewing certain information (the counters and health information). Of the 6 participants, 5 had only used a small number of functions and to a limited extent, and 3 of them indicated that the app could not do much other than count days, cigarettes, and money; however, these participants also immediately admitted that they had not actually explored the app thoroughly. They realized that there might be more functionality available in the apps. For these participants, the app had not played an important role in quitting. However, of the 6 participants, 1 had used the app more extensively and indicated that the app had supported him in his quitting attempt.

In retrospect, what the participants remembered most about finding an app for smoking cessation was that many apps, more or less, offered the same functions and looked similar, making it hard to distinguish among them. We also saw this at times during the search when participants tried to remember the features of a particular app. At such times, it appeared that people mixed up (information about) apps and, in some cases, did not remember whether the app had already been viewed or even downloaded. Combined with hard to find, limited, or absent information, sometimes, the only way to find out about something was to download the app. Consequently, a number of participants indicated that they thought it actually takes (too) much time, effort, and energy (in some cases because of negative emotions) to really look for an app properly. For some, the frustration of the search was still fresh in their minds:

Going back to look for another app? No, no, I found that process só tedious, already after 5 minutes. I’m really not going to do that again, no. Haha, no, I found searching for those apps, oh my god...Those frustrations all the time, no, oh no, no.

participant 8

The participants who had used the app intended to leave it on their phones for now, mainly for the counters. Participants who had not yet used their apps intended to save them for their next quit attempt. Thus, no one expressed any intention of going back to the app store or looking for another app.

### Transformations

From the start of the search until 2 weeks after searching and selecting and, in some cases, using the app, we observed transformations in 3 distinct areas. Over time, participants gained *knowledge* of the workings of smoking cessation apps and simultaneously developed clearer ideas about their personal *wishes and requirements* for an app. However, *confidence and trust* in the ability of these apps to really help while quitting remained quite low or even decreased. We describe the changes in each area in more detail in the following sections.

Before starting the search, every participant could think of at least one or two basic functions (eg, counters and notifications), remembering these from earlier experiences with smoking cessation apps or from stories they had heard from other people. However, none of the participants, including those who used an app in the past, had any knowledge of what currently available smoking cessation apps were able to do and offer. During the search, every participant recognized the basic functions and also discovered new functions and features in smoking cessation apps they had not known or realized existed beforehand. After the search, all participants felt they had a more complete picture of the range of smoking cessation apps, what they can do, and what the landscape looks like.

For all participants, learning about functions and features went hand in hand with forming ideas about what they wanted and did not want from an app. While gaining knowledge, participants developed ideas about what they would like, enjoy, or (on the contrary) find irritating and annoying about an app (wishes), as well as what they thought would or would not help them and, thus, be important in an app (requirements).

The development of wishes and requirements could even continue after choosing an app. The functions and features participants had liked during the 2 weeks of using the apps were often things they had already noticed during the search. However, in some cases, participants were surprised by the fun aspects that they had not seen information about while searching. Notably, a few participants were surprised to find certain functions in the app that motivated them and changed their minds about those functions. For example, one of the participants gave his opinion about a specific feature while exploring the app during a contextual inquiry:

Well, the way that works, I just find that weird. Because [...] if you accidentally shake your phone, another one of those things will appear. I don’t need that.

participant 10

After 2 weeks, the same participant said the following:

If you have your phone in your hand and you shake it too hard, then it automatically gives those quotes and stuff on the screen, so to speak. Sometimes when you’re not even [...] engaged with it, and you pick up your phone, then suddenly there’s this thing on the screen, so to speak [...] I think that’s a good thing. You don’t really get the chance to forget about it, or to let your attention wane, so to speak. So that, yes, for me that works.

participant 10

**Figure 3:**
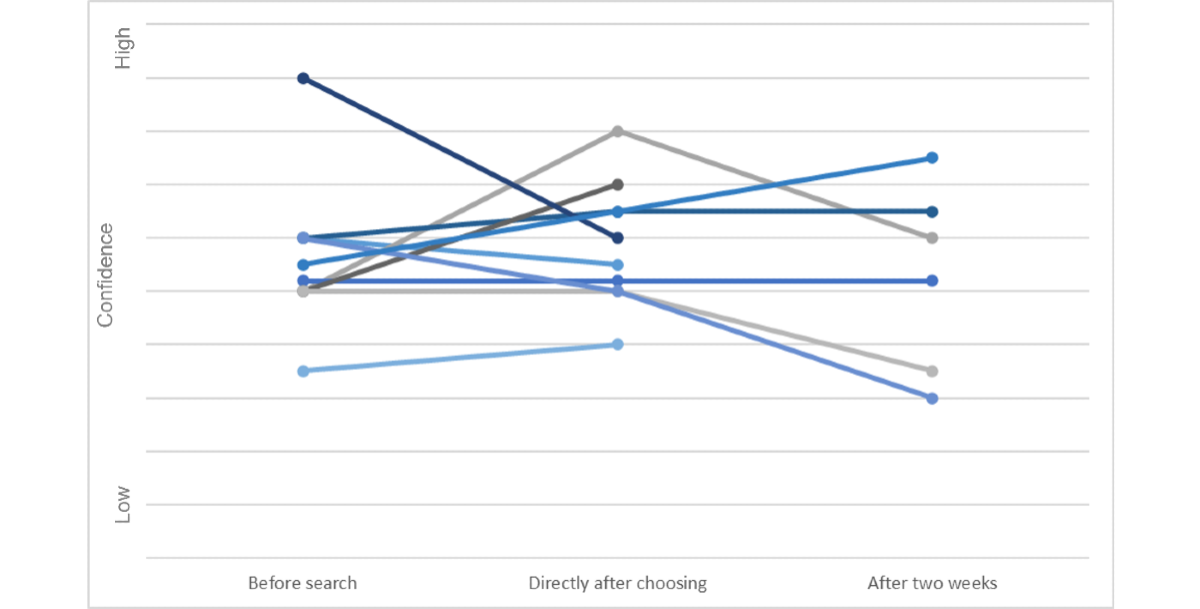
Confidence in the helpfulness of smoking cessation apps per participant at 3 points in the search process.

The transformation of *confidence* in the helpfulness of smoking cessation apps was slightly more *fuzzy*, with no clear patterns or groups (for an impression of the fuzziness in the changes in confidence, see Figure 3). Generally, confidence was not very high for any of the participants beforehand. For some participants, this was because of a mediocre experience with these types of apps in the past. For almost all participants, low expectations about the ability of apps to help with quitting seemed linked to low confidence in the ability of cessation aids to help during a quitting attempt in general:

Well, it’s certainly not going to be the ultimate remedy. I’m too stubborn for that anyway and I know that I, I have to do it myself. And aside from someone coming and sitting next to me all day and knocking every cigarette out of my hands...There’s no way the app is going to do that.

participant 10

Immediately after choosing an app, participants were asked to estimate their confidence that the chosen app would actually help them quit smoking. For some, confidence had increased slightly compared with the confidence participants indicated having in smoking cessation apps in general before the search; for a few, it was similar; and for one, it had dropped significantly. Although this participant felt that he had chosen *the best* app available, he had become disappointed in the *landscape* of these types of apps through the extensive search:

[...] especially telling that during my search, no really serious things come up. I think that’s a very simple fact that says a lot. For example, that there doesn’t seem to be an app that costs 100 euros a year. That makes the domain serious, that world, that makes that there is a landscape. That there are things that cost 100 euros, things that are free or a few euros. Then there would be something of a landscape, and now there is not. Actually, we have seen 2½ things. 2½ ways of...That is, a very simple counter and an app that has something of interaction in terms of cravings. [...] Yeah, the disappointment that I feel now at the end, like: “yeah, it’s just not there, or something, that [serious] app.”

participant 6

For a couple of participants, the disappointment in the chosen app manifested only after 2 weeks. During the extensive search, they felt that they had looked at enough apps (sometimes *at everything there is*) and had chosen *the best app* from the range on offer, only to discover during use that even the *best* was not very good:

I did hope [that this app could hold my interest]. I think, you know, I was going to do a really good search, and that’s what I did with you at the time, but no, [...] no. [...] There’s nothing innovative in it. | [...] Maybe I thought, “well, this is it then” because I chose very consciously [...] and didn’t simply take the first one I could find. Then I think, well, this is going [...] to be the Columbus’ egg. But it turned out not to be.

participant 9

A few participants, who had not had a high opinion of quit-smoking apps 2 weeks earlier, found their lack of confidence confirmed during use:

I don’t find the app reliable, because after every day it says: “you will live 60 minutes longer”. Then I think: “yeah, bullshit probably”. Or just things where you think: yeah, I don’t know...this is probably just not true. Anyway, it’s kind of funny to see [...] [but] I don’t take it very seriously.

participant 8

For most participants, even for those who were enthusiastic about certain aspects of the app, confidence in the app’s ability to help them quit smoking did not increase after 2 weeks compared with the confidence they had immediately after searching. Again, most participants indicated that quitting smoking is simply hard and a matter of perseverance and discipline and that no app in the world can do anything to make it easier:

I do adjust the grade down a bit, to a six [instead of an eight] in the sense that it really helps to stop smoking. [...] you can’t stop smoking just by using an app. I mean, there’s more to it. But there’s nothing the app can do about that.

participant 2

## Discussion

### Principal Findings

This study set out to explore the process of searching and selecting apps for smoking cessation and map the range of actions and the reasons for those actions during the search, focusing on both the information needs and experiences of those who aim to find an app. The empirical findings in this study have expanded our knowledge of the process, information needs, information processing and decision-making, and transformations that occur when searching and selecting apps for smoking cessation.

With regard to the *process*, we found that participants thoroughly searched for an app that they expected to contribute to smoking cessation. All participants were actively involved in exploring, evaluating, imagining, comparing, searching, assessing, choosing, and navigating. The comprehensiveness of the search was reflected in several aspects. Many participants continued to look at app information screens and download apps to find something they were somewhat confident in, even if they were fed up or frustrated. The most extensive searches involved using multiple search terms and going back to previously viewed app information screens to discover more and compare apps. Participants viewed many detailed app information screens and scrolled far down the list of search results. No one used a *Take the First* heuristic [30]; one of the participants chose an app after viewing 2 detailed app information screens; however, otherwise, everyone viewed ≥5 screens. Many participants read texts thoroughly. Only 1 participant hardly read at all and chose to download apps based on heuristic cues such as ratings and pictures. Most participants also explored the downloaded app as part of the search process. Searches took quite some energy: although there was laughter and participants were generally happy to be looking for an app, for some, the whole process caused negative emotions such as frustration, irritation, and disappointment. Furthermore, several participants indicated fatigue at the end of the search. Overall, it appeared that although confidence in the helpfulness of smoking cessation apps was low, everyone made a real effort to find the *best possible* app.

The search process of our participants was far more extensive than we had expected based on one of the few studies on choosing apps [30]. In that study, only 16% of the participants used a strategy of viewing >1 detailed app information screen before making a choice when choosing (among others) a running app. This former study was conducted in a laboratory setting, used special research devices, and was conducted with participants who did not necessarily have use for a running app. Our participants may have been more invested as they were looking for an app they actually intended to use on their own devices and in their own personal context. It was also notable that all 10 of our respondents chose and downloaded an app with the intent of using it, whereas uptake was found to be far lower in other studies [35]. This result may also be related to the level of investment. Alternatively, it may have been the formulation of the task (to *search for an app like you would do if I were not present*). Although we took care to tell the participants that deciding not to download an app was also an option, emphasizing that choosing an app was certainly not required to end the task, the task may have been leading. Another result we did not expect was the extent to which participants scrolled down the list of search results. It is well-known from research on internet searches that people never scroll down further than the third page [53,54]. However, what is consistent with research in this field is that the first and second results were viewed most often.

Second, with regard to *information needs*, our findings show that participants mainly paid attention to and went in search of information about the functioning of smoking cessation apps. In doing so, they mostly paid attention to what these apps do and how they work, whether apps functioned well technically, whether other users were positive or negative about the apps and their functions, *the price of a free app*, and quality and professionalism of apps. In addition, some participants tried to assess the trustworthiness and personal relevance of information itself.

Information about the functionalities included in an app (eg, counters, community, Facebook, and coaching) and what features the app has (ie, design and price) was easily found by most participants and could be obtained from descriptions, screenshots, and reviews and by exploring downloaded apps. Information about the content and technical quality of apps could not be gleaned directly from descriptions and screenshots and was, therefore, more difficult to find. Some participants dug deep to assess whether app developers had proper expertise, whether the intervention was good and reliable, and whether it was based on a scientific foundation but often could not find any information about it despite the extensive search. Information about the *true costs* of free apps was equally hard to find.

Nearly everything we have observed in terms of information needs is consistent with previous research. As in previous research, our participants paid attention to features such as monitoring, feedback, goal setting, rewards, reminders and prompts, progress sharing on social media, coping games, health and statistical information, communication style, and ease of use [31-33]. Recently, a study by Szinay et al [35] showed that people also primarily pay attention to these potentially engaging characteristics when searching for health apps. In addition, similar to participants in other studies, our participants also paid attention to immediate look and feel, design, other people’s star ratings or reviews of apps (*social proof*), and costs during the search [31,35]. However, the considerable focus on the *hidden costs* of free apps (eg, whether paying for an upgrade would get you extra functionality, quality, or just the elimination of annoying advertisements and pop-ups offering upgrades) is something we have not seen in other studies. This insight is an addition to the factors that people consider important during the uptake of apps for smoking cessation.

Furthermore, this study brings nuances to existing insights from the literature regarding the importance of ranking and rating. Previous research [28,29] has shown that apps with (among other things) high rankings and high ratings are downloaded most often. Although the apps chosen in this study all have high ratings and rankings, this was not what participants paid the most attention to during their search. Participants mainly wanted information about the features of the apps that they expected to be fun or helpful. Individuals looked for specific characteristics of an app (eg, functionality, appearance, and price) and simply started at the top of the search results. Therefore, although ranking was seen by a few participants as a useful source of information for selecting apps, the influence of ranking is clearly noticeable, as starting a search at the top of the list is simply convenient and obvious. This leads to the fact that in the Google Play Store, for example, a few dozen apps at the top of the ranking account for almost half of all downloads [54]. More than 85% of all health apps are found much less often, rarely, or never [55]. This is potentially a loss, as that 85% of apps may contain exactly the functionalities and features that someone is looking for.

Third, in our results regarding *information processing and decision-making*, we observed that participants had to make several decisions during the entire process. In addition to smaller ones, such as choosing search terms, every participant chose to click on or skip apps in the list of search results; leave a detailed information screen or download an app; and finally, after downloading, keep or discard it. To make these decisions, participants needed to understand, interpret, and remember information, form a mental picture of smoking cessation apps, and continually adjust that picture based on new information. Furthermore, choosing an app involved thinking about wishes and requirements and formulating opinions about the functions and features of apps. Some participants also imagined what using certain functions would be like for them in practice. Overall, this seemed to be quite a cognitive load, as without the participants realizing it, they often made mistakes in information processing. A number of times, we observed that choices (click, skip, download, or discard) were based on a judgment formed on information that was misread, misinterpreted, misunderstood, or misremembered, ultimately affecting the final choice for an app. These findings are in line with the known deficiencies in human thinking and decision-making [56,57], including, among others, restricted capacity and forgetting [58]. To the best of our knowledge, the influence of errors in information processing on choosing health apps has previously not been explored and recognized in other studies in this field.

Finally, during searching and selecting, we observed *transformations* in the areas of knowledge, wishes and requirements, and confidence in apps. Knowledge increased from *knowing 1 or 2 basic functions* before starting the search to participants feeling they had a full picture of what smoking cessation apps can do and offer. While gaining knowledge, participants developed ideas about wishes (likes or dislikes) and requirements, which were eventually important in deciding which apps to download, discard, or keep. Notwithstanding this development of knowledge, wishes, and requirements, confidence in smoking cessation apps did not vary much if we compare the participants’ estimations before, immediately after, and 2 weeks after the search. For some, confidence was slightly higher immediately after the search, leaving participants rather optimistic. However, that rise was nullified after the 2 weeks of use, with confidence returning to the level before the search or even lower. For some participants, confidence in smoking cessation apps as useful aids had already decreased immediately after the search.

Although it is fully expected that people go through a transformation in knowledge, wishes, and requirements during the decision process [59], to the best of our knowledge, this has not been reported before as an essential part of the search for health apps. We reflect on this in the *Suggestions for Further Research* section. With regard to trust in smoking cessation apps, we confirmed what the study by Regmi et al [60] put forward as a potential threat to smoking cessation apps. In an analysis of strengths, weaknesses, opportunities, and threats, they postulated the loss of trust from users because of the incongruence of perceived app abilities and actual functionalities.

### Strengths and Limitations

This study’s additions to the literature are primarily the result of our application of contextual inquiry, a method that is not often used in comparable studies. By using contextual inquiries, we were able to study the act of choosing an app in a situation as naturally as possible. Participants could search on their own devices at a place and time that was most convenient for them. This may have increased the likelihood that participants would feel at ease, be honest and open, and understand and accurately remember information, which, in turn, would contribute to data quality [61]. Furthermore, the mindset created by the contextual inquiry’s specific basic principles of apprenticeship and partnership facilitated curiosity, humility, interest in, and respect for the respondent, which are generally seen as success factors in conducting interviews [62]. Moreover, close collaboration with participants throughout the research process is thought to produce credible data [63].

We extended our contextual inquiries by video recording the screens of the participants’ mobile devices and audio recording their comments. This allowed us to detect that there is often a discrepancy between what people think they see, read, understand, and remember and what actually is on the screens. These double recordings also enabled us to observe that choices are frequently based on the faulty processing of information.

We also consider the inclusion criteria for our participants as a strength of this study. The inclusion of participants in the study who wanted to search for an app to actually stop smoking caused the respondents to be more invested in the task and made the task less artificial. Making observations of actual, realistic behavior in a natural context may have contributed to the ecological validity of the research [64].

Notwithstanding the strengths of the methodology, our chosen approach also has some drawbacks, which create a number of limitations. First, it has been hypothesized that a good rapport between the interviewer and respondent also has downsides and could result in response bias as it causes respondents to ingratiate themselves with interviewers [61]. This could explain why some of our participants indicated that the search during the contextual inquiry was somewhat different from how they would normally search for an app. At the end of the search, 3 participants (participants 3, 4, and 9) indicated that they had chosen more consciously than they normally would have as they had to state aloud why they made certain choices. Three participants (participants 4, 5, and 9) indicated that they had searched a bit more extensively and thoroughly. For 3 participants (participants 2, 7, and 8), the way they had searched for a smoking cessation app was completely different, as normally they would never browse but just go to the app store for a direct search based on an app name. Lastly, 3 participants (participants 1, 6, and 10) searched and found their app in much the same way they would normally do.

In addition, during analyses of the data, we reached saturation [65] when we got to the point where further data collection would not necessarily add anything to the overall story [66]. Even before the analysis of the tenth and last inquiry, we found no new variants of expressions in behavior within the themes or subthemes. The decision to stop further recruitment was reinforced by the consideration of time investment in recruitment and the chosen methodology. Nevertheless, it is conceivable that studying more people could lead to an even richer description of the search process, people’s actions, and their motivations. For example, one of the participants was diagnosed with Pervasive Developmental Disorder-Not Otherwise Specified (PDD-NOS) and surprised us by looking at the information in a completely different way than the other participants.

A further limitation concerns the composition of our sample. As the aim of the study was to better understand the process of choosing a smoking cessation app, recruitment was not about getting a representative sample but about composing a group of people in such a way that we could gain different perspectives. By purposive sampling on factors that could theoretically influence the app choice, such as gender [67-69], age [70], and education level [71], we tried to do just that. However, it is thinkable that different cultural backgrounds or other personal characteristics could provide different, new insights.

### Suggestions for Further Research

This study raises several new questions. During the search, participants gained knowledge about smoking cessation apps and developed wishes and requirements. This finding implies that the search process in itself plays a role in the uptake of apps. This raises entirely new questions about the influence of these transformations on the outcome of the search process, selecting an app: how do gaining knowledge and developing wishes and requirements shape the decisions people make, is it an important part of the decision process, does it lead to different outcomes than a search in which no transformations would take place, do these transformations also occur in less active and thorough searches, and what underlying mechanisms are at play? For instance, as all our participants chose an app with the intention of using it, an active and thorough search may have contributed to more satisfaction with the choice [72] and lower uncertainty and thus have increased the intention of using the app [73].

Another potentially interesting question is one regarding the effect of the number of presented search results. The Apple users who used a Dutch search term were presented with significantly less relevant results than those who used an English search term or those who searched the Google Play Store. On average, participants who used Apple explored more app information screens than Android users (mean 11.6 vs mean 5.2) and downloaded more apps (mean 2.6 vs mean 1.2). A number of participants indicated that they liked the fact that there were not so many results but were concurrently puzzled by the limited results and presentation of irrelevant apps. Experimental research with more respondents might explore differences in experiences, feelings, considerations, and decisions among various numbers of search results.

Finally, the matter of initial use is intriguing. Much research in the field has focused on understanding the factors that influence uptake, such as what people find engaging. The goal of many of these studies is to increase uptake by helping users to, for example, obtain information about things that are potentially engaging [32,33,74,75]. In this study, we saw that participants searched for the functions and features they liked or found useful and that uptake in the sense of *downloads* was high—every participant ended the search with an app and the intention to use it. However, after 2 weeks, we saw that some of the participants had not even opened the app. Despite successful uptake based on expected engaging functions, initial use was thus not guaranteed, let alone actual engagement and continued use. We suggest that it may be worthwhile to investigate what happens between uptake and initial use. It could be useful if further research takes into account the extra step of initial use between uptake and continued use.

### Implications for Practice

The results of this study indicate the need to work on the forms of decision support in app stores. We propose a number of suggestions for the design of three obvious solutions to support people in searching and selecting a fitting app for smoking cessation: advanced filters, recommender systems [76], and curated portals [35].

The first solution involves advanced options to filter the search results. In an immense supply, where people want information that is not easy to find, if done properly, filters can make a difference in terms of time, energy, and positive search experience [77]. Choices based on popularity and others’ opinions can be made relatively easily by people themselves. Therefore, filters should focus on the content of apps, taking into account the functions and characteristics of the app. With the help of technologies such as natural language processing [78], text analytics [79], and machine learning [80], it is possible to analyze apps in terms of content and identify the characteristics present in the app. Filters and other tools based on the identified characteristics can easily be included in the user interface of an app store, with terms that are relevant, useful, and recognizable to the user, to help the user choose an app that is valuable to them.

The second solution is recommender systems. In this study, we have seen that participants put much effort into figuring out what functions and features they expect will really help them and that they actually find that very difficult to do. Most participants seemed quite unaware of what they needed to support them in their behavior change. Thus, many choices in our study (click or skip, click or download, or keep or discard) ended up being based purely on *a feeling* or on what participants found fun, attractive, or funny. However, there can be a discrepancy between what people indicate to prefer and what actually works well for them [81-83]. The possibility of matching apps and participants with a recommender system could theoretically go beyond matching based on what participants like. Recommender systems could be trained with delayed feedback on the effectiveness of the app on the health behavior change, in this case: smoking cessation. Through this training, the system gradually learns which (functions in which) apps work for whom, optimizing the systems’ recommendations on the expected effectiveness of an app, ultimately helping people to find an app that they not only like but that may also work effectively for them.

A solution in the form of curated portals adds value in yet another way when supporting people in choosing an app. First, we have seen that several participants wanted to find a professional, evidence-based app founded on scientific insights. However, information about the quality of apps is almost impossible to find in the app stores. From earlier studies, we know that high-quality apps are scarce to begin with [22,60,84,85] and, therefore, difficult to find in enormous supply. People for whom quality is a criterion would be helped by reliable assistance in choosing. There are reliable sites that users also trust [31,35], such as the GGD App Store in the Netherlands [86]; however, these are not found by users, as this study and previous research have shown [31,35]. An easy-to-find, well-curated site could also help keep people from giving up after a first tried app. It can be a safe and orderly collection where people can return to try a new app if they do not like the first one.

The second argument in favor of curated app portals is data and privacy protection. As in previous studies [35], we also observed that participants hardly glanced at permissions, privacy terms, and conditions. Although people regularly indicate that privacy and data protection are important to them [31,32], in practice, for most, it is not feasible to process and understand this type of information [87]. Even if consumers were to read the incomprehensible terms and conditions, information could be incomplete [88]. Moreover, it has been found that many apps, both free and paid versions, display dismal privacy practices [89,90]. As the use of apps depends on the acceptance of the conditions, and many people are not (or cannot be) aware of the risks [87,88], a reliable, independent party that monitors privacy and data conditions is of great importance.

### Conclusions

The empirical findings in this study add insights into the literature on the process, information needs, information processing, and decision-making and transformations in knowledge, wishes and requirements, and confidence and trust that occur when searching and selecting apps for smoking cessation. Currently, finding an app that contains functions and features you expect to help you quit smoking takes considerable time and energy and can even be a negative experience. At present, app stores do not appear tailored to finding suitable smoking cessation apps, and consequently, people who want to quit smoking need to process a lot of information and make a multitude of choices. In this entire process, errors in information processing creep into and affect decisions. Furthermore, although every participant downloaded an app with the intention of using it (uptake), initial use was lower, and subsequent continued use and engagement were even lower. As such, our findings highlight the need for further research into the factors that affect initial use and into the relationship between active, thorough searches and uptake and initial and continued use. Furthermore, our findings stress the importance of developing helpful tools to guide users through the immense supply of health apps, such as advanced filters, recommender systems, and curated health app portals. Among other things, we suggest the creation of filters and recommendations based on app functionalities and curated portals to guide people to high-quality and trustworthy apps. These solutions could make the search process easier, faster, and more enjoyable for people who wish to find an app that is valuable to them and ultimately effective.

## Appendix

Multimedia Appendix 1 Example interview guide contextual inquiry.

Multimedia Appendix 2 Example interview guide follow-up interview.

Multimedia Appendix 3 Example process chart.

Multimedia Appendix 4 Checklist Standards for Reporting Qualitative Research.

Multimedia Appendix 5 Search queries.
