# Supplementary material for: Process and Information Needs When Searching for and Selecting Apps for Smoking Cessation: Qualitative Study Using Contextual Inquiry
Source: JMIR Hum Factors. 2022 Apr 14;9(2):e32628. doi: 10.2196/32628 (PMC9052019; doi:10.2196/32628)
Supplement: Multimedia Appendix 1 [file humanfactors_v9i2e32628_app1.pdf]

Example Interview Guide – Contextual Inquiry
[Please note that the original protocol was in Dutch and that this document was translated automatically (using DeepL Translator) for accessible publication purposes.]

[This is a Multimedia Appendix to a full manuscript published in the J Med Internet Res. For full copyright and citation information see http://dx.doi.org/10.2196/jmir.32628]

Who? Adults (18-65) who want to choose an app to help them quit smoking. The participant has their own device, experience downloading apps and speaks good Dutch or English.
When and where? At the participants home, at a time previously agreed upon with the participant.
How? After getting acquainted and discussing the process, we first go through a short questionnaire. Then the participant selects an app (for quitting smoking) on his or her own device. I watch the session and ask questions during the session. The meeting is recorded (audio and screen mirroring/recording) and I also make notes. Once the task is completed, we discuss my notes together (member check). The session ends with a 'formal' conclusion.
What? The focus of the research is: choosing an app (for smoking cessation), and the information (about the app) that is important for people in doing so. We want to see <u>what</u> people do when they choose an app and especially find out <u>why</u> they do it.
About this protocol: Use this protocol primarily as a checklist so you don't forget important process steps and go over all the "business and administrative" issues with the participants. The questions during the interview are for inspiration. It is not a survey!

1. Before the interview

<u>Longer in advance</u>

- Send participant the information letter
- Exchange contact information
- Explaining screen sharing
    - Ask about type of device (tablet, smartphone, laptop), brand (Apple, Samsung, ...).
    - Explain what is needed (depending on device): install Mobizen Mirroring App on participant's device and/or access to wifi.
    - Ask for permission
    - Use set text (see mail)
- Check by mail:
    - Participant wants to quit smoking, wants to do it with an app and wants to figure out that app with you. Is that right?
    - Can you download apps independently?
- Make an appointment

<u>Day in advance</u>

- Confirm appointment
- Getting things ready - checklist:
  ☐ Interview script
  ☐ Consent form for participants
  ☐ Information letter for participants
  ☐ Laptop (charged) & Mobizen installed (bring cable)
  ☐ Digital voice recorder (and extra batteries)
  ☐ Notepad and pens
  ☐ Agenda for planning telephone interview
- Planning route and travel time

2. Explanation (10 minutes)

Content explanation of the study

- This research is part of a bigger picture: the ultimate goal of the "bigger" research is to find ways to help people get health apps (for quitting smoking, or exercising more, or eating healthier) that are a good fit for them.
- Were you able to read the information letter?
- In this research, we want to discover what considerations people make when choosing a health behavior change app from an app store, because we don't really know much about that yet. Today, I'm particularly interested in finding out what you want to know about an app to determine if it's one that's "right" for you: what information will you look at, can you find the information you'd like, and so on. I am also curious about how you experience the process of choosing; whether it is fun, easy or difficult.
- My personal motivation: I find it annoying to see when people who have finally found the courage to quit become demotivated or disappointed because an app does not do what they hoped or expected it to do, and therefore stop quitting.

Procedural explanation of the study

- The survey consists of two parts: the interview with choosing an app (which we are doing today) and in about a week and a half another short phone interview in which I want to ask you some questions about how you experienced the app - whether the app actually matches what you expected when you chose it.
- Today, we're going to start with a short questionnaire, with some questions about your personal situation such as your desire to stop smoking. After that you are going to choose a "quit smoking app", where I would like to know continuously what you do and especially why. I think it will take us about an hour and a half.
- But before we can start, we have to discuss a few 'business' matters, such as consent to participate in the study.
- If you have any questions: don’t hesitate to ask them!

Explanation of consent form

- In order to participate in the study, you need to give official permission. You can do this by signing this form.
- Go through the form - explain each sentence
- Do you have any questions about this?
- Sign the consent form

> If in the unlikely event that the respondent does not wish to sign the consent form, thank the person for their time, leave the information letter, consent form and your contact details. Indicate that if she/he has second thoughts, you would be happy to come back at a later time to do the interview.

Explanation about recording

- In order to be able to work at a later time with everything we have discussed today, I would like to record our conversation on this recorder.
- I'm going to type out the recordings verbatim, in order to properly analyze what people look for when choosing an app. I will leave out all names and personal details, and use numbers instead. Only I know which number belongs to which person. After I have typed out an interview (and the text is securely stored) I delete the recordings. The anonymized typed-out interviews are kept in the university's (digital) vault until the end of the research.
- Do you have any questions about this?
- Do you agree that we record the interview? [yes/no]

Explanation of screen sharing (has already been explained prior to the survey)

- To make it easier for you to watch while choosing an app, I would like to connect your device (with a wire | via the wifi) to my laptop and use a program on my laptop to watch what is happening on your screen.
- I will also record these images so that I can look back at them later, should I need to. Again, this is completely anonymous and the images are erased once my research is complete.
- I only see what you are currently doing on your smartphone/tablet and therefore have no access to your device. Screen sharing is otherwise completely secure.
- Specifically, it means:
    - That you must give me access to your wifi network AND/OR
    - That for the duration of the interview we need to install an app on your phone (with the necessary permissions for the app)
    - We can do that together so you can see what I'm up to. And afterwards, I'll 'forget' about your wifi network and we'll uninstall the app immediately.
- Are you okay with sharing our screens? [yes/no]
- Ask where the participant normally sits with her/his mobile device. Then take the survey there as well.
- Connect participant's device to my laptop (screen sharing) and briefly show what I see.
- Have the participant log into the app store before turning on the screen recorder.

3. Warming up (20 min.)

The purpose of the warm up is literally to warm up - to get used to each other and get into it a little bit together. In addition, it is important here is to find out if the participant has expectations about, or hopes for things from the app. This could be based on:

- experience with (help with) smoking cessation
- apps (or eHealth interventions) for smoking cessation
- other eHealth intervention for other 'health behaviors
- thoughts about the app to be chosen

Focus = Hope (what does the participant hope the app will be able to do, based on past experience or otherwise)

That was the business and administrative part - now we can really start the research. We'll start with a short questionnaire to find out a little more about you. I will now turn on the recorder.
> **Turn on voice recorder**

Background information about the participants:

- Can you tell us a little about yourself? Age, education, work? How long have you been smoking? How much do you smoke?

Previous quit attempts

- Have you ever made a quit attempt? When was that? How did it go then?
- What helped you to quit? What did you like about quitting smoking at that time? What was difficult? What made you start smoking again?
- Have you ever used an app to stop smoking? Or other eHealth applications?
  o Which app was that? Several?
  o How did you get that app? (Searched in app store, recommended by friends, etc.).
  o Do you remember why you chose that app at the time?
  o How did you like using that app?
  o What did you like? What really helped you?
  o What did you not like? What didn't help you?
  o Why did you stop using that app?

- Besides the quit attempt with that app / eHealth intervention we just talked about, were there any other quit attempts in the past?
- Have you ever used any other kind of app for your health? For example, for running, or eating healthier?

Experience with apps

- Do you download apps often? How often do you go to the app store to download an app?
- When was the last time you did so? What kind of app was that?

Motivation to quit smoking - Can you rate that on a scale of 0 to 10...?

- How important is quitting smoking to you right now?
  0 = not important at all, 10 = the most important goal in your life.
- How "ready are you" to quit smoking in the next month?
  0 = I am not ready at all, 10 = I am 100% ready
- How confident are you that you will quit smoking in the next month?
  0 = Not at all sure, 10 = 100% sure

Expectations of the app to stop smoking

- What do you expect from the app? What do you hope it will do to help you quit smoking? What should it be able to do? When is an app a good fit for you?
- What do you think you need to stop smoking? What would help you stop smoking? What don't you think would help you at all?
- What were things in the past (if app or eHealth used) that helped you? What were things you liked? What did you not like?
- How much confidence do you have that an app will help you quit smoking?
- [Scale of 0 to 10]
- How important is it for you to make a good choice today? [Scale of 0 to 10]

3a. Contextual Interview (35 min.)

The focus here is: what do people pay attention to when choosing an app, and why do they pay attention? What are the considerations for clicking things, viewing things, choosing things, downloading things? What do they want to know about an app to decide whether to use it? What are the information needs? In addition, there is the actual process - how does someone approach it; what does the person do?

Transition

Now we come to choosing an app for smoking cessation. The idea is that you choose an app as you normally would if you wanted an app of one thing or another. You do it all your way: on your own device (for example, your smartphone) and in the app store where you normally choose apps (for example, the Apple App Store or the Google Play Store). Take your time to look at and read the things you want.

While searching for an app, I'll be watching and asking you questions. There are no right or wrong answers. It's about how you choose an app. I especially want to understand what you do and why you do it that way.

Take your time to choose an app. The task is complete when you have found an app that you really want to use. It can also be that you can't find a suitable app; that there is no app that 'fits' you. If at some point, after searching for a while, you don't think there is anything there to help you stop smoking, then don't. Downloading is not an end in itself. At the end, we will have a brief discussion together about my notes to check if you recognize yourself in the things I have seen, heard and written down.

I am especially curious about why you do certain things, so I will ask about that in between. Together I want to try to discover what kind of information you are looking for and how you experience the search process. It can help if you think out loud. Shall we begin? If you log in to the App Store, I'll turn on the image sharing and recording.
> **Turn on Mobizen Mirroring and PowerPoint screen recorder**

Choosing the app

The **focus** while choosing the app is the **WHY**. What is happening in a person's mind? Why is this person doing/looking/reading/clicking/reacting in this way? Pay attention to the following things:<br>• What information do people look for and view when choosing an app?<br>• What information would they want?<br>• What do people do? What do they click on, what do they look at, what are they looking for, what are they hesitating between, why are they sometimes quick to click/decide and other times slow, what do they find difficult/difficult/frustrating/pleasant? And why?<br><br>> Write down things you see/notice that you want to come back to later.<br>> As soon as someone lands on a page, indicate what you want to know about the participant. So before someone goes to view the page.<br>> If all goes well, during the warm-up you will have identified aspects that the participant hopes to find in an app, based on:<br>- past experiences with smoking cessation<br>- apps or mobile interventions for smoking cessation<br>- other types of health apps<br>- thinking about functionalities and wishes<br>Does the participant search for information on these aspects? Does the participant make new discoveries during the search process about what she/he thinks is nice and/or useful about an app? Make a note of these aspects and ask further questions about them in breadth (are there other aspects) and depth (hope/expectations, nice/useful).<br>> Pay attention to (and react to) little things: "huh?", "this is crazy", facial expression, looking at something for a long time/longing, etc.

Can you show me how you go about choosing an app for smoking cessation?

Process steps:

1. Opening app store / first search (search function / browsen / …)

Before you go on and click on anything: can you tell me what you notice on this 'page'? What catches your eye? What catches your attention? What do you want to know more about? What do you want to click on?

- What are you looking at? What do you think about that?
- What catches your eye? What do you think is important?
- What do you want to find out? What are you looking at to find out?

2. List of search results

Before you go on and click on anything: can you tell me what you notice on this 'page'? What catches your eye? What catches your attention? What do you want to know more about? What do you want to click on?

- What are you looking at? What do you think about that?
- What do you notice? What do you think is important?
- What do you want to find out? What are you looking at to find out?
- What would you like to know about the app, but can't find?

- What is your feeling when you look at these results? Why?

3. Detailed app information screen

On a detailed app information screen, different 'types' of information can be found:

- 'Entry' (on smartphone you can immediately see: logo, title, developer, PEGI and summary #reviews/ratings/downloads)
- Screenshots (images, you can scroll to the right for more)
- Description (a few lines, with possibility for the full text by clicking 'Read more')
- Ratings and reviews (the distribution of reviews, a few reviews are shown with possibility for all reviews by clicking on 'View all reviews', in the Google Play Store filters are present)
- Other (e.g. 'App info', 'Permissions', 'Developer info')

Before you go on and click on anything: can you tell me what you notice on this 'page'? What catches your eye? What catches your attention? What do you want to know more about? What do you want to click on?

- What are you looking at? What do you think about that?
- What do you notice? What do you think is important?
- What do you want to find out? What are you looking at to find out?
- What would you like to know about the app, but can't find?
- What is your feeling about this app? Why?

4. Back to results & new detailed app information screens

Before you go on and click on anything: can you tell me what you notice on this 'page'? What catches your eye? What catches your attention? What do you want to know more about? What do you want to click on?

- What are you looking at? What do you think about that?
- What do you notice? What do you think is important?
- What do you want to find out? What are you looking at to find out?
- What would you like to know about the app, but can't find?
- What is your feeling about this? How did that come about?

5. Downloaden / installeren

You are now installing this app.

- Why did you choose this app?
- What do you think you like about this app?
- How do you think this app will help you quit smoking?
- Do you have any particular doubts about this app?

6. Opening the app and the final choice

Exploring an installed app is also still part of the search process. When the participant opens the, just let her/him go for a while. The **focus** here is on the **REALIZATION** of the **EXPECTATIONS** the participant had about the app based on the information in the app store. Does the first impression of the app match what the participant had expected/hope for about what she/he liked and/or found useful about the app?

> Do not go into the functionalities of the app, except to find out if everything the participant had hoped for (the different aspects) was found. Is there more? Is it bad that some things are not there?

After opening and exploring the app for a while

- Is it as you expected? Does the app match the image you had when you just saw it in the app store?
- What is your feeling about this app now? Why?
- You're now getting all excited about..., did you notice that when you were searching?
- Do you now see things in the app that you didn't expect beforehand?

The task is accomplished once the participant has chosen an app that she/he feels is the one that is going to help with smoking cessation; that she/he has made a good choice; that this is an app she/he wants to use.

After final choice

- You have now decided to use this app, do you feel it suits you? Why?
- Do you feel you have made a good choice? What was the deciding factor in making the choice? Why did you (finally) choose this app and not one from another?
- What were things you discovered/learned through looking at the apps (in the app store) about what you think is important about an app to help you quit smoking?
- Based on past experience (with other apps or smoking cessation), what were the things you expected or hoped for from the app?
- Did you feel you got the right information (in the search process) that you needed to find a good app?

3b. Contextual Interview – the wrap-up (20 min.)

During the wrap-up, we use the notes taken to summarize in outline what you saw and heard, and to discuss the interpretations made. In addition, this is the time for additional questions and to discuss any ambiguities.

- That was it! Thank you for wanting to do this with me. How did you like it?
- Let's see if we can summarize what I saw and heard, and if that's accurate as far as you're concerned....
  - PROCESS You chose the app by... (using the search function | Googling | browsing the app store | ...)
  - INFORMATION SOURCES You made the choice by considering... (reviews | rating | descriptions | ...)
  - DESIRES / EXPECTATIONS (per aspect) You found it important to know that the app ... (is free | well reviewed by others | looks nice | ...). You found that important to know because....
  - You were specifically looking for information about it and were able to find it (or not).
  - You got a certain image of the app while searching and exploring, and expect it to... (motivate you | inform you about health | calculate how much money you save | ...)
  - You sometimes felt while searching/choosing the app... mainly because...
- At the beginning of the interview, you indicated that you have [#] confidence that an app will help you quit. How much confidence do you now have that this app will help you quit smoking? On a scale of 0 to 10.
- On a scale of 0 to 10, do you plan to use your chosen app in the next week?
- Had you thought about choosing an app beforehand? Had you searched for apps beforehand, in "preparation" for this study?

4. Completion (5 min.)

- It's not a big deal if you're not going to use the app next week - do what you would do if you weren't participating in this study. What I mean above all is: don't do it for me, but only for yourself, because you really want to use the app.
- Explaining the follow-up research and making an appointment for a telephone interview
- If you suddenly think of anything you would like to say later on, or have any questions about anything, please let me know. You can call or e-mail me - you have my contact details?
- If you know anyone else who 1. wants to stop smoking, 2. wants to do so with the help of an app and 3. wants to find out about this app with me, I would be very grateful!

5. After the interview

- Process notes immediately after each session and adjust protocol if necessary
- Write a logbook / field notes (Evernote)
- Save recordings
- Scan and store signed consent form
- Update 'key table
- Typing out recordings

Appendix I - About Contextual Interview & Inquiry

> *Running a good interview is less about following specific rules than it is about being a certain kind of person for the duration of the interview. The apprentice model is a good starting point for how to behave. Then the four principles of Contextual Inquiry modify the behavior to better get design data: context, go where the work is and watch it happen; partnership, talk about the work while it happens; interpretation, find the meaning behind the customer's words and actions; and focus, challenge your entering assumptions. If all these concepts start to become overwhelming, go back up to the higher level idea of apprenticeship. You want the attitude of an apprentice; you want to create an intimate relationship in which you and the customer collaborate in understanding their work, using your focus to help determine what's relevant. That's enough to run a good interview.*

The method used in this study is called a Contextual Interview. This is a term from the Contextual Design world, where the Contextual Interview is a part of the 'Contextual Inquiry'. Or as Beyer & Holtzblatt (1998), the founders of Contextual Inquiry, put it, "The most common structure for Contextual Inquiry is a contextual interview."

Designers and researchers try to get a grip on how people (mostly in companies) do their work in a Contextual Interview. Usually, the method is used to fully understand the use of a particular system. That is, literally, how a person uses the system for his/her work, at his/her job, in the context where all kinds of other things are also happening.

The Contextual Interview basically means that the researcher/designer (by appointment) goes to the employee's workplace and observes the person's work. In between, questions are asked to understand why the employee does certain things (with or without the system in question).

A Contextual Interview is generally composed of four parts (Beyer & Holtzblatt, 1998):
1. **Introduction** (15 minutes) - You introduce yourself and your focus, you promise confidentiality, get permission to tape, and start the tape recorder. Explain that the customer and her work is primary and that you depend on the customer to teach you the work and correct your misunderstandings.

2. **The transition** (1 minute)
The interviewer states the new rules for the contextual interview-the customer will do her work while you watch, you will interrupt whenever you see something interesting. Here, you want to create the new rules for the contextual interview, so you state them explicitly.

3. **The contextual interview proper:**
The customer starts doing her work task, and you observe and interpret. You are…

- the apprentice, observing, asking questions, suggesting interpretations of behavior
- keeping the customer concrete, getting back to real instances
- taking copious notes by hand
- nosy

The principles of context, partnership, interpretation, and focus guide your interaction during the interview. (zie PDF ‘Tips for conducting a successful Contextual Interview - Holtzblatt et al., 2005’)

4. **The wrap-up** (15 minutes)
Skim back over your notes and summarize what you learned, trying not to repeat verbatim what happened, but saying what is important. This is the customer's last chance to correct and elaborate on your understanding.

In this study, we want to find out what considerations people make when choosing an app for smoking cessation. How do people decide which app to download? What information do they look for? What information do they use? What would they like to know about an app before downloading it? What do they think the app can do for them? And so on. Beyer & Holtzblatt (1998) call this type of task (choosing an app) an 'internal task' and state that the Contextual Interview is a good method for this.

> *Sometimes the inquiry needs to focus on internal mental processes, such as how decisions are made. In this case, the interviewer must be present when the mental process is happening because there's no way to recover enough in a retrospective account. You may need to create events that will cause the mental process to happen so that you can be present. Then interrupt a lot; make a lot of hypotheses about what the customer is taking into account in their thinking. Warn the customer this will be very disruptive, but as long as the customer has to make the decision, they will keep working through it and you will learn something about how they do it.*

What distinguishes a CI from 'observations and interviews' is first that it takes place in the natural context of the person (and not in a lab or imaginary setting) at the moment the task is done. This allows you to literally watch while someone performs a task, and ask questions while the task is being done, thus eliminating recall and reporting bias. You can ask further questions about actions in the moment and thus go deeper into the 'why' of the actions.

In addition, the researcher forms a so-called partnership with the person who is being observed/questioned. The research is really done together with the respondent. The role of the researcher is that of 'apprentice' - the respondent is the 'expert'. This also ensures that the researcher, more than in traditional forms of (observation and interview) can share his or her interpretations of what has been observed. It is recommended that in a contextual interview, you literally tell the participant what you think motivates him/her to do something. The participant's response then confirms or refutes that interpretation, yielding new insights. In more traditional forms of observation/interview, this would likely be seen as "too controlling," or something you are using to influence the participant.

Finally, a characteristic of a Contextual Interview is that the 'findings' are discussed with the respondent immediately after the observation/interview in order to give meaning to the findings together. The member check thus takes place on the spot.

We expect the Contextual Interview to be a good method for answering the research questions[1] because we are looking for underlying motivation and information needs and because we want to see what available information people use. Purely quantitative analysis of online search behavior (in an app store) gives an incomplete picture because nothing can be concluded from that about the use of, for example, the content of descriptions or reviews of an app. Moreover, the question of "why" cannot be answered in this way at all. Looking at the task 'in the moment' and asking questions makes reflecting on the task (and the underlying motivation and wishes) easier than if we ask someone to imagine how they would choose an app.

The main difference with situations in which a CI is 'normally' used is the complexity of the task, and the context in which the task is done. CI is mainly used for research into the use of a system in a work process within organizations, by different people with different roles and ways of working. The subject of this study (choosing an app for smoking cessation) is considerably simpler in that respect. Nevertheless, it would be nice if through this method we could also gain

[1] 'What is the process of searching for a smoking cessation health app in an app store and what information is important to people in making a good choice? What information do people use when choosing an app; what information would they like to use; and is there a discrepancy between the two?'

more insight into the full context (in which such an app is chosen) - the role of (significant) others, of timing, of motivation (in the choice process and considerations).

Literature

36. Beyer H, Holtzblatt K. Contextual Design: Defining Customer-Centered Systems. Amsterdam: Elsevier; 1998. ISBN: 0080503047.

45. Boudreaux ED, Sullivan A, Abar B, Bernstein SL, Ginde AA, Camargo CA. Motivation rulers for smoking cessation: a prospective observational examination of construct and predictive validity. Addict Sci Clin Pract 2012 Jun 08;7:8 [doi: 10.1186/1940-0640-7-8] [Medline: 23186265]

46. Holtzblatt K, Wendell JB, Wood S. The contextual inquiry interview. In: Rapid Contextual Design. San Francisco, CA: Morgan Kaufmann; 2005:79-100.

Appendix II - Examples of functions in an app that can be beneficial to people:

- Personalized feedback and support
- Counters, such as the number of cigarette-free days, the number of cigarettes not smoked, or the amount of money you saved by not smoking
- Motivation, such as providing motivational messages
- Information, such as effects of quitting smoking on health/body, or the process of withdrawal
- Distraction, games or messages that can be evoked at difficult moments
- Rewards, compliments and 'medals'
- Social sharing, such as features to share progress via Twitter or Facebook
- Hypnosis
