# Supplementary material for: Process and Information Needs When Searching for and Selecting Apps for Smoking Cessation: Qualitative Study Using Contextual Inquiry
Source: JMIR Hum Factors. 2022 Apr 14;9(2):e32628. doi: 10.2196/32628 (PMC9052019; doi:10.2196/32628)
Supplement: Multimedia Appendix 2 [file humanfactors_v9i2e32628_app2.pdf]

Example Interview Guide – Follow-up Interview
[Please note that the original protocol was in Dutch and that this document was translated automatically (using DeepL Translator) for accessible publication purposes.]

[This is a Multimedia Appendix to a full manuscript published in the J Med Internet Res. For full copyright and citation information see http://dx.doi.org/10.2196/jmir.32628]

Who? Adults (18-65) who want to choose an app to help them quit smoking. The participant has their own device, experience downloading apps, and speaks good Dutch or English. The participant has participated in the Contextual Interview at t1.
When and where? At the time agreed upon with the participant following the Contextual Interview.
How? Semi-structured telephone interview in which we want to ask specific questions about the choice made in the light of the app chosen. We leave room for other things the respondent wants to tell about the process, the app or participation.
What? The aim is to relate the choice made to the information the respondent was looking for while making the choice. We want to find out how "good" the participant thinks her/his choice is, given the image she/he formed of the app beforehand (based on the information available while choosing the app).
About this protocol: Use this protocol primarily as a checklist so you don't forget important process steps and go over all the "business and administrative" issues with the participants. The questions during the interview are for inspiration. It is not a survey!

- In the telephone interview after one week, the **focus** is on the REALIZATION of the EXPECTATIONS the participant had about the app based on the information in the app store, based on user experience. After a week of use, how does the app compare to what the participant knew/thought/felt/hoped/expected at the time of choosing the app?
- Attention to things he/she did not expect (positive and negative)
- Not going into functionalities of the app, except to find out if everything the participant had hoped for from it (the different aspects) was found. Is there more? Is it bad that some things are not in there?

1. Prior to the interview

- Confirm appointment
- Getting things ready - checklist:
    - Telephone Interview Protocol
    - Digital voice recorder (and extra batteries)
    - Telephone Pickup Microphone
    - Notepad and pens
    - Charger/full power bank for phone (just in case)
- Quiet room reservation
- Read through notes on the Contextual Interview (take notes if necessary)
- This second interview gives you a chance to come back to things that were said or happened in the first interview, or things that you still want to ask the participant after analyzing other interviews. Write these questions down and ask them during the telephone interview.

2. Explanation

- The other day we 'together' picked an app for quitting smoking. We're two weeks in now and I'm curious to know how it went. If you've used the app, I'm particularly interested to

know if it lived up to any expectations you had of it at the time you picked it. Now that you've used the app for a while, how do you look back on your choice and whether you would choose differently? We'll talk about that later.

- Just like the interview, I'd like to record this conversation again in order to work it out later. Is that okay with you?
- Do you have any questions?

3. Interview

<table>
<tr><th colspan="2">Opening</th></tr>
<tr><td colspan="2">• Summarize Contextual Interview
• You were hoping of the app …:
• Important = , Readiness = , Confidence =
• Process:
• What I noticed during the search was…:

Vragen:
• What do you remember about choosing the app? (thoughts, feelings, impressions)?</td></tr>
<tr><th colspan="2">Using the chosen app</th></tr>
<tr><td>Possible questions

• How did you do with the app last week? Did you use the app?
• Which app was it again?
• Did you explore the app(s) further first (after the interview) to decide if you really wanted to use it?
• How often did you open the app?

<u>If the app was not used:</u>
Why not? What was disappointing about it? Did that surprise you? Did you expect that based on the information you had while searching? Do you think there is another app that could help you? If you were to search for it, would you look for different things?</td><td>Background - try to get a grasp on...

• To what extent has the person used the app?</td></tr>
<tr><th colspan="2">Satisfaction with the chosen app</th></tr>
<tr><td>Possible questions

• How did you like using the app? What did you [like/dislike] about it?
• Would you recommend this app to others? Why (not)?
• If you would like to let others know something about the app, what would you say it would be? Is it something you read about yourself when you chose the app?
• How do you feel about the choice you made?</td><td>Background - try to get a grasp on...

• To what extent does the participant feel she/he has made a good choice?</td></tr>
</table>

<table>
<tr><th colspan="2">Reflection on choice process (information and process)</th></tr>
<tr><td>Possible questions

• Does the app do / motivate / help you in the way you expected when you chose it?
o Did you see the things you thought were important when you chose it? Did you see things in the app you didn't expect?
o Did you have a good impression of the app when you chose it?
o Did you have the right information? What is different from what you expected?
• You indicated when you chose the app that you hoped the app would [...]. Was that indeed in the app?
• Now that you've used the app for a few weeks, what do you think (looking back on the search process) of the information in the Google Play Store?
• Is there anything you would have liked to know about this app beforehand (that you didn't see/find when choosing)?
• With the knowledge you now have of the app you chose - would you make the choice differently now? Would you pay attention to different things? Would you look at different things?
• Would you (with what you know now) choose a different app? If so, what would you want to know about that app?
• Do you think you will continue to use the app?</td><td>Background - try to get a grasp on...

• How does the participant look back on the choice process in light of the app chosen?
• To what extent does the chosen app match the image the participant had of it (based on the available information, both in terms of content, and presentation) at the time of choosing?</td></tr>
<tr><th colspan="2">Lastly</th></tr>
<tr><td colspan="2">• Last time, at the end of our meeting, you expressed [...] confidence that this app was going to help you quit smoking (you gave that "a [...]"). How high is that confidence now that we are [one/two weeks] on?</td></tr>
</table>

4. Completion

- Closing and thanking
- Summarize what the participant has said (main points) - is it true?
- Tell the participant there will be a small gift in the mail as a thank you - Allen Carr's book 'Quit Smoking' (if desired, send along the 'Quit Smoking' information sheet).
- If you think of anything you would like to say later on, or if you have any questions, please let me know. You can call or email me.

5. After the interview

- Process notes immediately after each session and adjust protocol if necessary
- Write a logbook / field notes
- Save recordings
- Typing out recordings
- Send the presentation

Appendix I - Tools (interview techniques)

- Depth questions: 'probes' (when, why, how did that happen, what does that show, can you give an example of that?)
    - Can you tell a little more about that?
    - Do you mean that ... (summarize what someone said)
    - Why, how so?
    - Why is that important?
    - Can you give an example of that?
    - Just now you said [...] - can you elaborate on that a bit more? Why is that [...]?
- Width questions: 'prompts' - always keep asking: is there more? Are there any other things?
- Encouragements: Briefly summarize | Silence | 'hm, hm', okay... | Repeat what someone said in questioning form
