# Supplementary material for: Process and Information Needs When Searching for and Selecting Apps for Smoking Cessation: Qualitative Study Using Contextual Inquiry
Source: JMIR Hum Factors. 2022 Apr 14;9(2):e32628. doi: 10.2196/32628 (PMC9052019; doi:10.2196/32628)
Supplement: Multimedia Appendix 3 [file humanfactors_v9i2e32628_app3.pdf]

#app	Which screen?

[**Please note** that the original texts were in Dutch and that this document was translated automatically (using DeepL Translator) for accessible publication purposes. Some of the subtleties may have gotten lost in translation. For practical reasons, only the most important pieces of text were translated, so parts are still in Dutch.]

[This is a Multimedia Appendix to a full manuscript published in the J Med Internet Res. For full copyright and citation information see http://dx.doi.org/10.2196/jmir.32628]

Screenshot

Sees/reads	Says	Information Cue
What does the respondent read aloud?<br>What does the respondent name?<br>What does the respondent interact with?	Comment/idea on what stands out?<br>What does the person think about it? (opinion)<br>What is the reaction to it? (behavior)<br>What is the feeling about it? (emotion)	Which information cues were looked at?<br>• Rating<br>• Ranking<br>• Description<br>• Reviews<br>• Etc.

Does	Reason
What is the follow-up action?	Why does the person do/say that which he/she says/does?

Functionality App	Remark researcher
What functionality of the app was discussed?<br>• Counters (time, money, cigarettes)<br>• Social media<br>• Health information<br>• Etc.	For example, "emotions in voice," all in memo "What does the respondent mean?

Search function

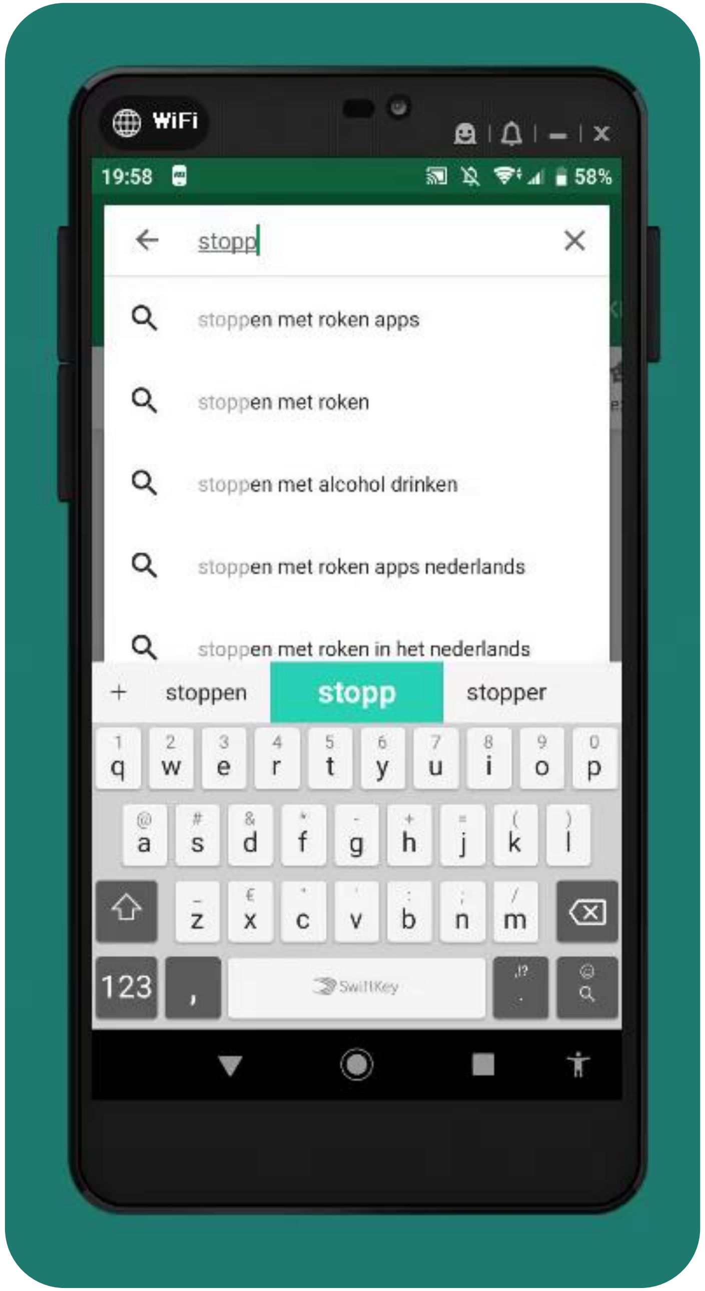

Ziet/leest	Zegt	Information Cue
Autosuggestions	–	–

Doet	Reden
Clicks on (1st) autosuggestion 'quit smoking apps'	–

Functionaliteit App	Opmerking onderzoeker
–	–

1	List of search results

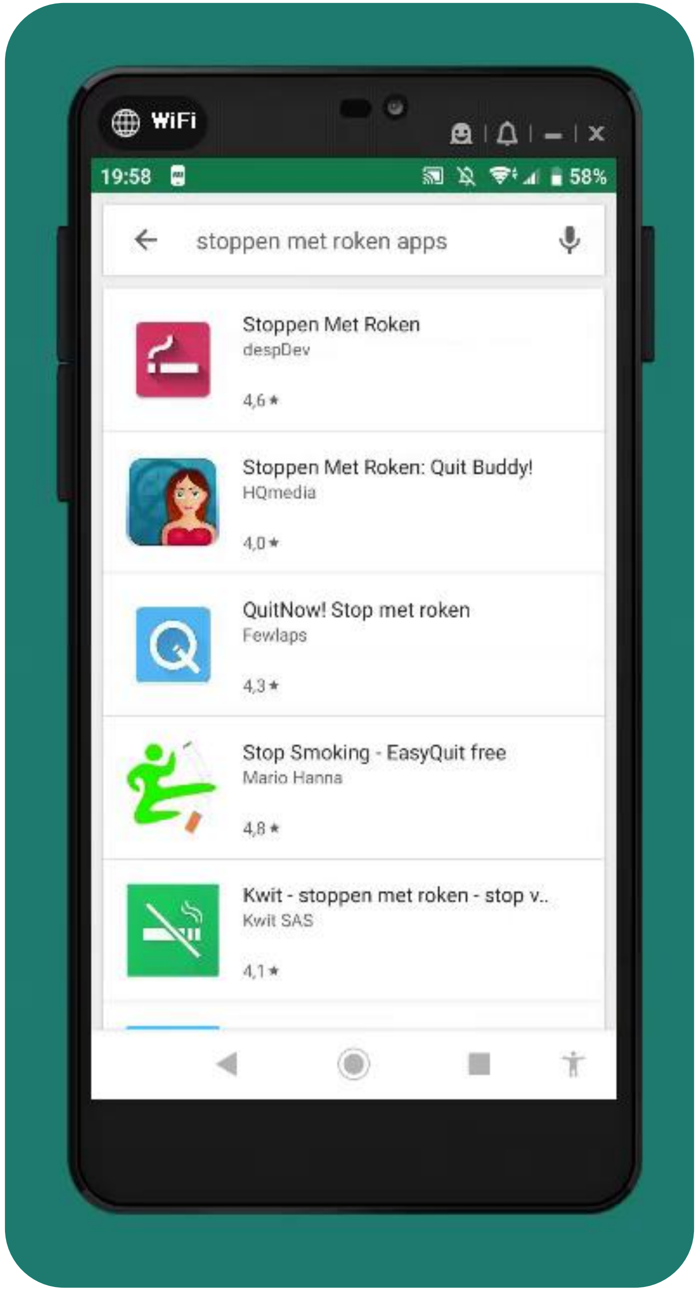

	Ziet/leest	Zegt	Info Cue
1	Results 1 t/m 5	"I think you have a whole bunch of them, I think." "Yeah, they're all just icons, of course." "I always find a funnier icon more fun to look at. The little man here, this one, the green one, kicking his cigarette away [laughs loudly] that I would click on before a female with Quit Buddy. Um yeah, I don't really know about that then."	Icoon app
2	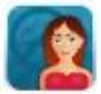	"[...] who is then going to ask you so very kindly all the time, I think, or at least, all the time "well done," all the time of those motivational things. I wouldn't think very well of that, I think. But that's my first insight." [I: Okay, so you're actually telling something that you don't think fits you very well]. R: No, that might be true then, yes. Someone who is then so motivated all the time patting you on the back, of "yeah, you're doing good", and you're.... [laughs]	Icoon app
3	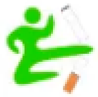	[I: Okay, and that other icon, the green one?] R: Yeah, I don't know, that one kicks your ass, I guess. [laughs hard] [I: And you have to laugh more at that one too] R: Yes, I have that one then. That's funny, with an icon that you then have an association with. [I: Funny?] R: Yes, or funny and fun at the same time, because that appeals more to you than a woman who is standing there with a text from QB.	Icoon app
4	"[...] and then you see those stars"	"[...] but of course that's all pretty high I see, so that doesn't say very much yet. Yes, the figures then actually, but that's of course with an asterisk." "It is now all above four, I see. So then I don't know if I can really get any information from that."	Rating
5	<-	[I: Are there things you actually want to find out about those apps right now?"] "Well, well what they do. That is." [I: Now if you could do anything, unlimited through the screen or whatever, what kind of information would you want to see here so you can make a good choice?] R: Yeah, maybe a little summary, or 'n, yeah.... In one sentence you can already say a lot, I think [I: About?] R: How they operate. They won't all work the same, of course.	Korte beschrijving
6	Easy Quit	[I: Are you missing things on this page? Are there actually things you would like to see on that app right now? With a search overview like that]. R: Yeah, maybe, yeah.... I don't know how that works with apps then... I've never actually paid attention to that, that if there is one more line... Yes, here it says "Easy Quit" or... They could, of course, already pack in one sentence what they do, or how they go about it. Yes, how they go about it. Yes, I don't know at all at this point, of course, what they... how they work. But then you could, of course, in one line, you could...	Naam app
7	Quit Buddy	It says here, of course... 'Quit Buddy', of course is something where you think of, that must be somebody, must be a friend who then goes along....	Naam app

Doet	Reden
Clicks on fourth app in list (Stop Smoking - EasyQuit Free, #1)	"I had just clicked on one because I see a funny little puppet there."

2 List of search results

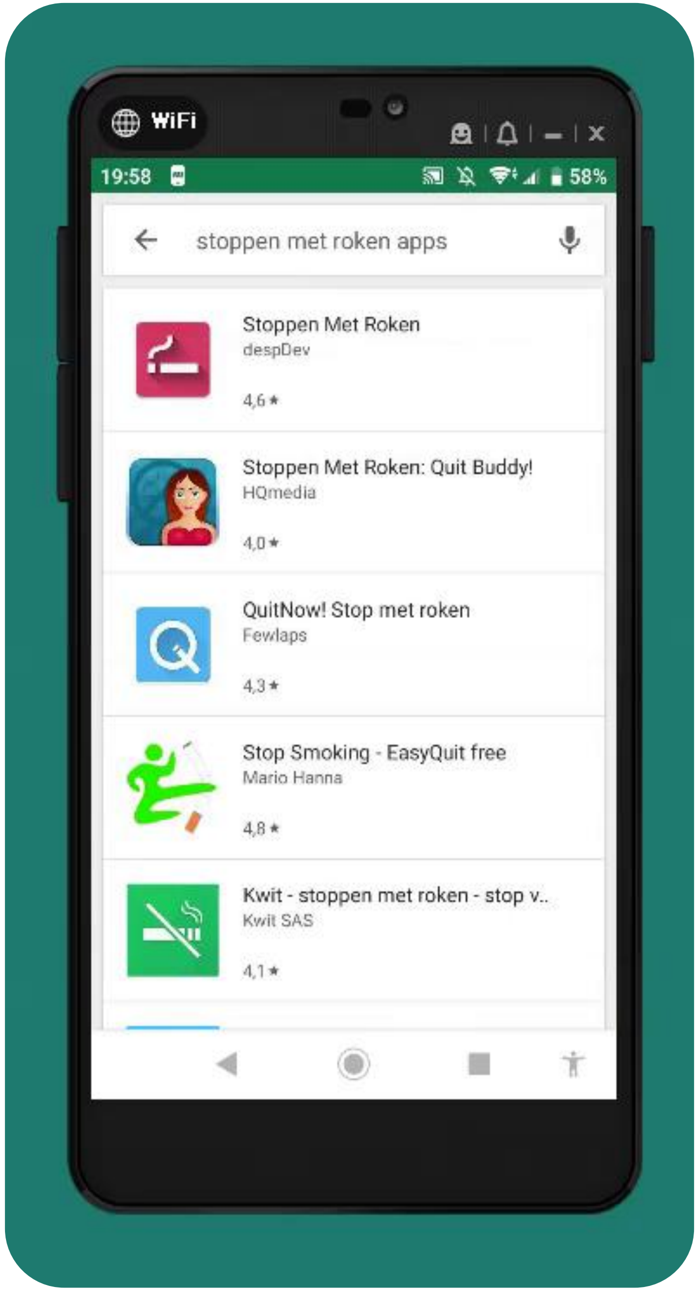

		Herhaling van vorige sheet	
2	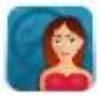	"[...] who is then going to ask you so very kindly all the time, I think, or at least, all the time "well done," all the time of those motivational things. I wouldn't think very well of that, I think. But that's my first insight." [I: Okay, so you're actually telling something that you don't think fits you very well]. R: No, that might be true then, yes. Someone who is then so motivated all the time patting you on the back, of "yeah, you're doing good", and you're.... [laughs]	Icoon app
3	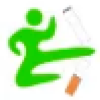	[I: Okay, and that other icon, the green one?] R: Yeah, I don't know, that one kicks your ass, I guess. [laughs hard] [I: And you have to laugh more at that one too] R: Yes, I have that one then. That's funny, with an icon that you then have an association with. [I: Funny?] R: Yes, or funny and fun at the same time, because that appeals more to you than a woman who is standing there with a text from QB.	Icoon app
7	Quit Buddy	It says here, of course... 'Quit Buddy', of course is something where you think of, that must be somebody, must be a friend who then goes along....	Naam app

Opmerking onderzoeker

Here, based on an icon and the name of an app, the participant immediately has a certain feeling about two apps. So the participant:

- Sees something (fairly superficial information cues icon and name)
- Based on that, he immediately forms a certain expectation about 'how the app works' (motivation for example)
- Then he thinks / has opinion about whether it suits him, and
- Decides whether it is for him or not.

While his first impression of the two apps (based on icon and name) may be completely correct (we don't know), it may also be the case that his impression is completely INaccurate. Perhaps the Quit Buddy app is a much better fit for the participant than the EasyQuit in terms of how it works and its approach, but he never finds out because an icon and a name of the former already give him 'a bad impression' (where bad = 'doesn't fit me').

NB By the way, more respondents said something about the Quit Buddy icon - it was (because of that?) one of the most striking apps in all the searches. @Check.

1.1	Detailed app info screen

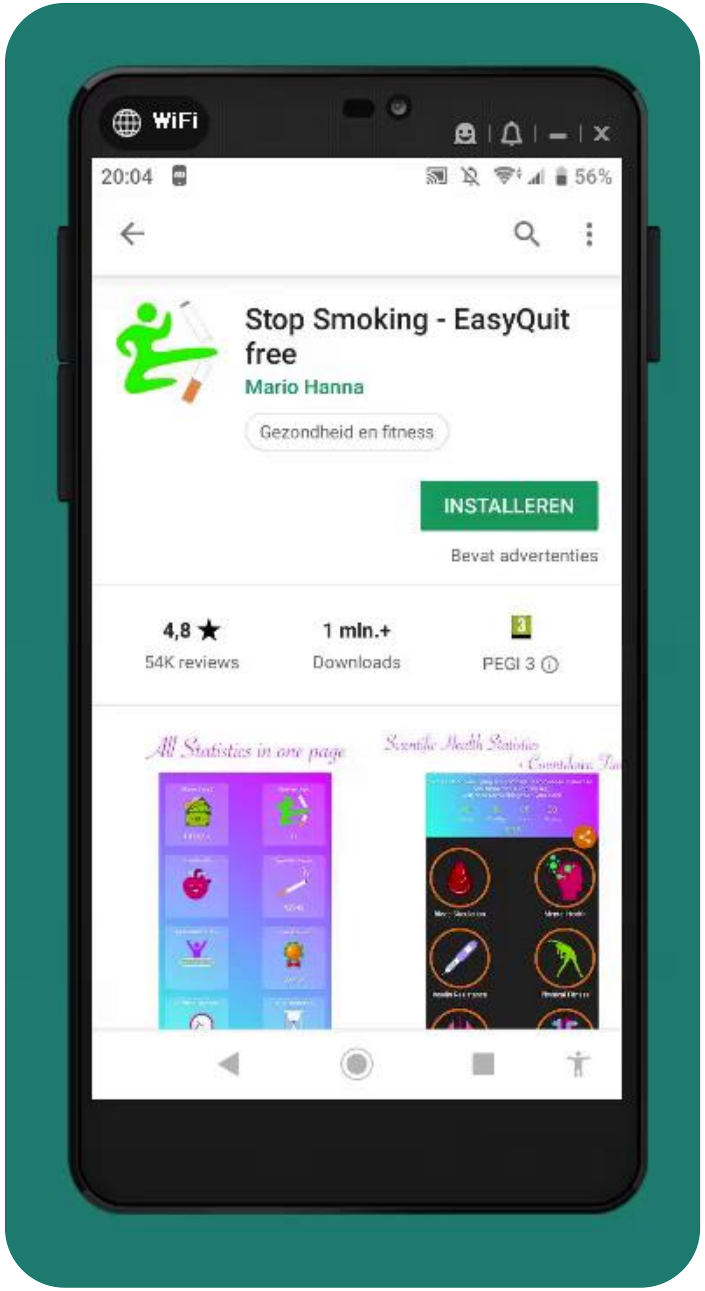

	Ziet/leest	Reageert	Information Cue
1		"Yes. It actually still doesn't say.... Oh wait, here it is."	Korte beschrijving

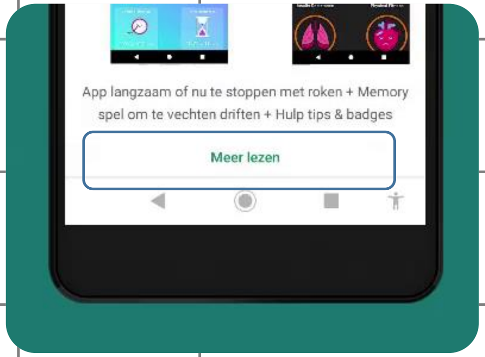

Functionaliteit App	Opmerking onderzoeker
–	The participant says: "It still doesn't say.... Oh wait, here it is" The conversation just before this one was about what he missed in terms of information and that he would like to see one sentence with each app in the search results overview with information about 'how they work'. It seems like he actually expects to see that sentence immediately when opening the app detail page, but doesn't get to see it. After "Oh wait, here it is" he clicks on Read more.

Doet	Reden
Clicks on 'Read more'	–

1.2 Description

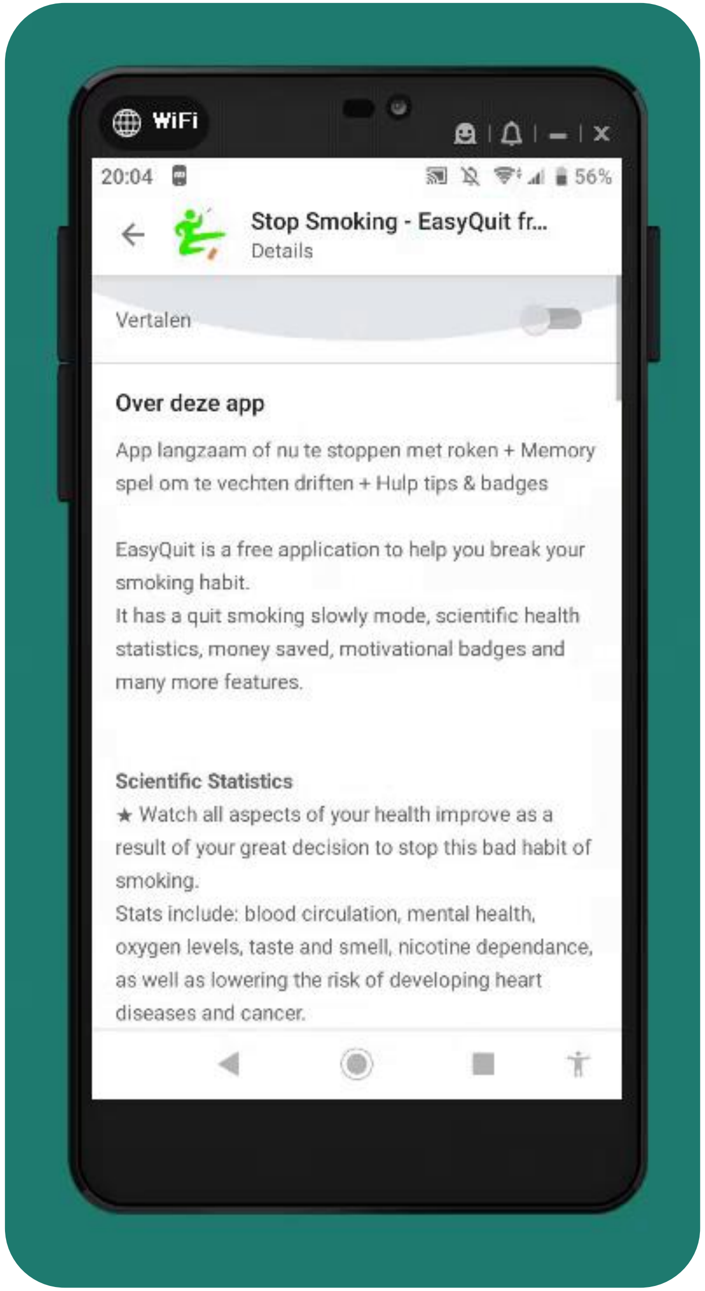

	Ziet/leest	Reageert	Information Cue
		R: Yeah, then I'm just going to read actually. [#00:31:11-0#]<br>I: What are you going to read then?<br>R: The little story that's out there about this app....	
		"wait, translating is easier too..."	

Doet	Reden
Clicks on slider ‘Translate’ [Vertalen]	–

Functionaliteit App	Opmerking onderzoeker
–	–

1.3 Description

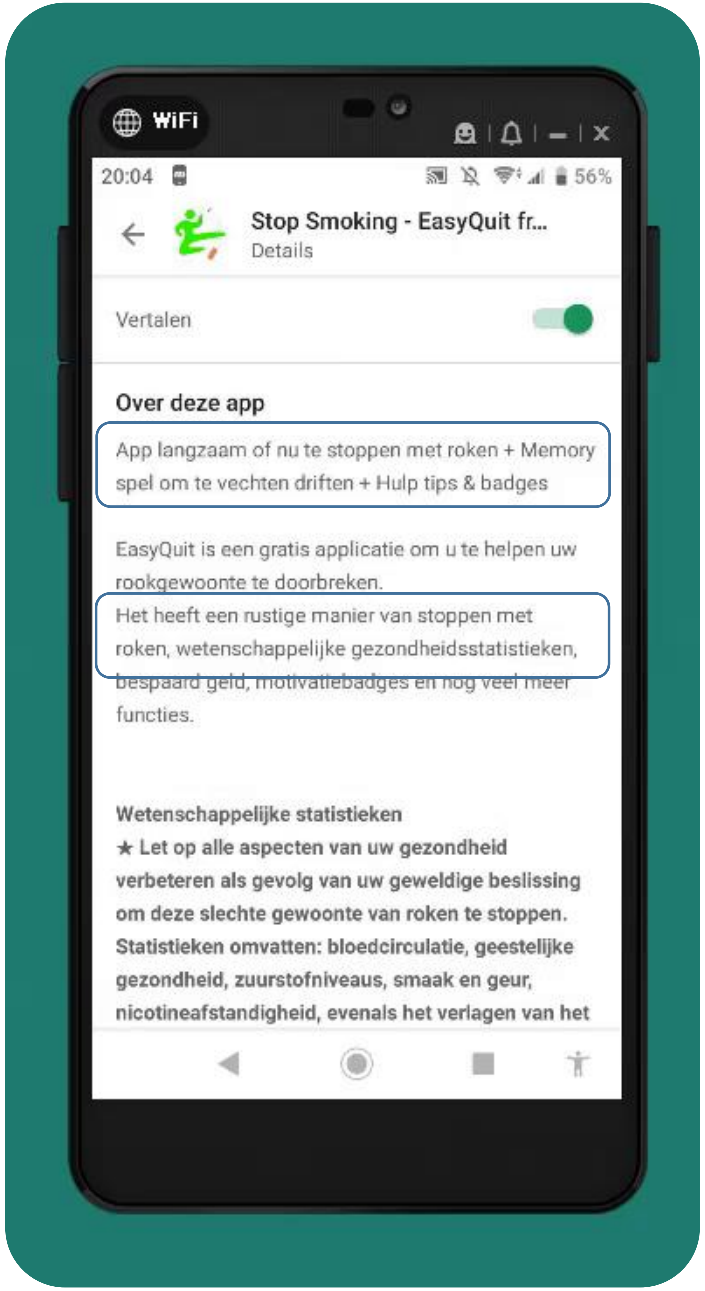

	Ziet/leest	Reageert	Information Cue
1	Memoryspel	"There's then games included on an app that you can then kill your time instead of lighting a cigarette [laughs]. I think, I think, "memory game."	Beschrijving
2	heeft een rustige manier van stoppen	"That appeals to me then, that you don't get dings all day long. They are pretty good at that, I think, apps. To ehh... those push up messages. That's of course the case with a lot of things. That I think 'yes, that's not very useful', it seems to me."	Beschrijving

Doet	Reden
–	–

Functionaliteit App	Opmerking onderzoeker
• Game<br>• 'Stop Smoking Slowly Mode”	At point 2. - The participant reads: 'quiet way to stop smoking' and interprets that as: 'they let you stop quietly'/ 'you don't get too many push messages'. What is meant is that there is a 'Stop Smoking Slowly Mode' in the app ("to help you stop smoking slowly"), but you don't see that here yet (see 1.7 for the text in the description about this).<br><br>See also 1.13 for related note on this.

1.4 Description

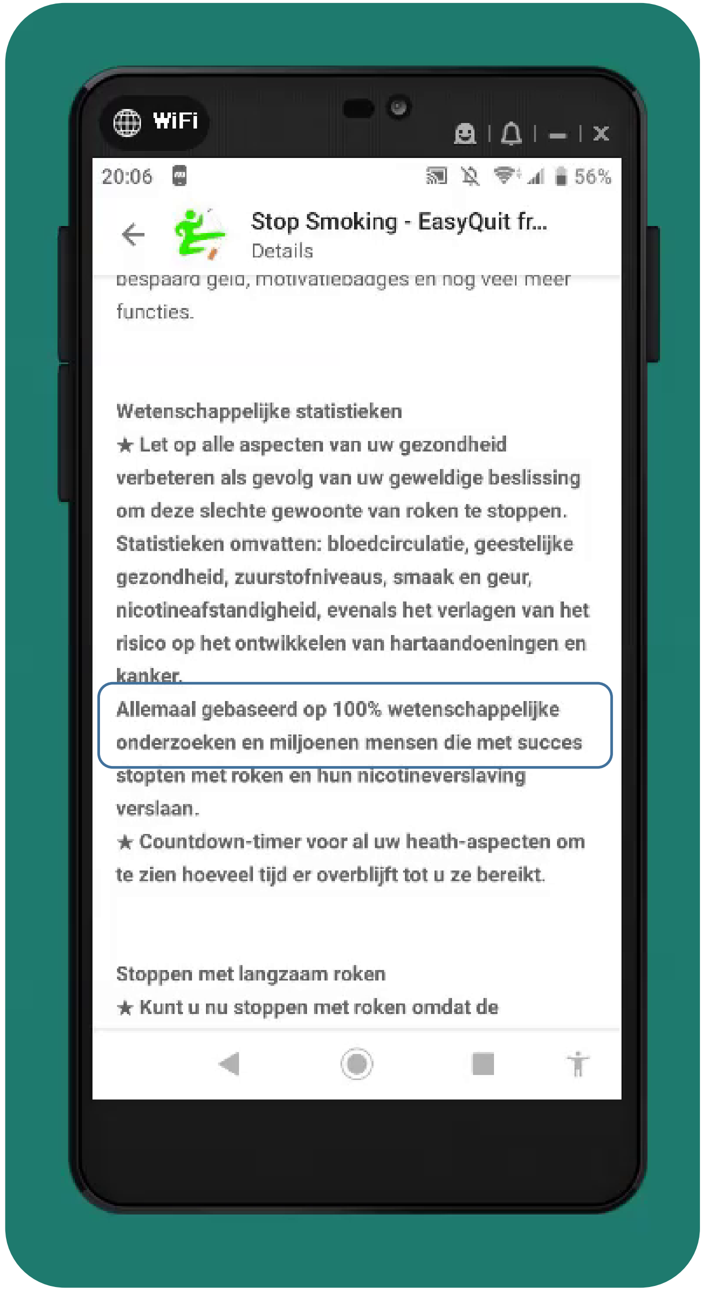

	Ziet/leest	Reageert	Information Cue
1	"On scientific studies"	"Here's some stuff: 'scientific' that they do have there, ehh..." "That's okay too, of course, I guess. I don't know if that's true then, but that, yes.... "	Beschrijving

Doet	Reden
–	–

Functionaliteit App	Opmerking onderzoeker
• Wetenschappelijke basis	–

1.5 Description

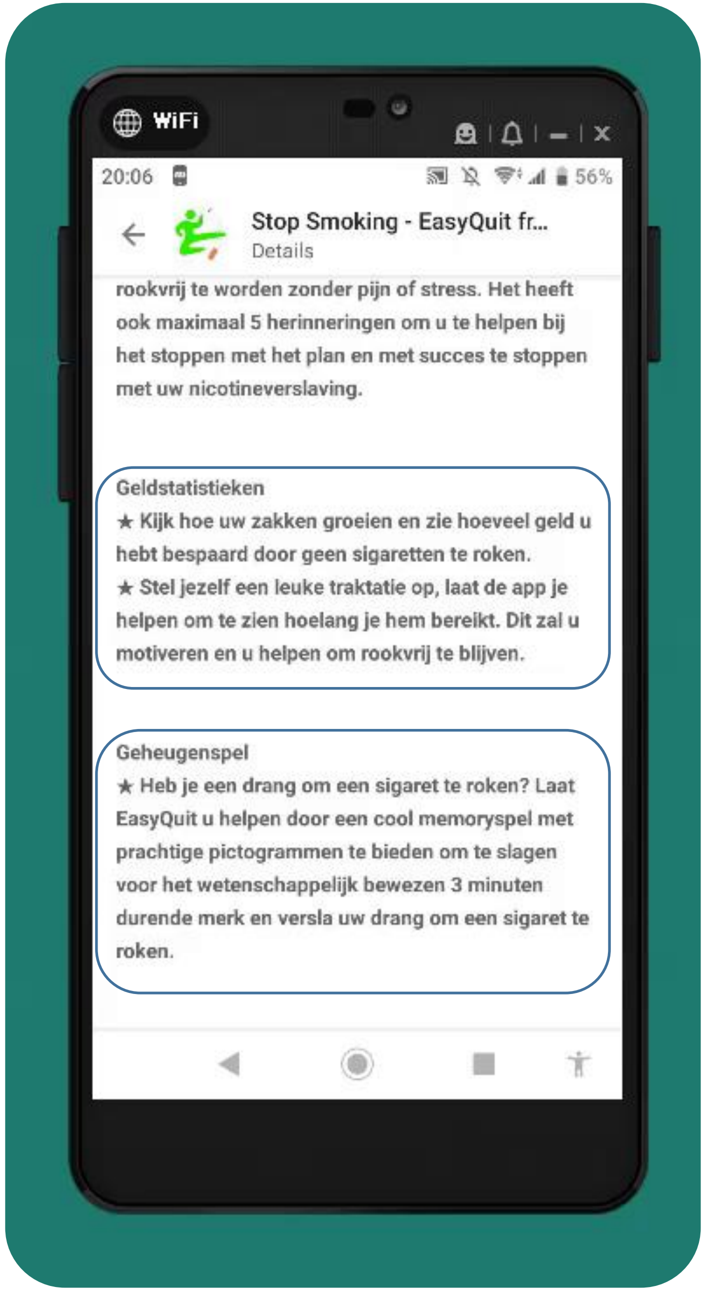

	Ziet/leest	Reageert	Information Cue
1	Geldstatistieken	"Ah, that's funny too of course: how much money you would save then if you did something ehh.... It also saves money if you stop smoking of course."	Beschrijving
2	Memoryspel	"This is kind of funny: memory game. For three minutes [laughs loudly]. That's nice, that in three minutes you can play a memory game where you normally could have smoked a cigarette."	Beschrijving
3	Memoryspel (beeldopname: vanaf 00:11:52)	[I: Yes, because for example that game, you call that funny. But do you mean... Do you also think it will help you or is it more just funny? [#00:36:37-5#]]<br><br>"Well, yeah maybe I think, if I do.... I often smoke three, four, yes, two, three, four cigarettes in the evening, but most of the time I'm just sitting on the couch watching television and I think "oh, I'll have another cigarette". Then you might still think of "I can play a game too." [Laughs] And then maybe that will motivate you. Of course, that could have a funny effect. But I actually had never thought about that myself, about something like that. That's what an app like that is for, of course, which you haven't thought about yourself, to be able to do that.... [deliver]"	Beschrijving
4	Geldstatistieken (beeldopname: vanaf 00:12:50)	[I: And with those money statistics, is that...? Do you want to just say from 'funny' to 'it helps me'?"]<br>"Well, money statistics, I don't know whether I find that very, yes, beautiful or that I would really start saving for it, or ehh. Because of course you hear that from many people who then put all that aside, such a pack of cigarettes, and then of course you can go on vacation at the end of the year. I think I'm too sober for that. That's six euros. Yes, if you count a pack of cigarettes every day, but yes, I smoke one pack of cigarettes during the week, so yes, the six euros is not so important, I think."	Beschrijving

Functionaliteit App	Opmerking onderzoeker
• Tellertjes geld<br>• Spel	–

1.6 Description

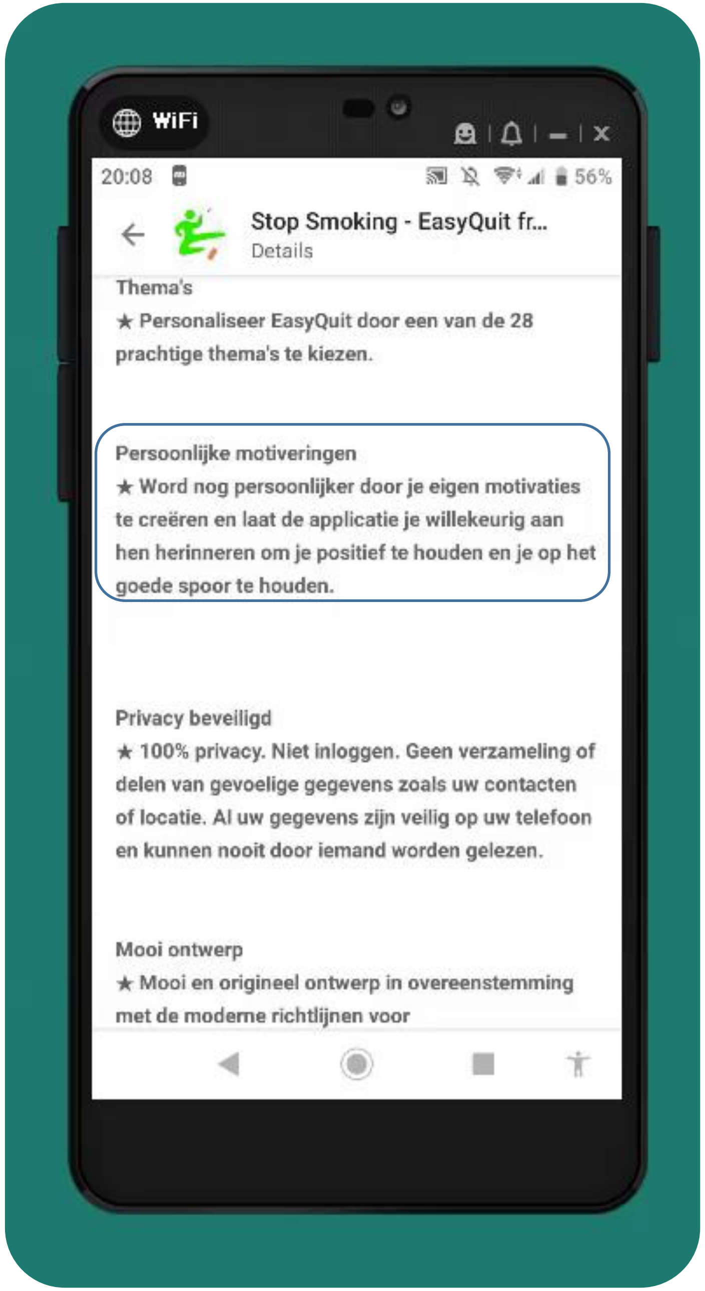

	Ziet/leest	Reageert	Information Cue
	Persoonlijke motiveringen	"You can, of course, also give your own personal motivations.... That would be, yes... I don't have anything to do with that further, but.... "	Beschrijving

Doet	Reden
–	–

Functionaliteit App	Opmerking onderzoeker
• Motiverende berichten (gepersonaliseerd)	–

1.7	Description

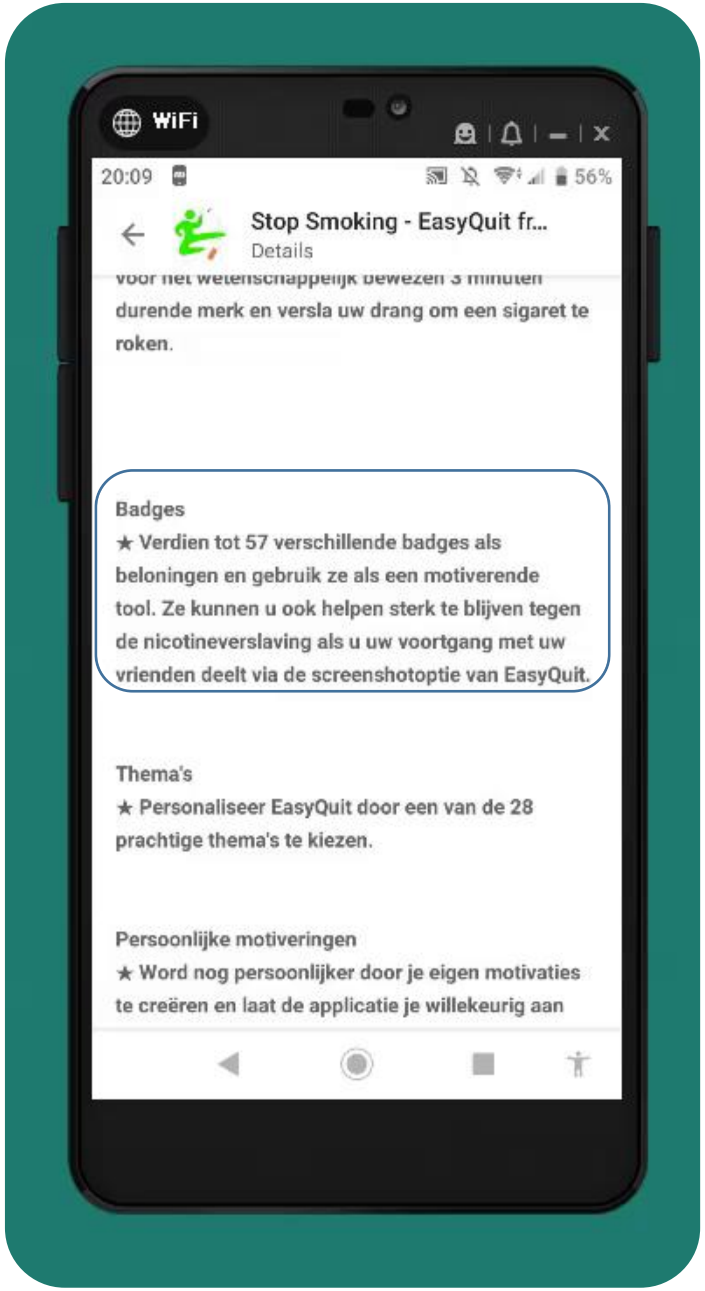

	Ziet/leest	Reageert	Information Cue
1	Badges	"Like badges, yeah... I think that's kind of funny, too, I guess. On the other hand, yeah, I don't know if I'd really be motivated by that. To get all the badges you can... I'm not sure about that. Maybe eventually, when you suddenly have twenty, you think "I want them all now". That could still happen of course."	Beschrijving

Functionaliteit App	Opmerking onderzoeker
• Badges	–

Doet	Reden
–	–

1.8 a Description

Reageert

Hm. [#00:34:46-7#]
I: Why did you say "hm"? [#00:34:48-1#]
R: No, yes, just, that 'he can actually choose a lot of things. [#00:34:57-3#]
I: Can you choose it? [#00:34:57-7#]
R: Well, you can, of course, choose your own personal motivations.... That would then, yeah. I don't have anything to do with that further, but... [#00:35:06-1#] (repeating 1.6)
I: Do you mean the app seems to have a lot of features? [#00:35:08-5#]
R: Yes, many functions. That's then of course... [#00:35:11-3#]
I: What is that? [#00:35:12-7#]
R: Yeah, kind of funny, I guess, then you have something to... That you still sometimes look into it and then you think, I'm going to, if you're bored, go through it all a bit and see what you can do with it. To explore what you... [#00:35:28-8#]
I: Yeah, so you can... Do you mean you can discover in the app what's nice for you? [#00:35:33-1#]
R: Yes. You don't have to use all the features, of course, but the fact that there's a lot of choice, of course, is...

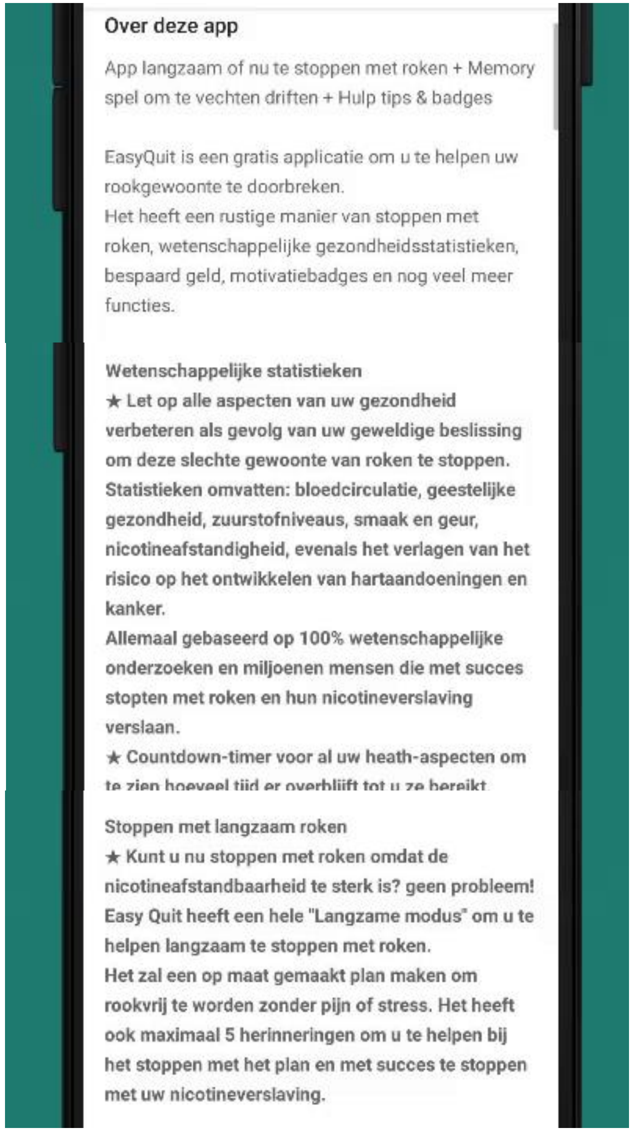

Over deze app

App langzaam of nu te stoppen met roken + Memory spel om te vechten driften + Hulp tips & badges

EasyQuit is een gratis applicatie om u te helpen uw rookgewonte te doorbreken.
Het heeft een rustige manier van stoppen met roken, wetenschappelijke gezondheidsstatistieken, bespaard geld, motivatiebadges en nog veel meer functies.

Wetenschappelijke statistieken
★ Let op alle aspecten van uw gezondheid verbeteren als gevolg van uw geweldige beslissing om deze slechte gewoonte van roken te stoppen. Statistieken omvatten: bloedcirculatie, geestelijke gezondheid, zuurstofniveaus, smaak en geur, nicotineafstandigheid, evenals het verlagen van het risico op het ontwikkelen van hartaandoeningen en kanker.
Allemaal gebaseerd op 100% wetenschappelijke onderzoeken en miljoenen mensen die met succes stopten met roken en hun nicotineverslaving verslaan.
★ Countdown-timer voor al uw heath-aspecten om te zien hoeveel tijd er overblijft tot u ze bereikt

Stoppen met langzaam roken
★ Kunt u nu stoppen met roken omdat de nicotineafstandbaarheid te sterk is? geen probleem! Easy Quit heeft een hele "Langzame modus" om u te helpen langzaam te stoppen met roken.
Het zal een op maat gemaakt plan maken om rookvrij te worden zonder pijn of stress. Het heeft ook maximaal 5 herinneringen om u te helpen bij het stoppen met het plan en met succes te stoppen met uw nicotineverslaving.

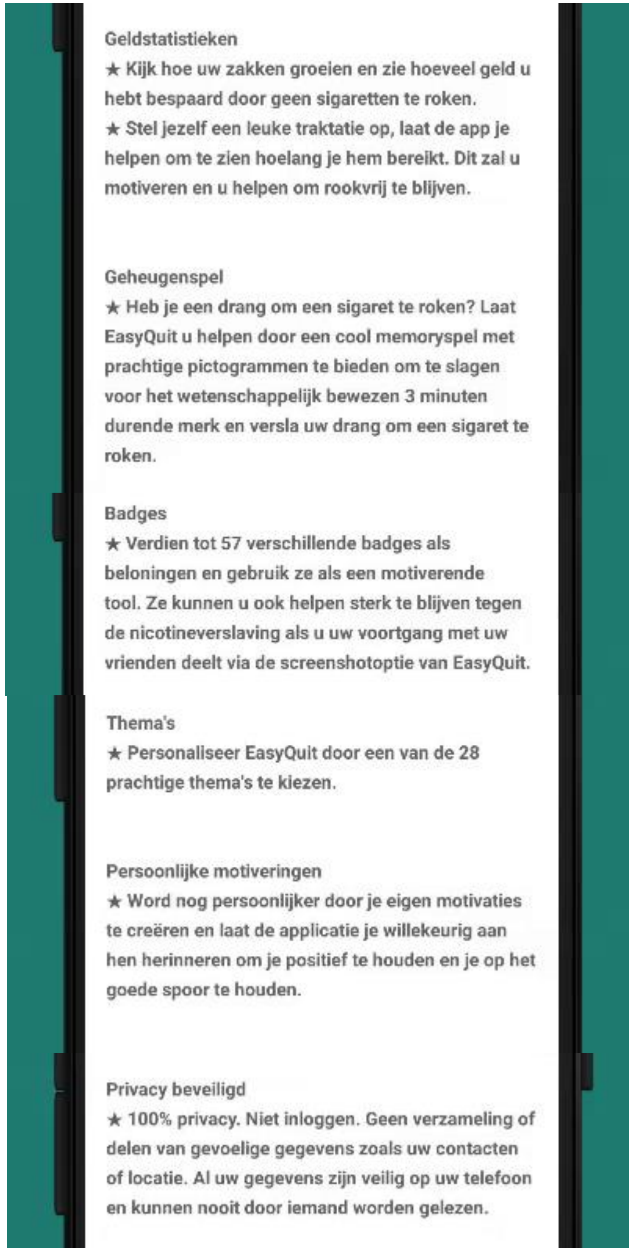

Geldstatistieken
★ Kijk hoe uw zakken groeien en zie hoeveel geld u hebt bespaard door geen sigaretten te roken.
★ Stel jezelf een leuke traktatie op, laat de app je helpen om te zien hoelang je hem bereikt. Dit zal u motiveren en u helpen om rookvrij te blijven.

Geheugenspel
★ Heb je een drang om een sigaret te roken? Laat EasyQuit u helpen door een cool memoryspel met prachtige pictogrammen te bieden om te slagen voor het wetenschappelijk bewezen 3 minuten durende merk en versla uw drang om een sigaret te roken.

Badges
★ Verdien tot 57 verschillende badges als beloningen en gebruik ze als een motiverende tool. Ze kunnen u ook helpen sterk te blijven tegen de nicotineverslaving als u uw voortgang met uw vrienden deelt via de screenshotoptie van EasyQuit.

Thema's
★ Personaliseer EasyQuit door een van de 28 prachtige thema's te kiezen.

Persoonlijke motiveringen
★ Word nog persoonlijker door je eigen motivaties te creëren en laat de applicatie je willekeurig aan hen herinneren om je positief te houden en je op het goede spoor te houden.

Privacy beveiligd
★ 100% privacy. Niet inloggen. Geen verzameling of delen van gevoelige gegevens zoals uw contacten of locatie. Al uw gegevens zijn veilig op uw telefoon en kunnen nooit door iemand worden gelezen.

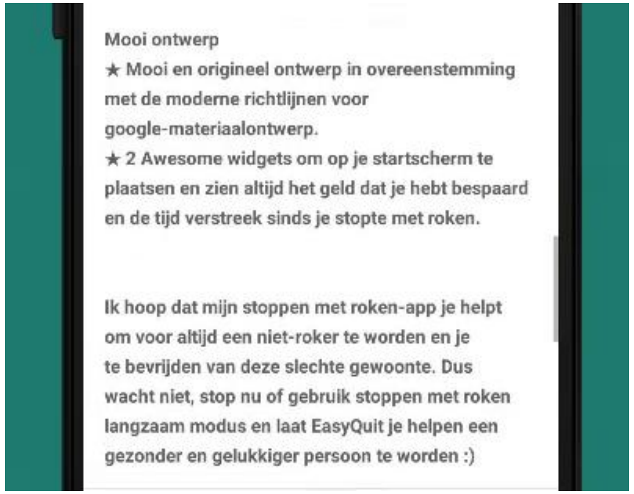

Mooi ontwerp
★ Mooi en origineel ontwerp in overeenstemming met de moderne richtlijnen voor google-materiaalontwerp.
★ 2 Awesome widgets om op je startscherm te plaatsen en zien altijd het geld dat je hebt bespaard en de tijd verstreek sinds je stopte met roken.

Ik hoop dat mijn stoppen met roken-app je helpt om voor altijd een niet-roker te worden en je te bevrijden van deze slechte gewoonte. Dus wacht niet, stop nu of gebruik stoppen met roken langzaam modus en laat EasyQuit je helpen een gezonder en gelukkiger persoon te worden :)

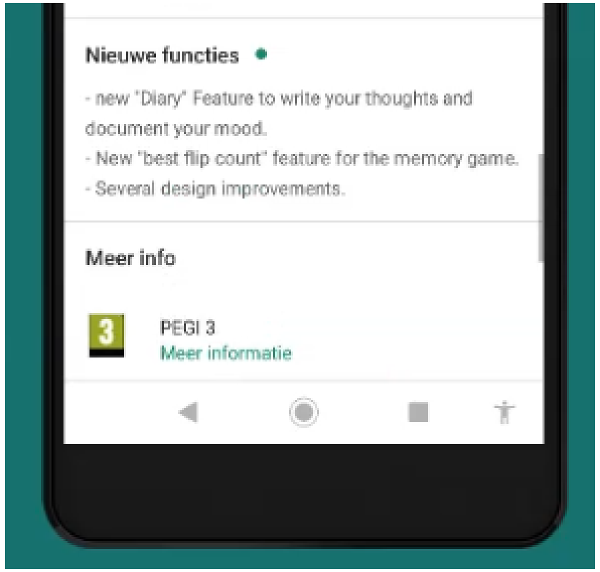

Nieuwe functies

- new "Diary" Feature to write your thoughts and document your mood.
- New "best flip count" feature for the memory game.
- Several design improvements.

Meer info

PEGI 3
Meer informatie

1.8 b | Description

Opmerking onderzoeker

The images show what the participant is NOT naming while reading (all unshaded areas of the screen). Should we do anything with that?

Doet	Reden
Returns to app detail page and scrolls to reviews	Yes, I'll have to check out the reviews then of course. [#00:38:41-3#]<br>I: Yeah, for me it doesn't have to be, but.... [#00:38:42-5#]<br>R: No, but I always do that myself then. [silence] [#00:38:50-3#]

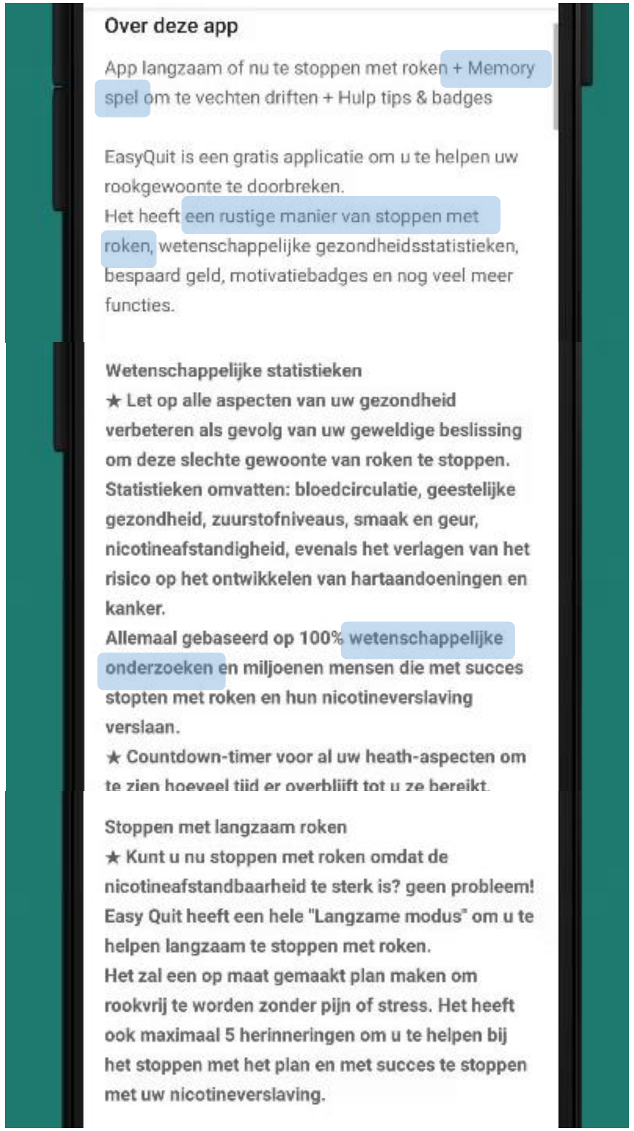

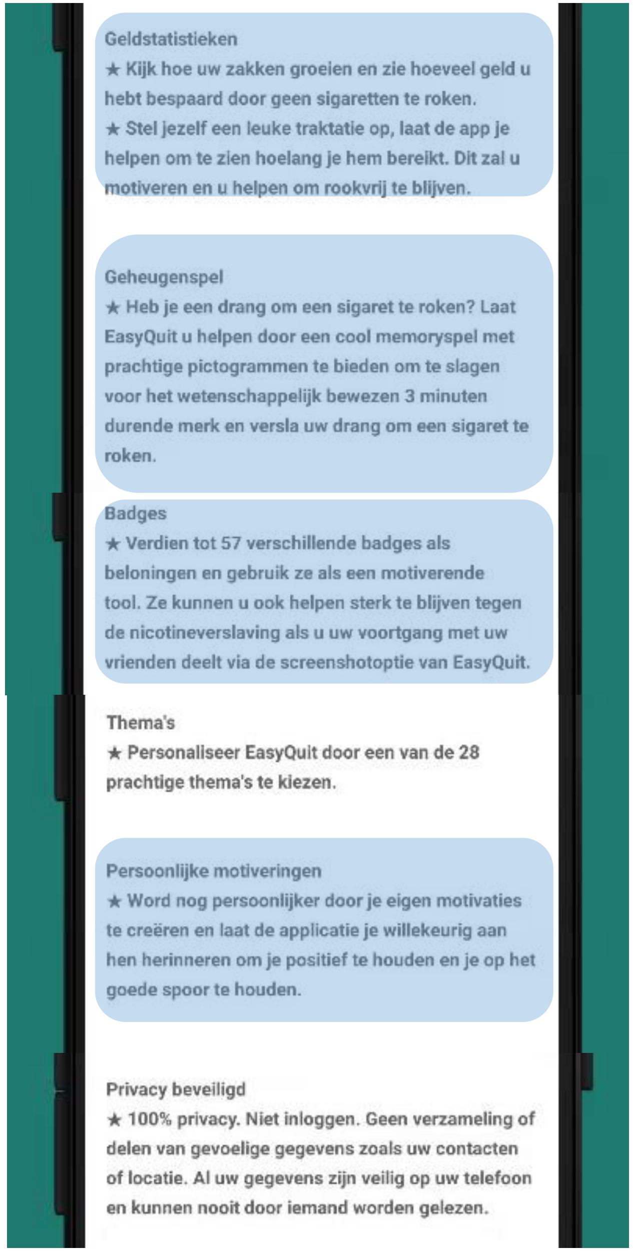

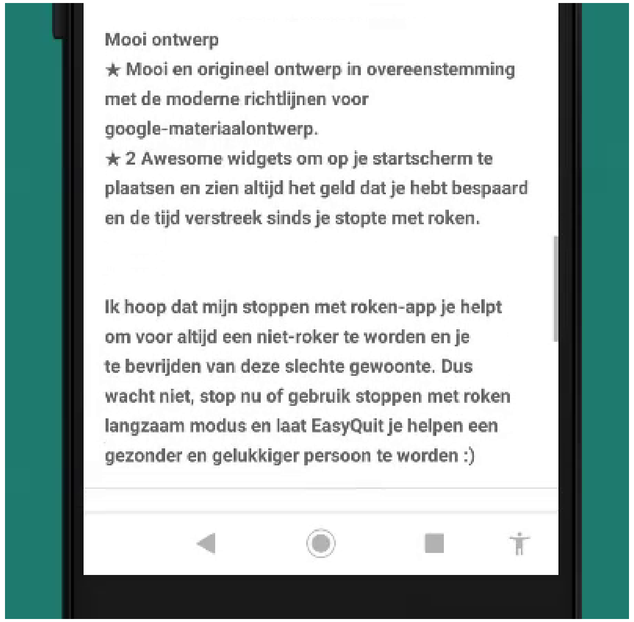

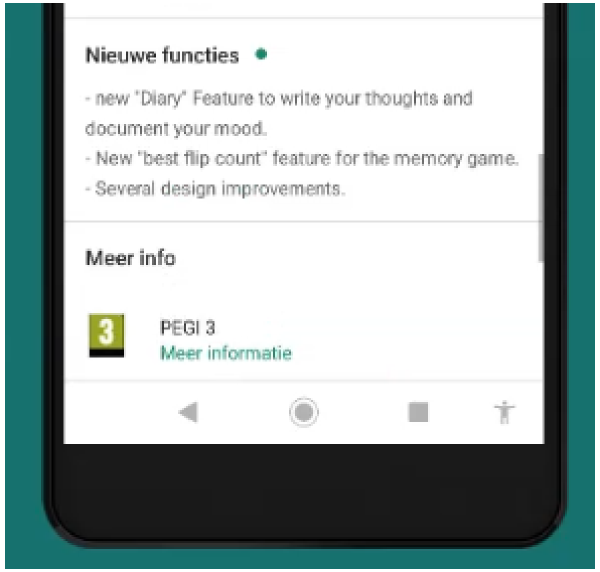

1.9 Detailed app info screen

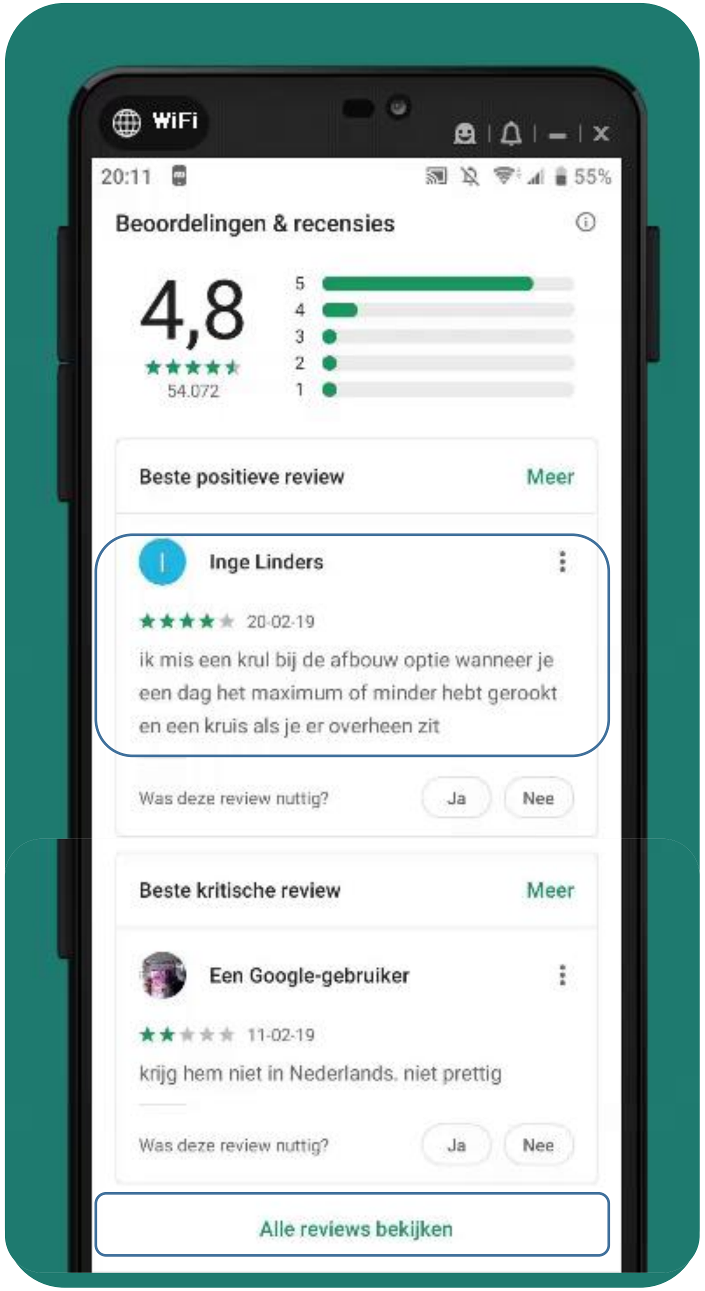

	Ziet/leest	Reageert	Information Cue
1	Inge Linders	"Oh yeah, well 'Inge Linders', but then I still don't really understand that because that's about the app itself."	Reviews (op app-detailpagina > ‘Beste positieve review’ & ‘Beste kritische review’)

Doet	Reden
Clicks on 'View all reviews'	

Opmerking onderzoeker
**About reviews in the Google Play Store**<br>• I noticed later (after the interview) that in the online Google Play Store (link) other reviews appear than the one the participant has on his screen. At least: Inge Linders is not among them on my screen (sorted by date and at February 20, 2019, there is no review by 'Inge Linders', nor is there any 'JS by Ake' among them).<br>• I wondered if that had to do with the fact that the participant had the page translated, but it turns out not to be the case because on my smartphone I do get to see 'Inge Linders' and 'JS van Ake' in the Google Play Store. Incidentally, the reviews displayed on the screen of the participant, and the screen of my smartphone, differ, but that may be because reviews have been added in the meantime.<br>• This also makes me wonder again what/who determines what 'Most Relevant' means - you can sort the reviews on that. Upvotes (doesn't seem to be the case: I checked one review as useful, but that review did not move up in the ranking).

1.10 Reviews

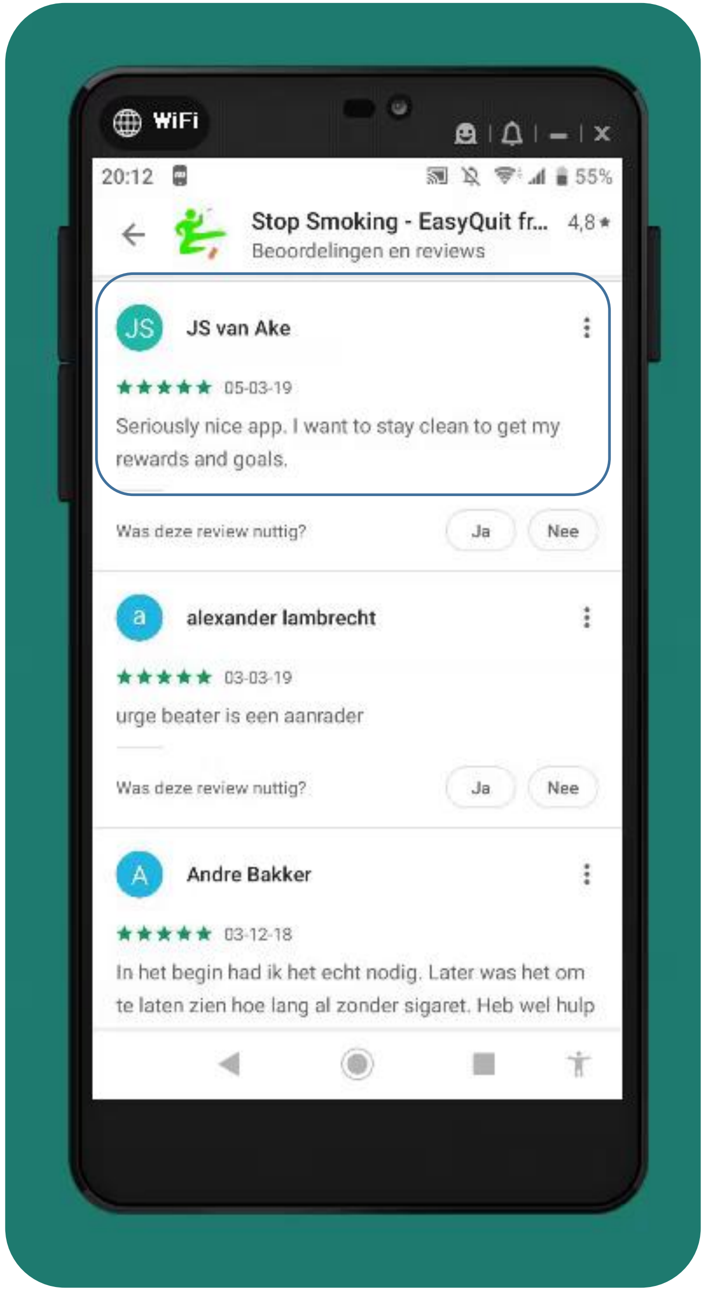

	Ziet/leest	Reageert	Information Cue
1	JS van Ake	"Yeah, now there's some stuff like that.... Here's just a good review though"	Reviews (Alles – gesorteerd op 'Meest relevant')
2	"I want to stay clean to get my rewards and goals"	Of course, if you get badges, you might think "I want them all". Maybe they have thought of a good way to motivate you.	Reviews

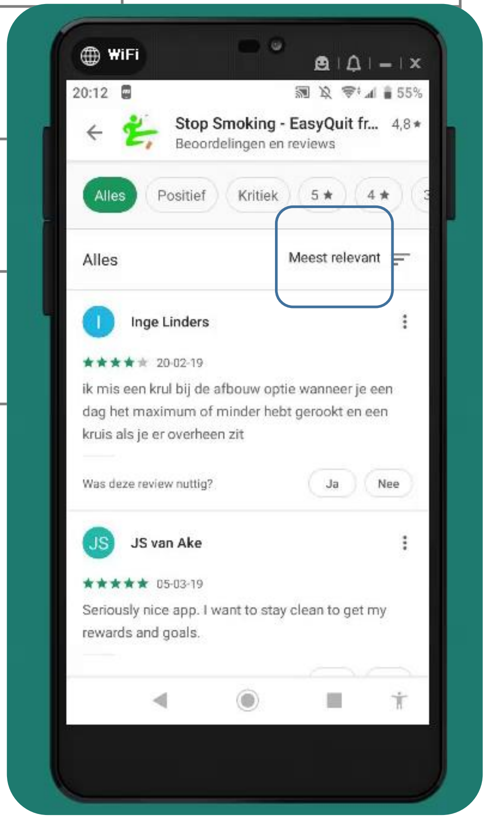

Functionaliteit App

- Badges

Opmerking onderzoeker

On point 1. - I'm curious what this participant thinks is "good" about this review - or more generally, when he thinks a review is good. Does he tell more about that later? I remember him saying later that in reviews he mainly wants to see confirmation of features of the app. Not 'this app was very nice for me' - that doesn't help him, but reflection on the functionalities of such an app. That would explain why he finds this review 'good'.

1.11 Reviews

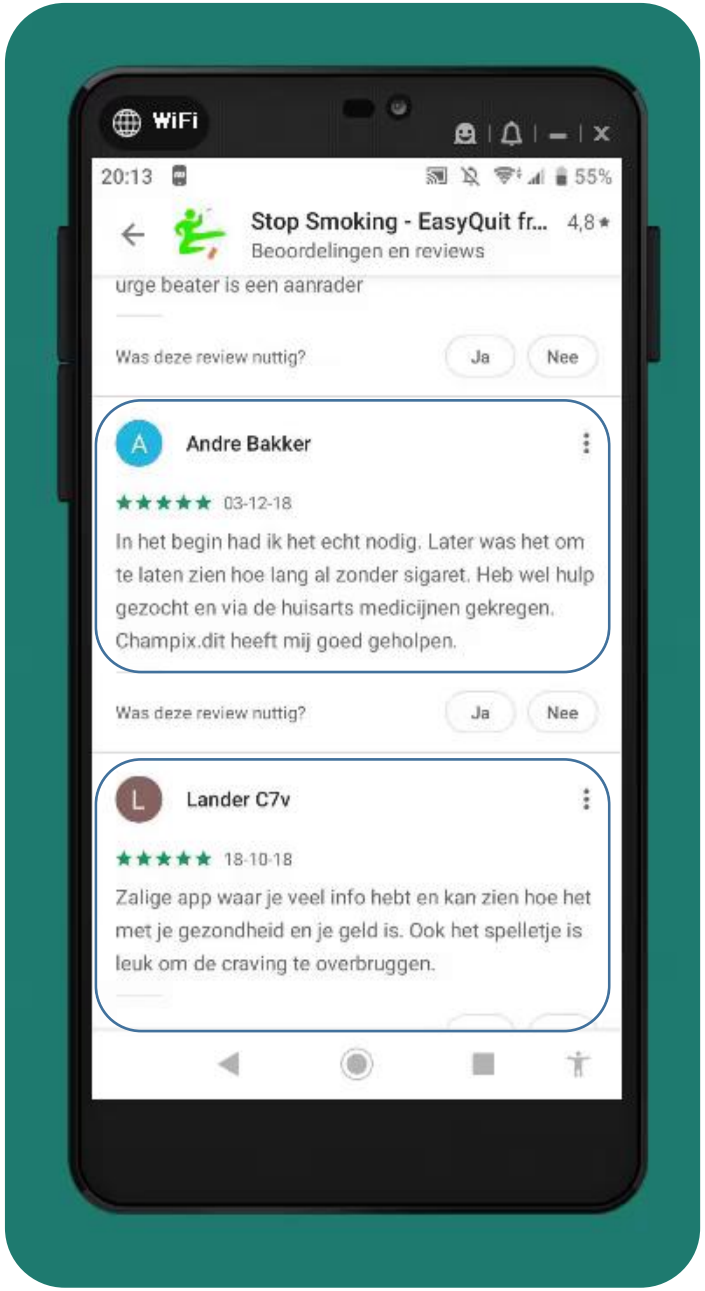

	Ziet/leest	Reageert	Information Cue
1	"In the beginning, I really needed it. Later it was to show how long I had been without a cigarette"	"Yeah, that's maybe what I think too, yeah.... "	Reviews
2	"Huisarts"	"That's probably a pretty bad smoker, this one, because he's been to the doctor. "	Reviews
3	"Lander" "kan zien hoe het met je gezondheid is"	"That's also nice to see, I think, what you might then... I don't know how that would be formulated, of course, but that seems interesting to me. "	Reviews

Functionaliteit App
• Gezondheidsinformatie

Opmerking onderzoeker
At point 1. - The participant says: "Here it also says, "In the beginning I really needed it. Etc." What does he mean by that? It does not go on to the badges he was talking about just before that (see 1.10). Is what he reads ("at first I really needed it, later it was to show how long I had been without a cigarette") a confirmation of something he said earlier? I can't really relate it to any previous comment.... It seems that he has thought something about the use of apps (that you will need it especially in the beginning, and later you can use it especially for the counters?) but I don't think he said that out loud anywhere.
On point 2. - The review of "Andre Bakker" is written by someone who indicates that he was medicated through his family doctor, and the participant makes a comment about that. It could be that this makes the participant value the review less, because he considers himself a light smoker (and therefore different). But that is pure assumption. Should we do anything with that? Can you do anything with it at all?
Participant mainly reads descriptions (as a source of information). But, he also gets input from reviews:<br>1. For ideas about useful features (it seems that he gets an idea here, by reading a review, about something that seems nice to him to have in an app (something that an app does).<br>2. Adjusts perception about (motivating) features , e.g. about badges (see 1.10, point 2)<br>3. He discovers features of the app that he did not know from the description.

1.12 Reviews

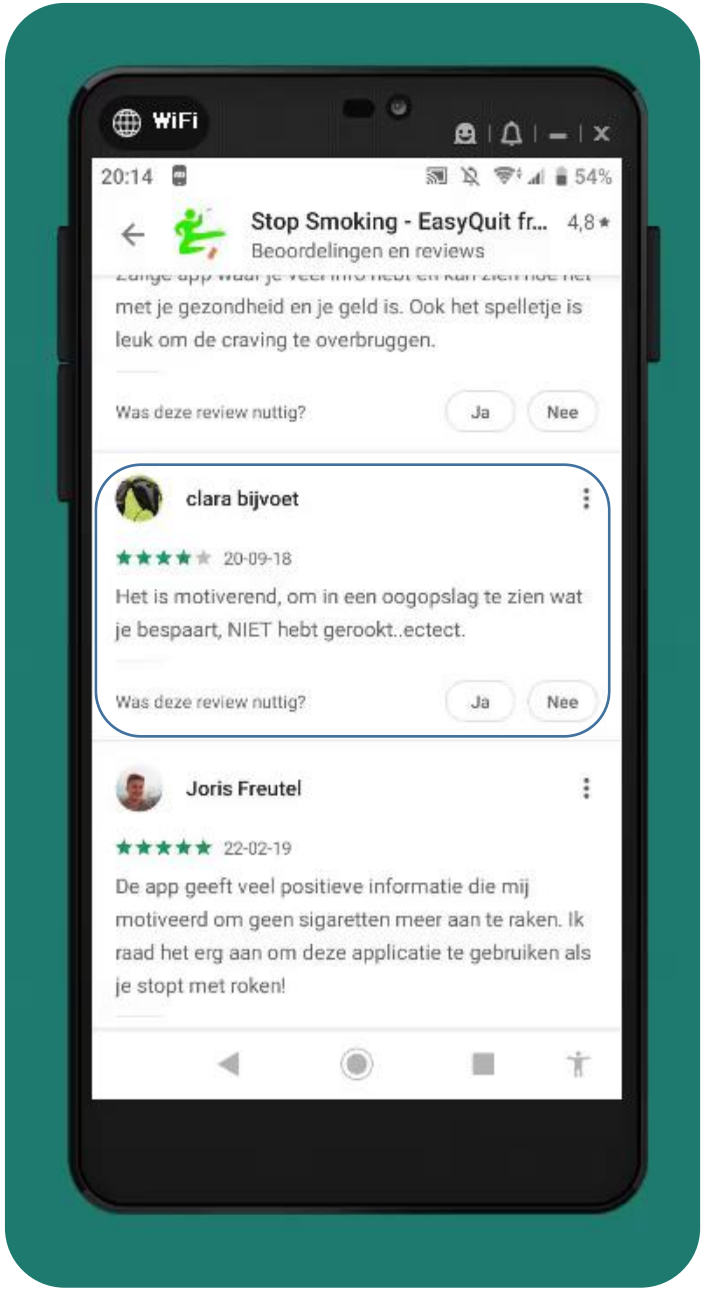

	Ziet/leest	Reageert	Information Cue
1	"het is in één opslag te zien wat je bespaart, NIET hebt gerookt, et cetera"	"That's easy too, not that you're everywhere yet.... That you can look at it, I think... Yes, that you don't have to scroll through everything all the time. "	Reviews
2			
3			

Doet	Reden
–	–

Functionaliteit App	Opmerking onderzoeker
• Usability	–

1.13

Reviews

Dwelled on the screen, but said nothing about it.

Reageert

I: Are you looking for a particular thing to read right now, or is it more that you just... [#00:40:26-3#]
R: No, yeah, with the reviews I always just read a whole bunch, because otherwise.... You can go looking for something, but there's always something there that you think of.... I think it's more fun then to read everything and come up with a summary of that, of how it's then ehh.... [#00:40:44-5#]
I: Okay, well, don't let me keep you. Sorry to interrupt you.
[#00:40:47-7#] R: [laughs] Not that I'm going to read them all, but I always try to get through a few. Um. That's kind of nice.

[...]

He does get good reviews all the time, so that's, yeah.... [silence] Hm. Yeah. [#00:42:21-6#]

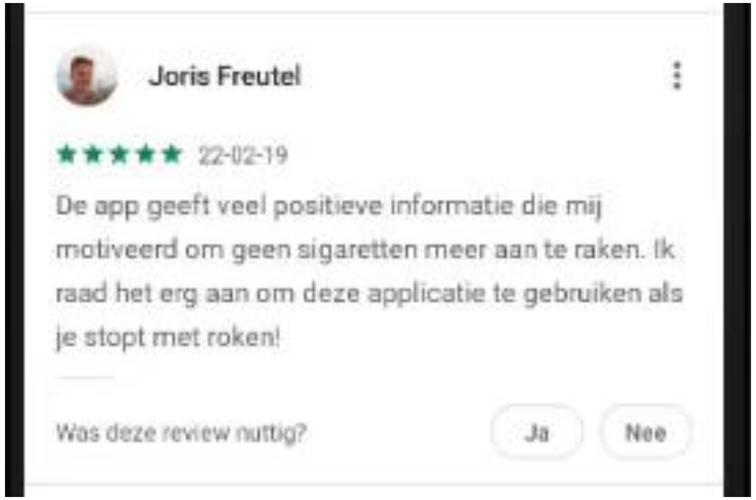

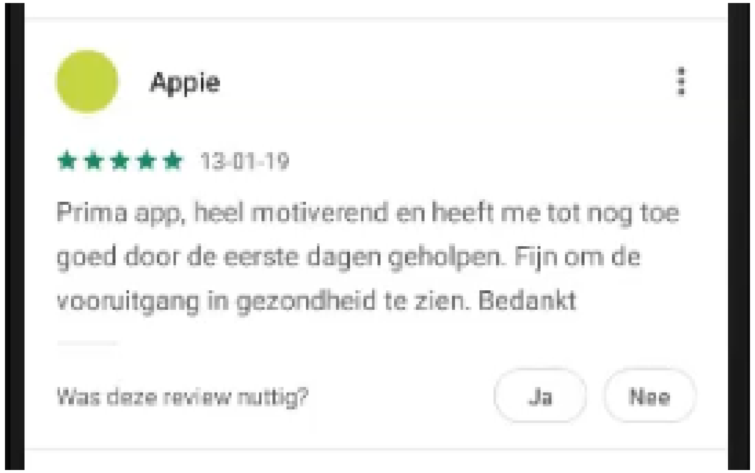

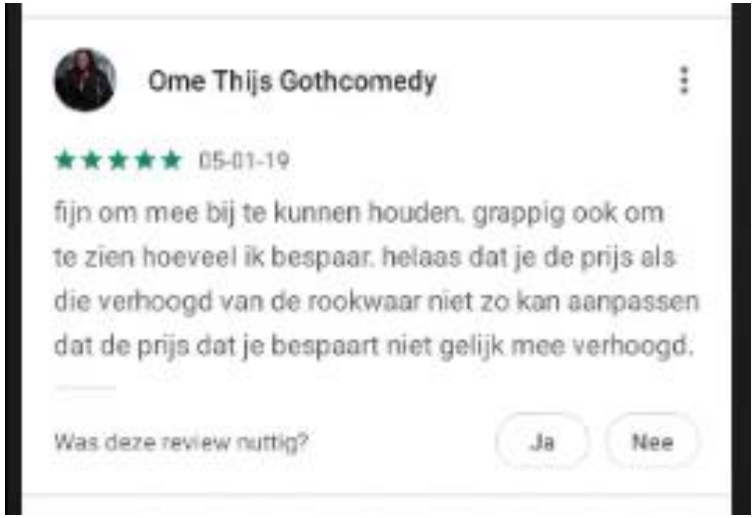

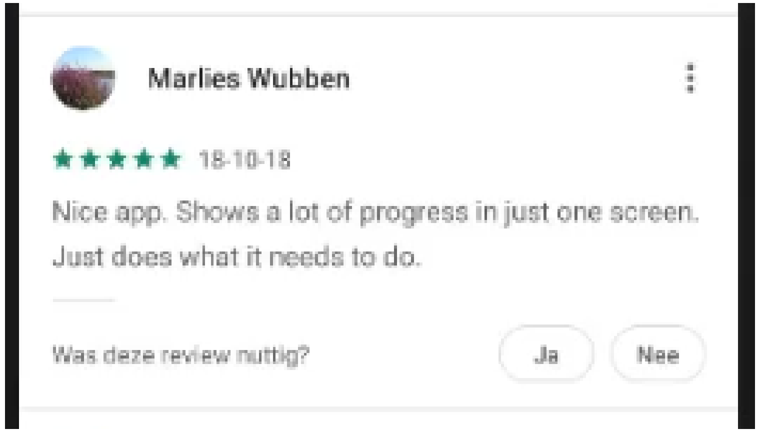

1.14 App #1 – EasyQuit free

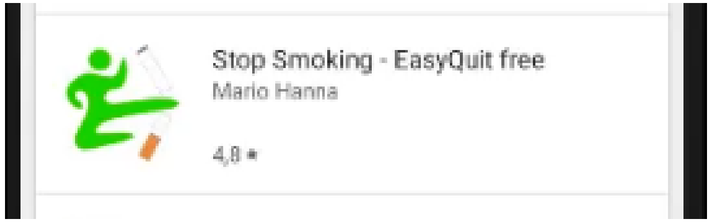

Reageert
Yes [#00:42:21-6#].<br>I: You know enough? [#00:42:23-0#]<br>R: I think so, yes. [#00:42:24-3#]<br>I: What do you know now? [#00:42:25-6#]<br><br>R: Well, this is well.... ehm, yes. Well, that I think it's easy to use, and it says something about science, how it works. And about your health, and yes, that it's just an interesting thing, I think. Not too motivating, or not that you're then pushed all day long to keep stopping, or.... [#00:43:05-3#]<br><br>I: That's something you don't want? [#00:43:06-4#]<br><br>R: No, I think that would drive me crazy. I think I would put it away, or throw it off faster. Then I think "yes, I'll do it myself". Then I always have the feeling that I'd rather do it myself than be pushed all the time. Then I get a little cocky and then I think....

Functionaliteit App	Opmerking onderzoeker
• Usability<br>• Wetenschappelijke basis<br>• Gezondheidsinformatie<br>• Notificaties (push berichten) >	With regard to the participant's remark about notifications: it is not entirely clear to me on what basis this participant got the impression that this app 'is not too motivating', that you 'are not pushed all day long'.<br>Perhaps because of comment 1.3 - 2: Quiet way of stopping.<br>If so, then his perception of the app was formed by a wrong assumption. He has assumed that 'quiet way of quitting' means: you are not pushed. But the description says that it is about help with 'slow quitting', for people who do not want to stop at once, but want to cut down. (see 1.7)
	He does this summary of his impression of the app from memory - that is, not from information on the screen. Does that mean these are the things that impressed the participant the most? Are these the most important features for him? When reading information from subsequent apps, does he look for info on the things he mentions here (ease of use, science, health, no push messages/not too motivating)?

Doet	Reden
Gaat terug naar het zoekresultatenoverzicht	"Oh yes, actually I had already read, of, yes.... Then I think, well I'd like to go and look at some more of course, what else is there between them of course. Because I don't know what, yes... I had clicked on one because I saw a funny little person there. It's a bit weird to think "we're going to do that!" as soon as you make your first choice. [laughs loudly]"
Scrolt naar beneden tot app #15	

3 List of search results

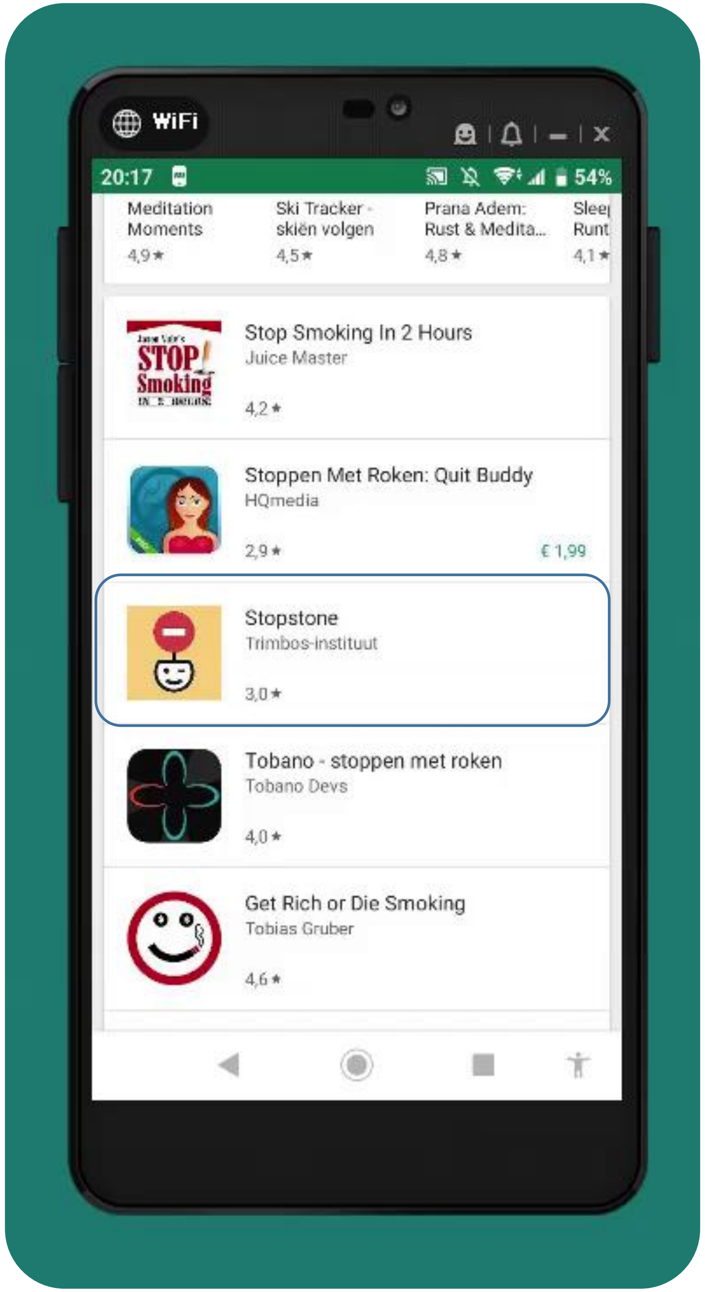

	Ziet/leest	Reageert	Information Cue
1	Trimbos	"Because I see here, I always find that, from the Trimbos Institute, then I think 'they must also know something about something',..."	Naam ontwikkelaar
2	3 sterren	"But then it only gets three stars. Then I always want to see why... How that came about. "	Rating

Functionaliteit App	Opmerking onderzoeker
• ...	–

Doet	Reden
• Klikt op 13de app in de lijst (Stopstone, Trimbos, #2)	"Am I curious then again. An institute like that should actually be reliable, that they make something working."

2.1 Description

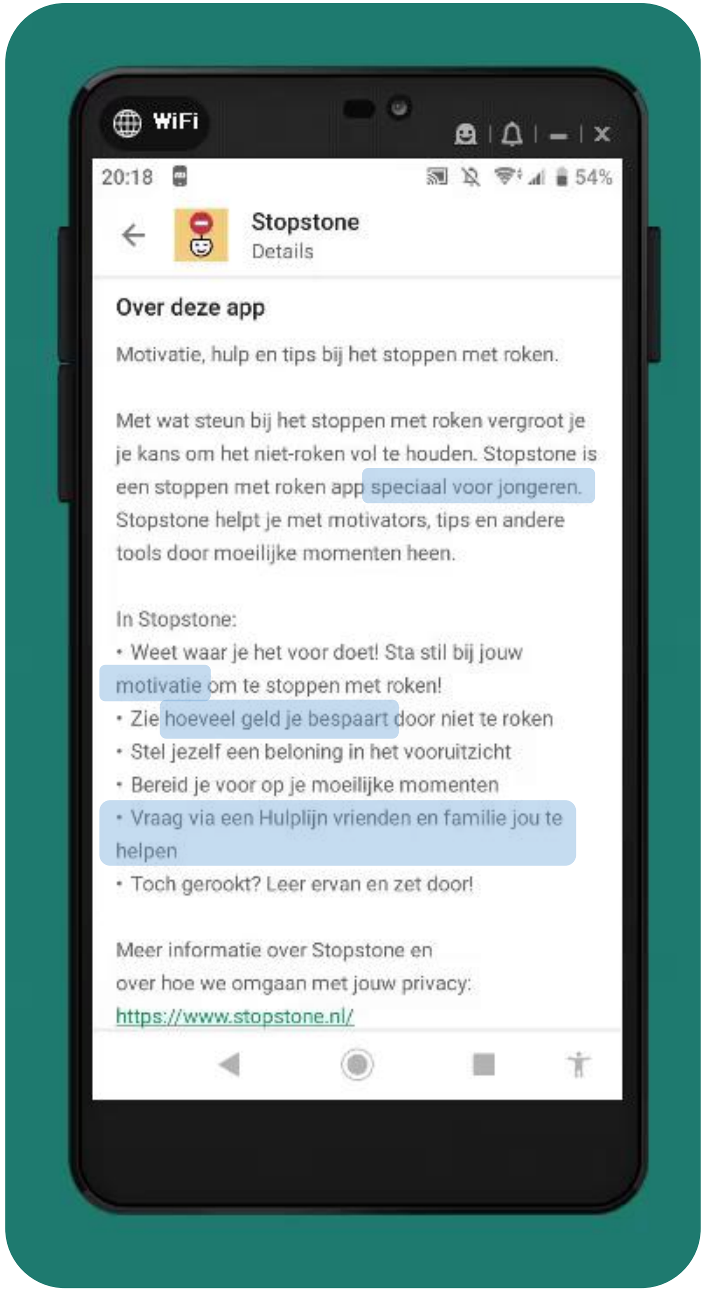

	Ziet/leest	Reageert	Information Cue
1	Motivatie	[mompel] "Motivatie" [stilte]	Beschrijving
2	Speciaal voor jongeren	"This, then again, is specifically for young people, it says. So that... [#00:45:34-4#]<br>I: Oh yeah. I don't know where you're putting yourself... [#00:45:37-1#]<br>R: No [laughs]. Yeah, not there. I'm 36 anyway, so then I should be eh.... "	Beschrijving
3	Hoeveel geld je bespaart	"Yes, of course they have everywhere from how much money you save. I get that too of course. Hm."	Beschrijving
4	"Vraag via een Hulplijn vrienden en familie jou te helpen"	"[...] then I think, yeah, I'm not really interested in that or anything. That eh... I just want to do that myself, not that I have to use helplines or other things for that, I think. So then I actually read it like that right away too... "	Beschrijving
5	motivatie	"It actually says the same thing as the other one, but more for young people. Also motivation, you should probably add that yourself then."	Beschrijving

Functionaliteit App	Opmerking onderzoeker
• Geldteller<br>• Hulplijn (vrienden en familie)<br>• Motivatie vastleggen	–

Doet	Reden
• Scrolt naar Ratings & reviews	–

2.2 Detailed app info screen

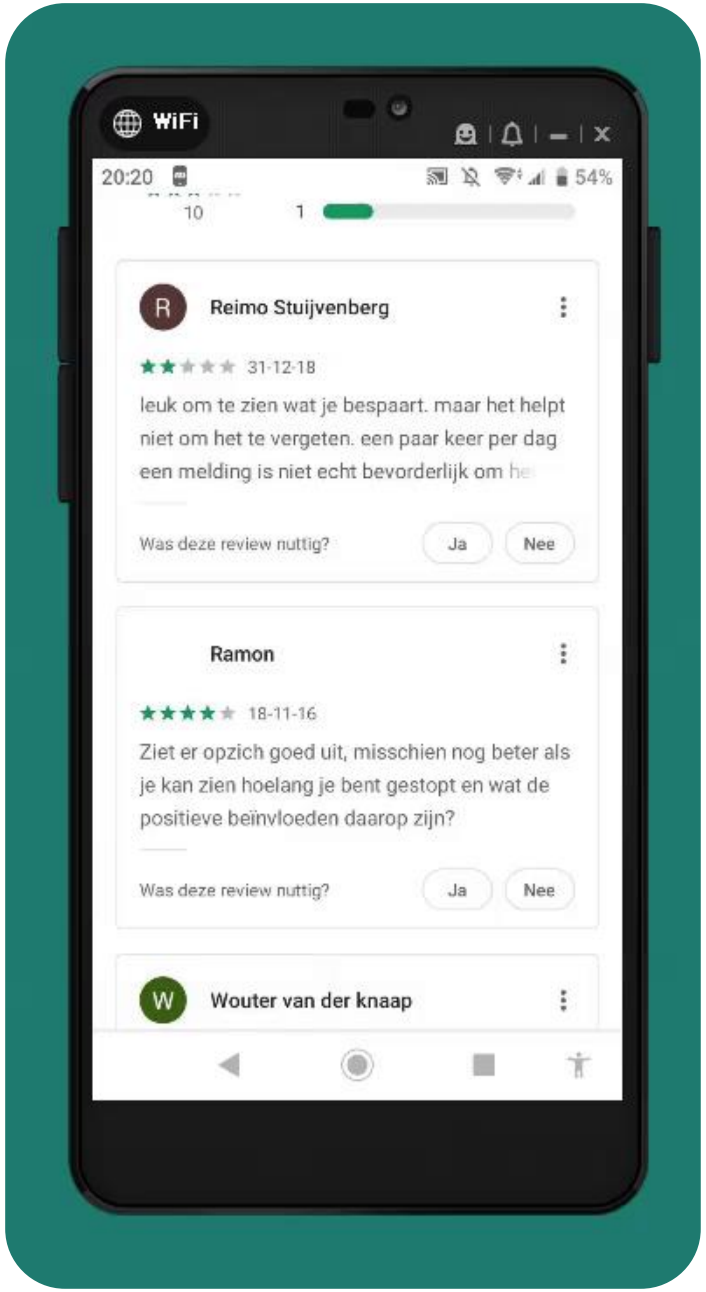

	Ziet/leest	Reageert	Information Cue
1	"misschien nog beter als je kan zien hoelang je bent gestopt en wat de positieve beïnvloeden daarop zijn"	"That's obviously nice to read if there's then something of, yes, I think then, some information of if you've stopped for so long what that's like for your lungs and for your body, for your health actually."<br><br>I: What you just actually mentioned with that other app as well. [#00:47:20-7#]<br>R: Yeah, that was even more of a part of it then, I think, that it was there ehh....	Review (op app-detailpagina)
3			

Functionaliteit App	Opmerking onderzoeker
• Gezondheidsinformatie	–

Doet	Reden
–	–

2.3 App #2 – StopStone

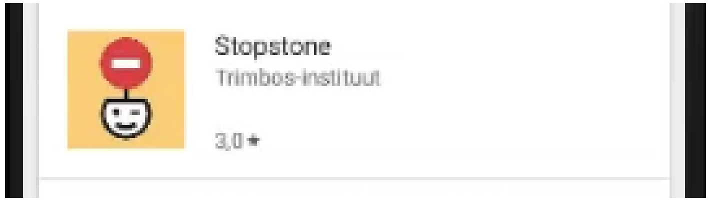

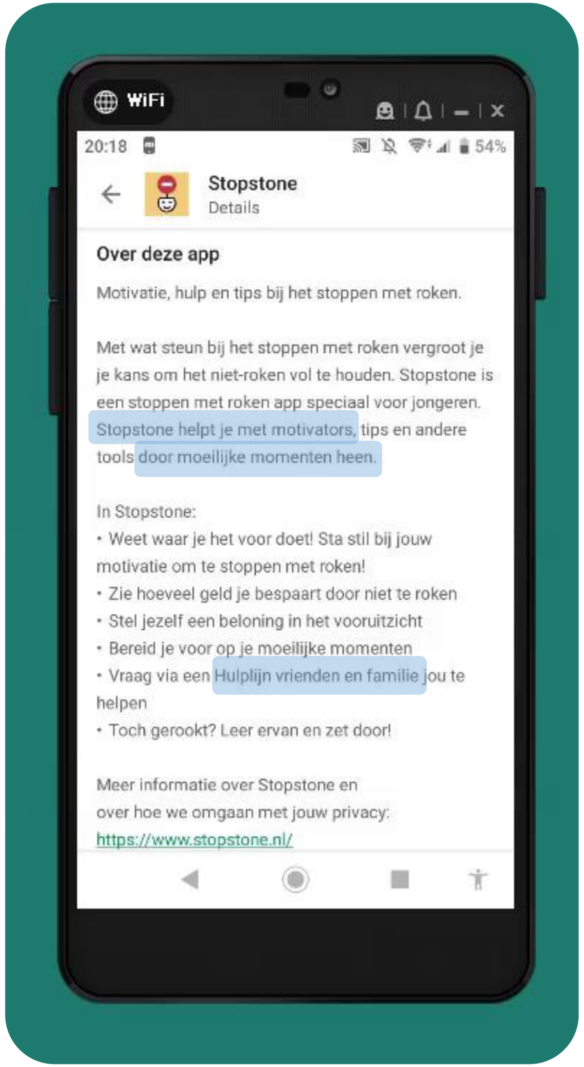

Reageert
I: Now what is the feeling you have left from this app? [#00:47:28-6#]<br>R: Yeah, yeah not really that I think of, I'd really want to use that, or something. You can see that right then, of "oh yeah". [#00:47:38-7#]<br>I: Yeah. And why not? [#00:47:43-4#]<br>R: Yes... Yeah, I think also with that kind of motivation, you have to write.... I'll take a look at what else was included that I then thought 'I don't really want to do that'. With such a helpline, friends and family, that's more really such a... Yeah. [silence] Yes, that's what I think... It also says here "Stopstone helps you with motivators". Yes, "through difficult moments". So I don't know if I should, yes.... I find that a bit too ehh, too sweet or something. That I think, yeah, [laughs]. There might be people who like that but, or like it, but I can't stand that very well then. [#00:48:28-4#]<br>I: Is it also kind of the style of how this is written down? [#00:48:29-4#]<br>R: Yes! Maybe it is indeed. That the text is like um, you're a victim, or a, um, yeah. Something like that I think. Yes, you're not, because you started it yourself anyway. So you also have to stop it yourself. On the one hand then I don't think you should then go, ehh. Well for me there is not the feeling then. [#00:48:55-8#]

Functionaliteit App	Opmerking onderzoeker
• Motivatie vastleggen<br>• Hulplijn 'vrienden en familie'<br>• Motivators	The participant says: "That the text so of eh, you are a victim [...]". I find it remarkable that he interprets the text in this way, because when I read the same text, I don't take it at all as if it implies that you are a victim.<br><br>The text in the app store: "With some support in quitting smoking, you increase your chances of keeping up with non-smoking. Stopstone is a smoking cessation app specifically for young people. Stopstone helps you through difficult moments with motivators, tips and other tools. In Stopstone:<br>- Know what you're doing it for! Stop and think about your motivation to quit smoking!<br>- See how much money you will save by not smoking<br>- Promise yourself a reward<br>- Prepare for difficult moments<br>- Ask friends and family to help you via a Helpline<br>- Smoked anyway? Learn from it and keep going!<br><br>I assume that the three things he mentioned just before (<-) created that image in him.

Doet	Reden
Gaat terug naar het zoekresultatenoverzicht	–

4	List of search results

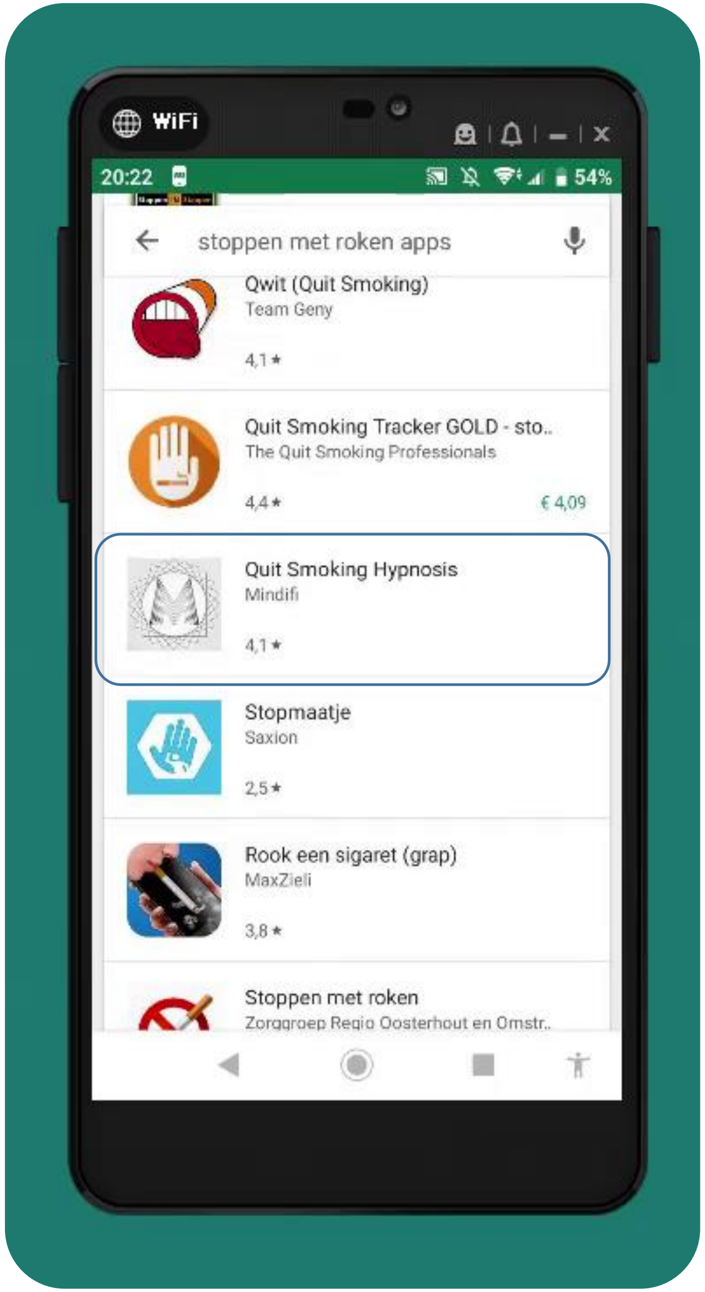

	Ziet/leest	Reageert	Information Cue
1	Hypnosis	"Then let's see, yeah. [silence] Ehm [silence] And then you have the... Here I am then also, I find that funny too, such a hypnotic..."	Naam app
2			
3			

Functionaliteit App	Opmerking onderzoeker
• ...	The participant seems to always choose apps in the search results overview that pique his curiosity, for various reasons. So far:<br>• Funny icon (app #1)<br>• Developer name (app #2)<br>• Functioning of app - hypnosis (app #3)

Doet	Reden
Scrolt naar beneden tot app #32	–
Klikt op 29de app in de lijst (Quit Smoking Hypnosis, Mindifi, #3)	"That would make me think of, well, how does that work, I'm curious about that too."

3.1 Description

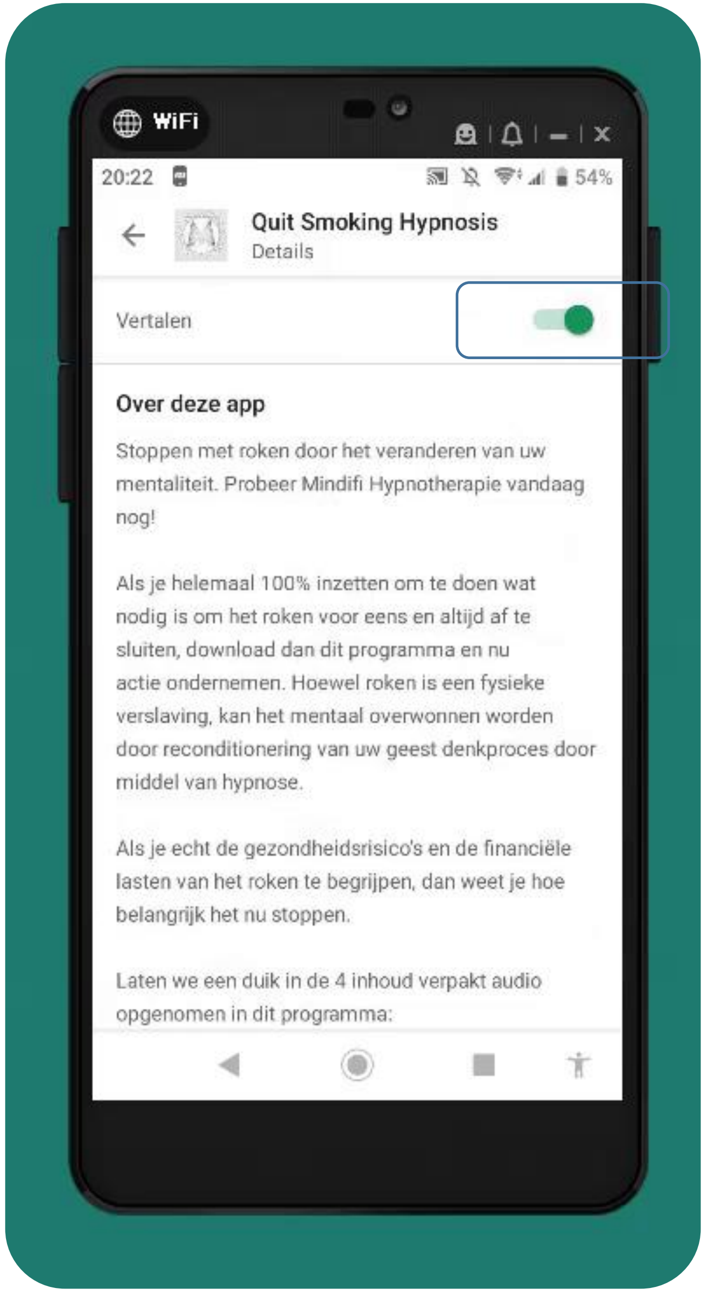

	Ziet/leest	Reageert	Information Cue
1			
2			
3			

Functionaliteit App	Opmerking onderzoeker
• …	–

Doet	Reden
• Klikt op 'Meer lezen'<br>• Gebruikt 'Vertalen'	–

3.2 Beschrijving

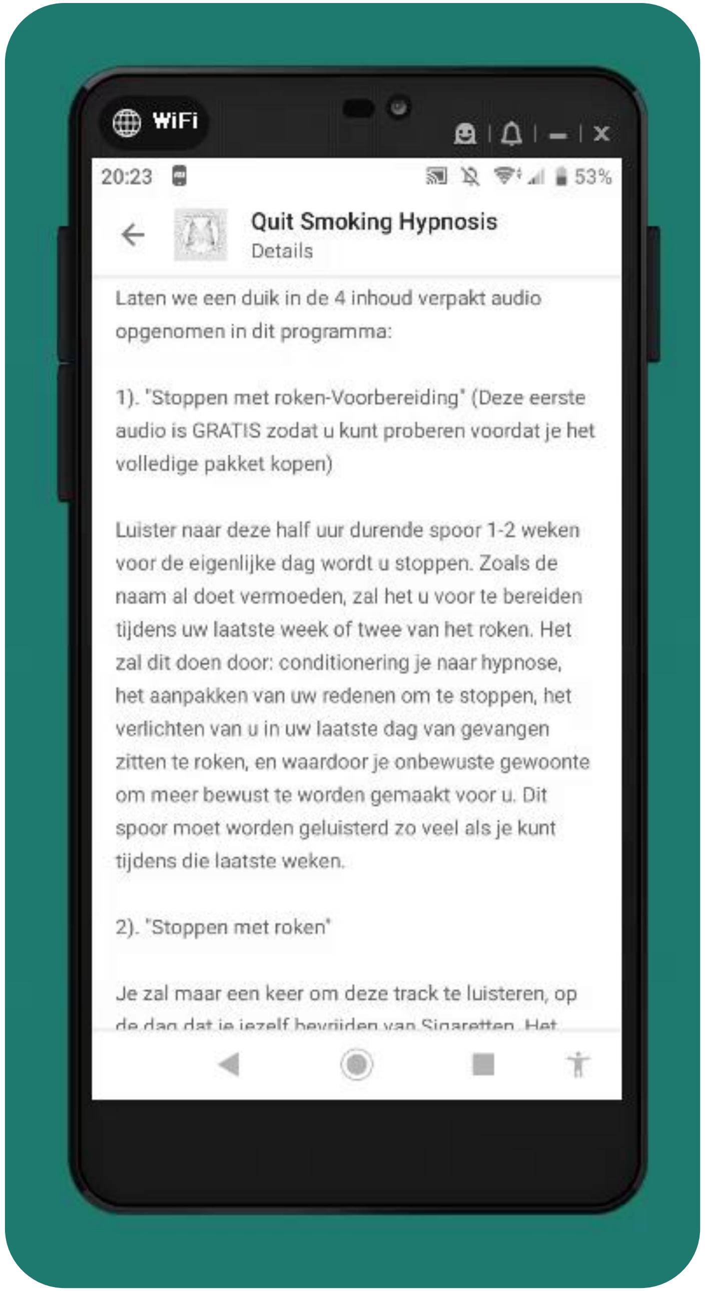

	Ziet/leest	Reageert	Information Cue
1	audio	Then you get real audio... Ah yes. Then you really get a half-hour... [laughs] [#00:50:29-7#]<br>I: What do you think now...?<br>R: Yes, that you get hypnotized. There are people who can do that, but I think I'm a little too sober for that, or have the patience for that. I think after ten minutes I'm already distracted. I don't think I'm hypnotized that easily. But then it's funny that such a thing exists.	Beschrijving
2			
3			

Functionaliteit App	Opmerking onderzoeker
• Informatie (audio)<br>• Hypnose	Participant discovers functionalities he hadn't thought of himself while searching. such as here hypnosis ("[...] it's funny then that such a thing exists."

Doet	Reden
• Gaat terug naar het zoekresultatenoverzicht	Then again, I've seen that from, that wouldn't be for me.

5	List of search results

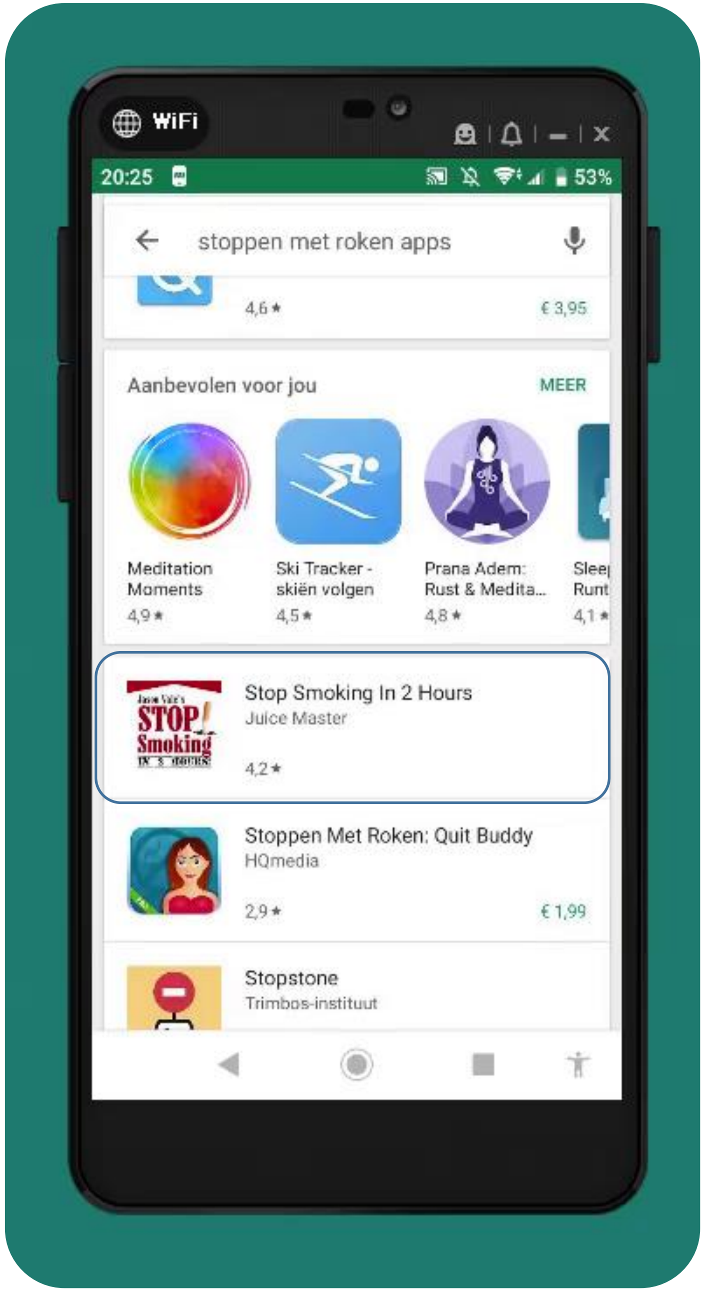

	Ziet/leest	Reageert	Information Cue
1	Stop smoking in 2 hours	Oh yes, here, an English "Stop Smoking in 2 hours". Then I thought too...	Naam app
2			
3			

Doet	Reden
Scrolt naar beneden tot app #60 en weer terug omhoog	"What else do you have? [silence] I just saw one, but I don't remember which one it was. Oh yeah, here, an English one "Stop Smoking in 2 hours". Then I thought too... [#00:51:59-4#]"
Klikt op 11de app in de lijst (Stop Smoking in 2 Hours, Juicemaster, #4)	I: Why do you click on it now? Why does that one appeal to you? [#00:52:03-2#] R: Well, just. That that could be done in two hours. Then I'm curious about that too. [Laughs] That would be the easiest thing, of course, if that could be done.

Functionaliteit App	Opmerking onderzoeker
• ...	Again, seems surprised by (how the) app works - is curious.

4.1	Description

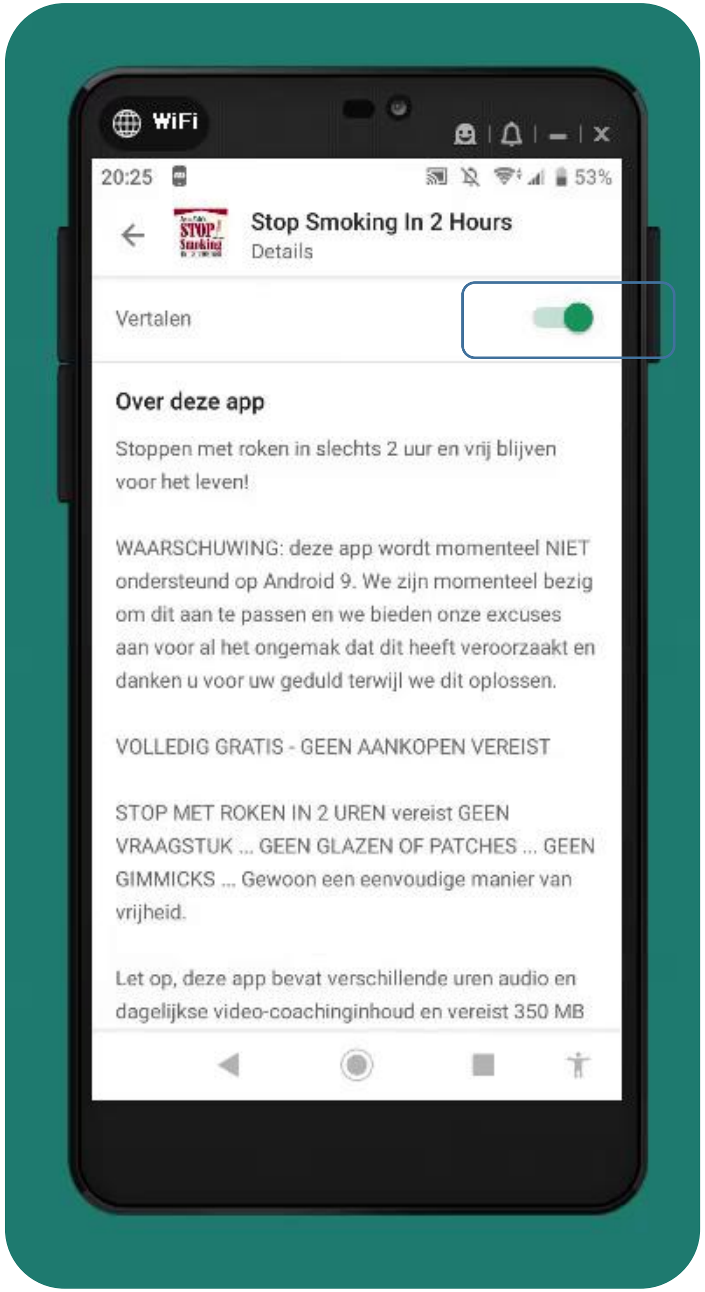

	Ziet/leest	Reageert	Information Cue
1			
2			
3			

Functionaliteit App	Opmerking onderzoeker
• …	–

Doet	Reden
• Klikt op ‘Meer lezen’<br>• Gebruikt ‘Vertalen’	–

4.2 Description

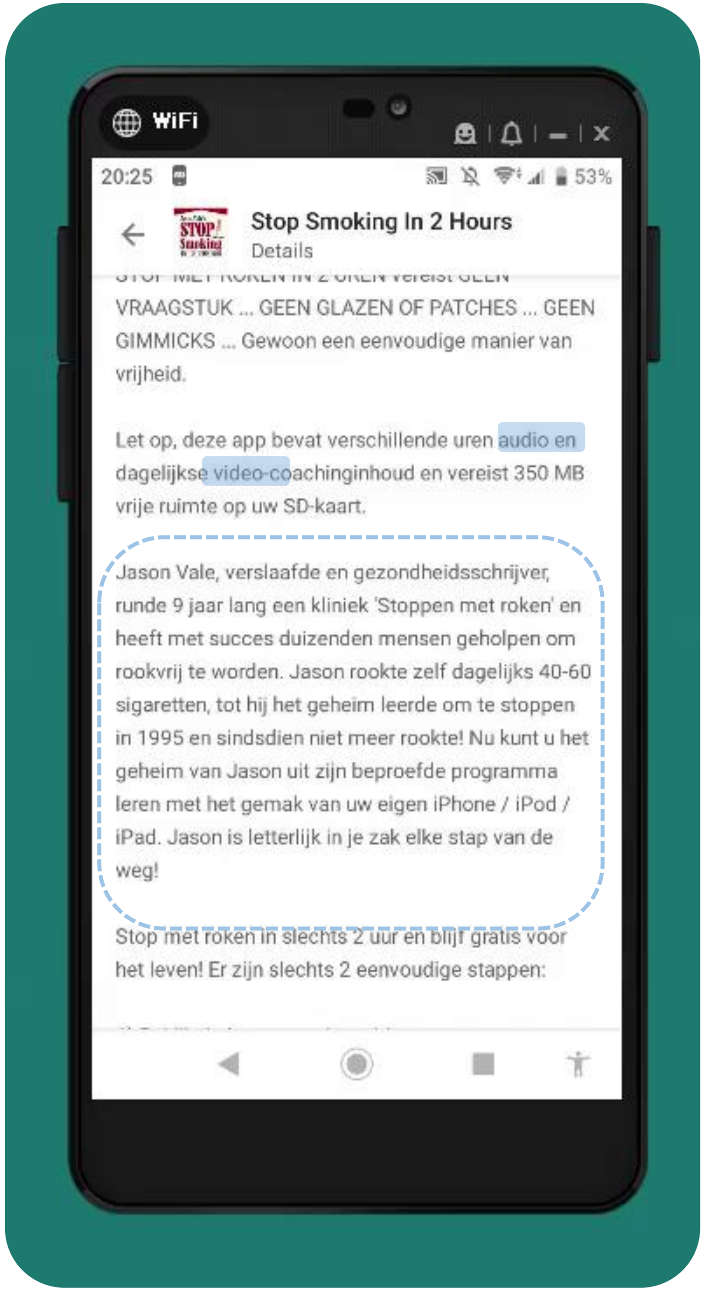

	Ziet/leest	Reageert	Information Cue
1	Audio en video	"Oh, then you do get real video and audio. Yeah, then of course you become an um..."	Beschrijving
2	?	"Then you just get stories from people, and then you get there.... Let's see. I don't know if I really... "	Beschrijving

Functionaliteit App
• Informatie (audio)<br>• Informatie (video)<br>• Informatie

Opmerking onderzoeker
On point 2. - It seems like the participant reads the paragraph that starts with "Jason Vale, addict and ...", and in response says, "Then you just get people's stories. By what he says next, it seems like he thinks that in the app you're going to get stories from other people and that that's how the app is going to help you quit smoking and that that's not for him. However, that paragraph in the description says nothing about the app but is just a piece to indicate that the developer has also been an inveterate smoker and has experience in helping people quit smoking.<br><br>In the "routine": "sees something, interprets it, decides if it is for him" something might go wrong here (again) because he makes a mistake in the step "interpret" and therefore the "decision" is based on a mistake.

Doet	Reden
	–

4.3 Description

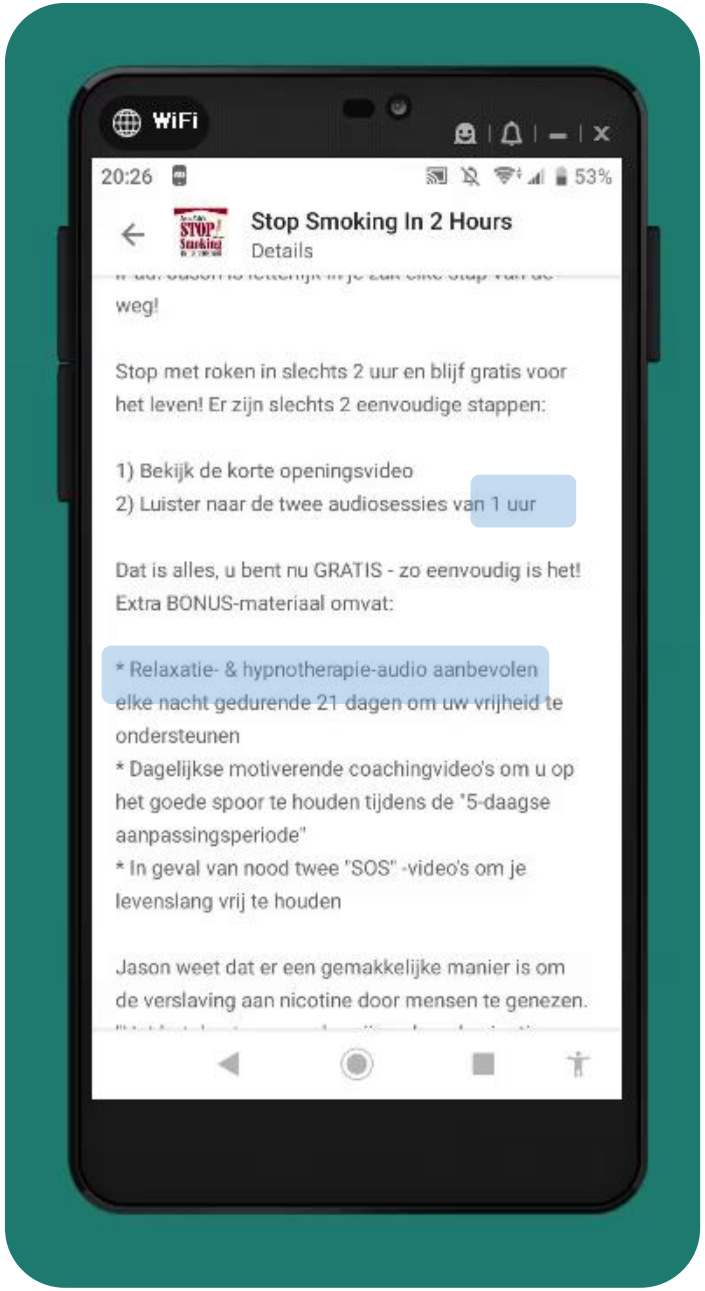

	Ziet/leest	Reageert	Information Cue
1	hypnotherapie	"That's also kind of like hypnotherapy or something."	Beschrijving
2	1 uur	"I don't think I have the patience for that either, that I would sit and listen to someone's story for an hour. And then to stop. Then I would also quickly click away. "	Beschrijving
3			

Functionaliteit App
• Hypnotherapie

Doet	Reden
• Gaat terug naar het zoekresultatenoverzicht	–

On information cues (1/3)

Information cue	Vraag Interviewer	Antwoord Respondent
	I notice that you look at what the app does, how it works, eh, that's how you call it. But are there other things that you also find important about an app? To know? [#00:53:39-1#]	R: No, no, not really that. Yeah, how it works and what it does the. [#00:53:45-1#]
Prijs	I: The price, for example? [#00:53:50-0#]	R: Yes, also, I think. I actually never do much with paid apps. I wouldn't really do that so quickly... Then I would first just start with a free app and then see if it's well.... I actually have that with all the apps I use. That you don't have the feeling of.... Why should I pay for it, when there are so many nowadays that you can also find a free app. I also just saw the one, which I was the first one, which seemed interesting to me, I just saw a paying app for that as well. [#00:54:26-4#]
	I: Okay. But in the [search results] summary here, is it the case that if there's a price with it that you skip it? [#00:54:31-3#]	R: Yes, I think so, yes. Because I also saw some with pay and then I don't click that one first.... Then I would not immediately... Because you have so many different ones anyway, yeah, then you go first anyway, I think... [#00:54:49-6#]
# Downloads	I: [...] what do you actually think of these other components? [on the app detail page]	R: No, yes, that's where it says, of course, '100,000 times downloaded'. I don't really look at that quickly either, at how many times it's been downloaded. It should actually be more like, yes.... I also understand if it says 'it's only been downloaded 1,000 times', then of course it becomes... Then you can click on it, which appeals to you, then I'll read through it a little bit, but then you don't know exactly either... Of course, that probably has very few reviews as well. [#00:55:53-8#]
	I: That's more of an incidental matter for you? [#00:55:57-3#]	R: Yes, actually I do. I want to know what it does first, and then see what yes.... Actually, of course, that's also...It could also be that it's just been made.... Yes, that doesn't mean much of course.
Rating		And then yes, those stars, or those reviews, that's then of course.... You still look at that a little bit. You see that, of course, that you... But most of them you saw that most of them were already above four. [#00:56:26-3#]
	I: Yes, okay. Isn't it inconvenient then? That they almost all score high? [#00:56:30-1#]	R: [laughs] Yes, yes.
Reviews		But then again, I'm not exactly sure how that works with such, um... How that works actually, with apps and with reviews. Yes, on the other hand, it's also a bit not entirely credible. But that's the case with all reviews, I think, all websites. That you actually have to read through it a little bit, what is actually being said. [#00:56:54-6#]

On information cues (2/3)

Information cue	Vraag Interviewer	Antwoord Respondent
Reviews (vervolg)	I: What do you mean by "not credible" then? [#00:56:56-9#]	R: Yes, that there are so many of them. And everyone is asking for reviews. I don't really believe that's all true, what it says there. Of course, it's also just that it could be someone from Vietnam, who gets paid to write reviews there. I think so. Or, I don't know from what country... [#00:57:26-3#]
	I: But the things you just read, the reviews, do you believe that those...? [#00:57:29-6#]	R: No, yes, I think so too. But you have to... I don't know exactly, then, how that is. But then I want more the information that's on it... Not like 'it helped me a lot', or a review like that, that doesn't interest me very much, because I don't know how she is in life and what kind of help she needs. That's very different for me, of course. [#00:58:02-9#]
	I: Yes, okay. I think you had with the reviews that you did look at what you could get out of them as well. What were you trying to get out of the reviews? [#00:58:11-8#]	R: Well, how such an app works then. You can already read that in the description, of course, what they themselves say about it. But it is perhaps good to read in such a review how it works for the user. [#00:58:33-7#]
Screenshots	What do you think of the rest of the information on this screen? [#00:58:42-4#]	"Yeah, that's kind of the way it is then, with a package like that and [...] drawn..."<br><br>"And then here you have, yes, I hadn't actually.... Can you have another video watch which then says... "
	And on those pictures. Have you done that before? Click on those pictures and watch movies? [#00:59:55-2#]	R: No, actually not so... No. I haven't really had any thoughts about that, like I'm going to press pictures or scroll through. I actually find the information you just find under 'read more' rather more important, or at least that I find that interesting than that I have a video... Because then it becomes another promo video, often. So then I think...
	I: You're pretty text oriented actually? [#01:00:27-7#]	R: Yeah, I find that rather interesting then I think.... [#0[illegible]

Opmerking onderzoeker

The participant thinks you can watch a video (but it's a screenshot), and apparently has an immediate idea about that too ("Yeah, that's probably a... " and a little later: "Because then it'll be another one of those promo videos, often."). But at the same time he says that he "actually doesn't" click on images and videos more often. So where does the idea that it will be "another promo video" come from?

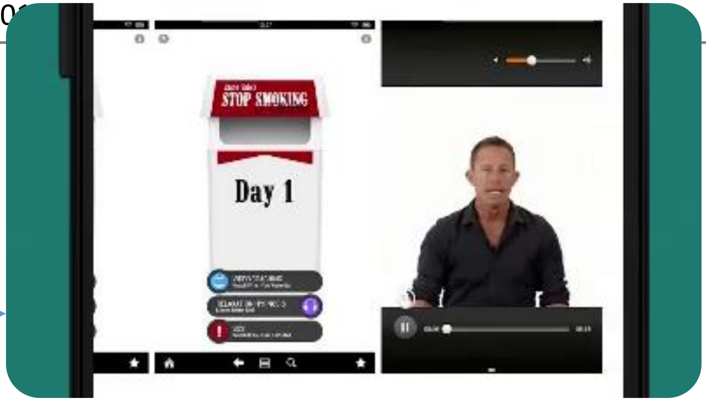

On information cues (3/3)

Information cue	Vraag Interviewer	Antwoord Respondent
PEGI<br>[PEGI (Pan-European Game Information) leeftijdsclassificatiesysteem]		
	What do you think of the rest of the information on this screen? [#00:58:42-4#]	"[...] With a 'pg3' like that, then I wouldn't know what that means either really." [screenshot 1] Hm. [#00:59:25-3#]
	I: Why are you smiling? [#00:59:25-8#]	R: No, yes, just about something from "Tom & Jerry. Oh, that's also about, um. That's then... How... It's actually a warning-type thing, such a PEGI 3. I had never actually looked at that before.

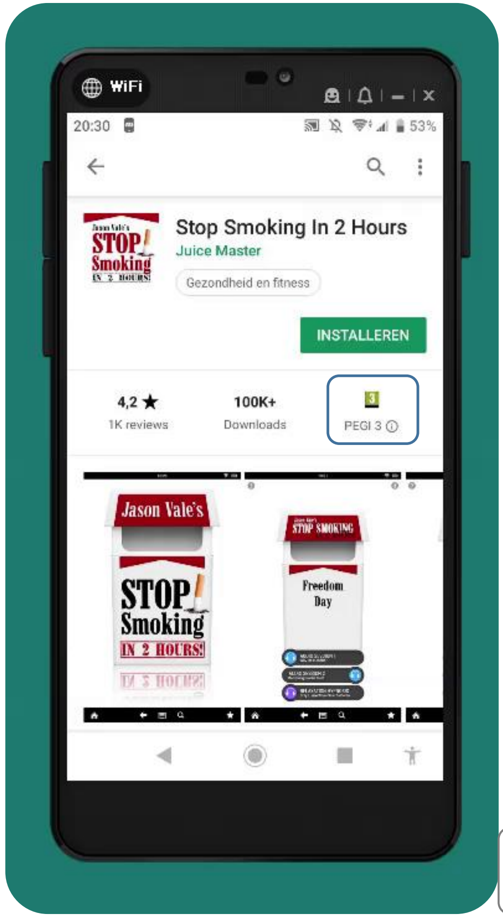

1

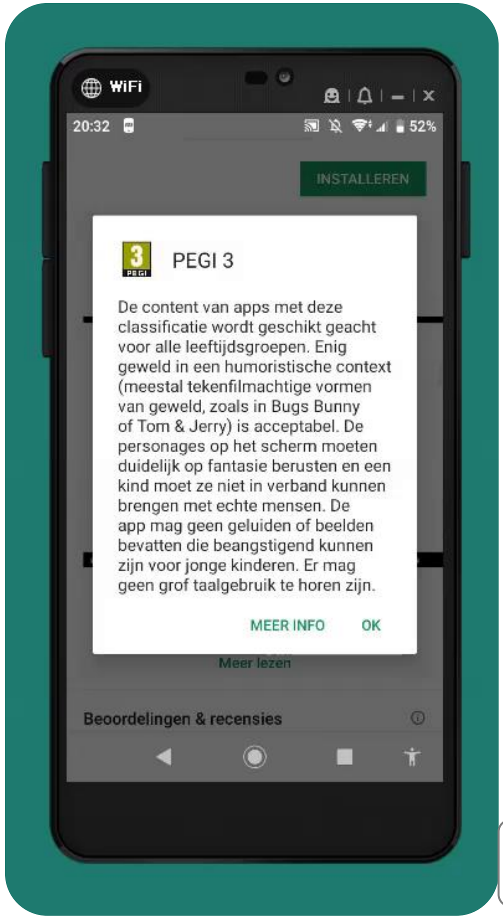

2

6	List of search results

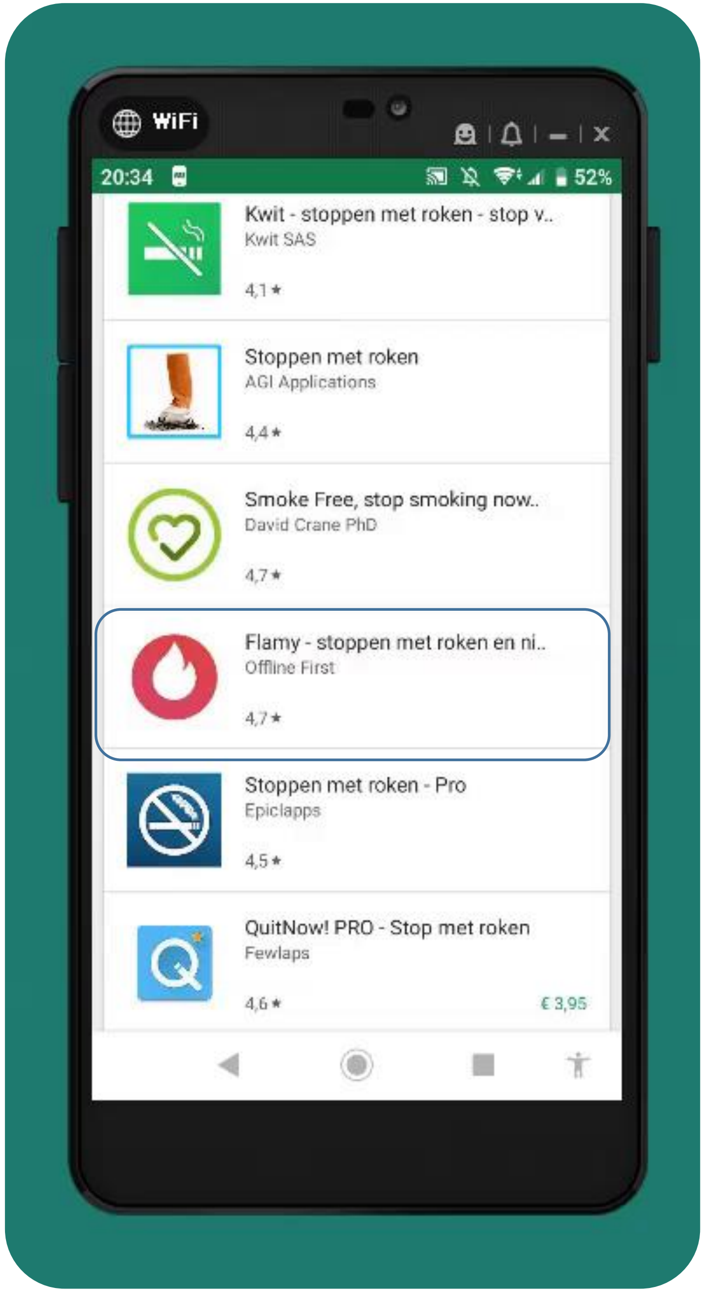

	Ziet/leest	Reageert	Information Cue
1	Flamy – stoppen met roken en ni…	I: Why are you clicking on that one? [#01:01:52-0#]<br><br>R: Yeah, I don't actually know. 'Flamy', yeah... This actually said, if you click it like that, so it doesn't say what exactly... Then you're curious maybe anyway.... It says, 'quit smoking and ni...' [#01:02:07-9#]<br><br>R: [laughs] So then you think, that might actually be smart, because then you think, 'hey, what does it say?'	Naam app
2	Offline	It also says something about "offline. I don't know if that has anything to do with it, mind you. Maybe that it's an offline app? So not that you always then throw all your data on it as well. Maybe that's another one too.... But I don't know if that's it, though, that it's for that.	Naam ontwikkelaar
3			

	Opmerking onderzoeker
• …	Misinterpretation participant when reading 'Offline'? He does clearly indicate here that he is not sure. There is no indication that he goes on to look for information on this.

Doet	Reden
• Scrolt naar app #10<br>• klikt op 8ste app in de lijst (Flamy, Hartmut Offenwanger, #5)<br>• Klikt op ‘Meer lezen’	–

5.1 Description

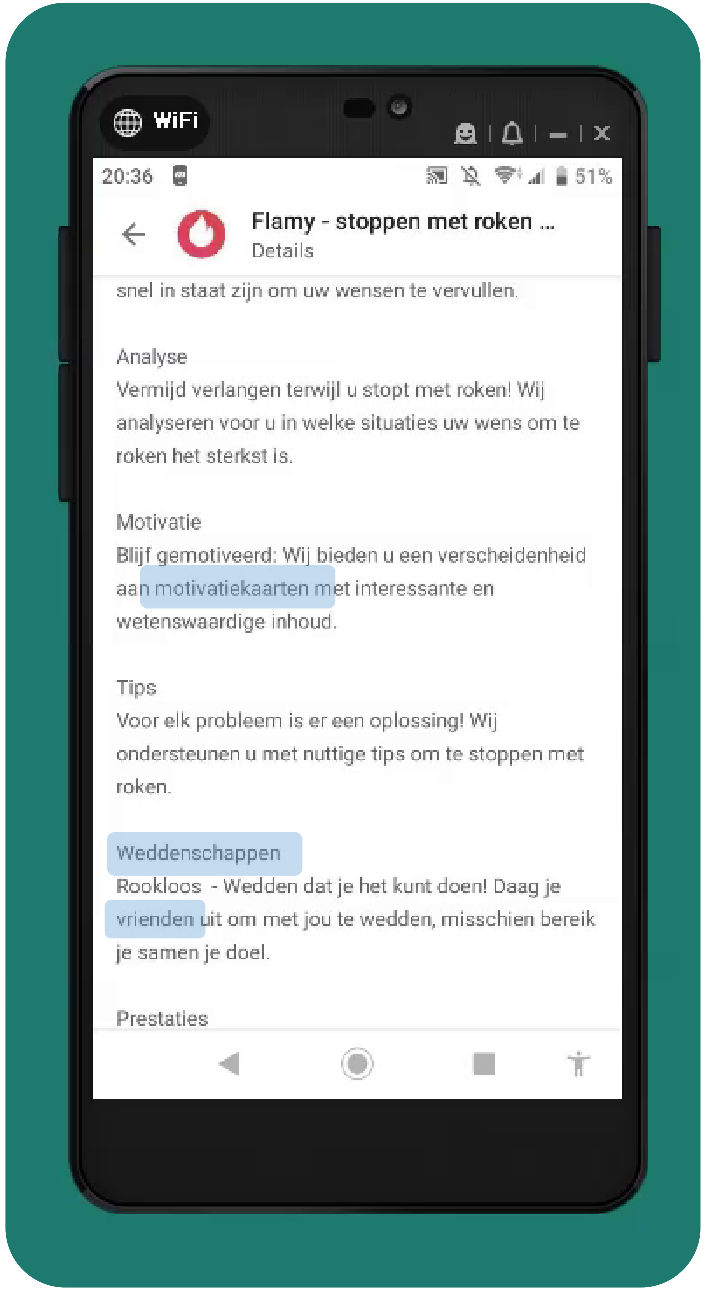

	Ziet/leest	Reageert	Information Cue
1	Motivatiekaarten	"Yes, here I am with one of those 'motivation cards', and so on. Then I think, yes, I don't have that much with that."	Beschrijving
2	Weddenschappen vrienden	"With bets with friends. Then I also think, yeah, I don't really have anything to do with that, either."	Beschrijving

Functionaliteit App	Opmerking onderzoeker
• Motivatiekaarten<br>• Weddenschappen	–

Doet	Reden
–	–

5.2 Description

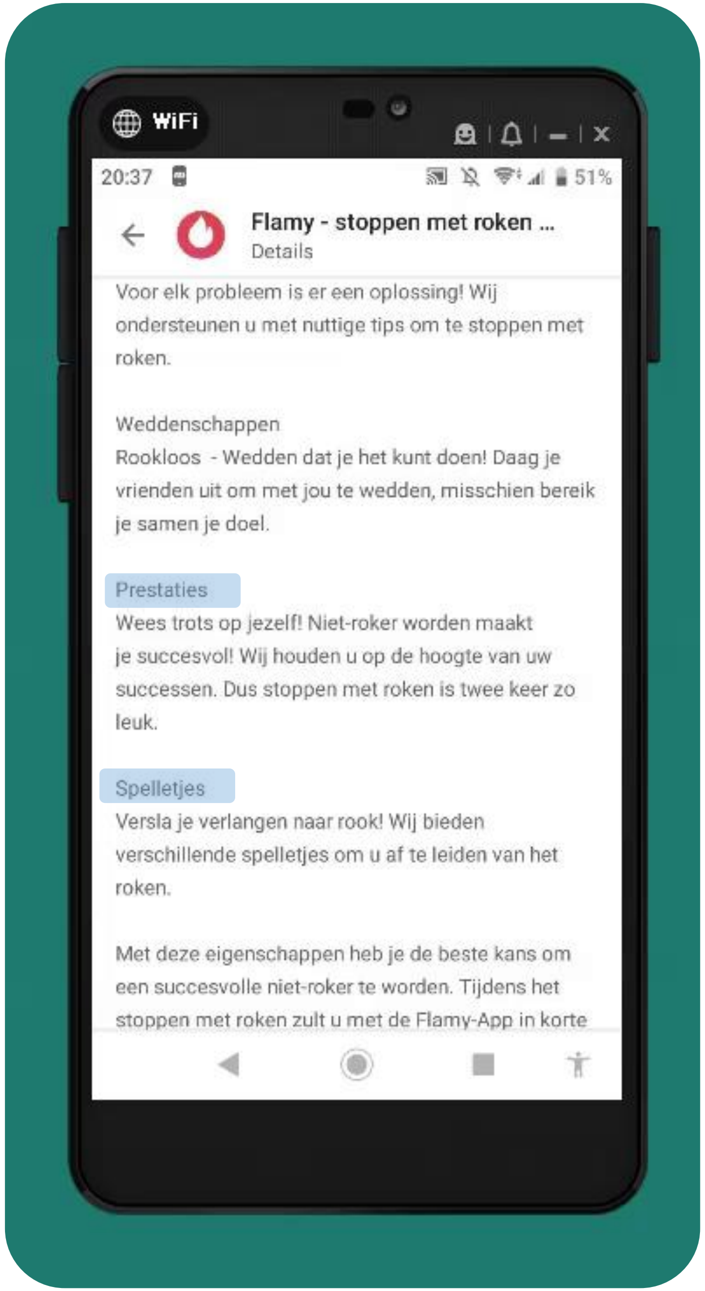

	Ziet/leest	Reageert	Information Cue
1	Spelletjes	"But then again, here's all kinds of stuff with games,....	Beschrijving
2	“Prestaties”	...and achievements. ”	Beschrijving
3			

Functionaliteit App	Opmerking onderzoeker
• ...	–

Doet	Reden
• ...	–

5.3 Description

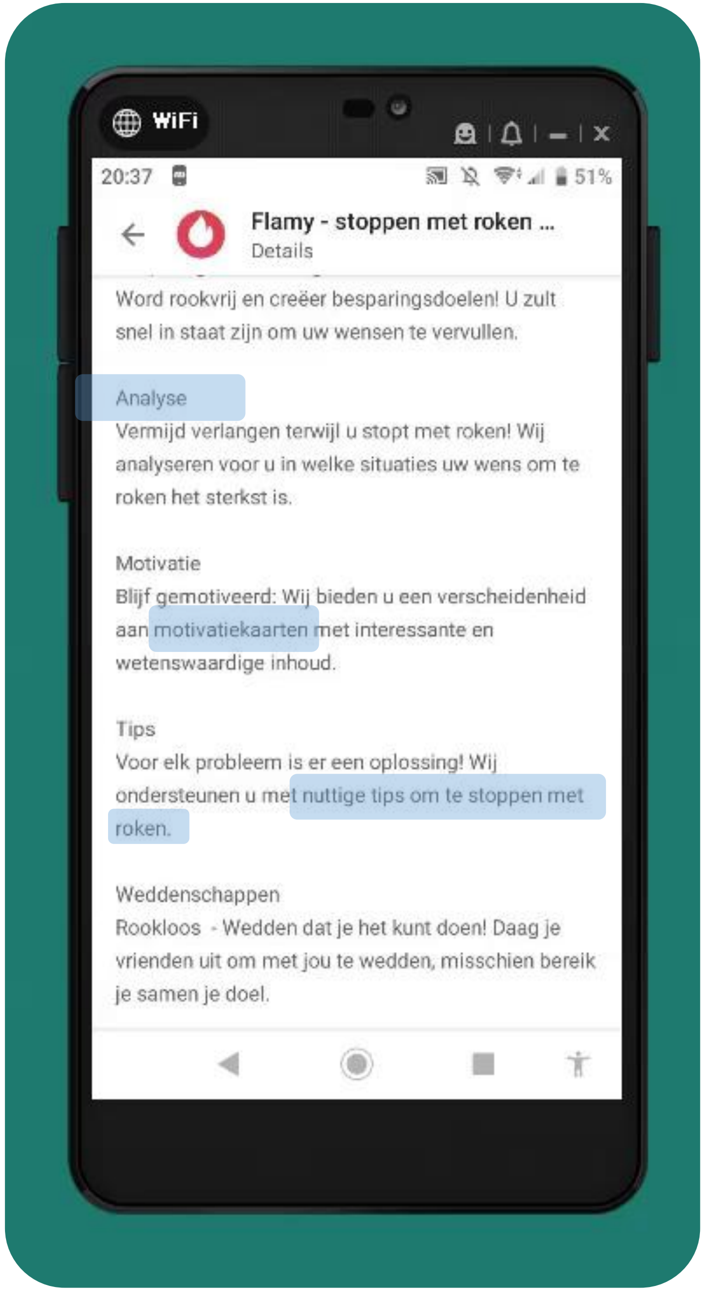

	Ziet/leest	Reageert	Information Cue
1	“nuttige tips om te stoppen met roken”	"Yes, there are some good points in it anyway, but also.... Such tips, useful tips to stop smoking, that might be another good one, of course."	Beschrijving
2	“motivatiekaarten”	"But other than that, one of those motivational cards, or something, yeah.... "	Beschrijving
	“	I: [...] But those motivational cards, why do you switch off on those? [#01:05:23-5#]<br>R: Yes, I don't know. That's another one of those motivational things, of 'you can do it', or that's what I think right away, of... [#01:05:34-0#]<br>I: Such an atmosphere? [#01:05:34-8#]<br>R: Yes, such an atmosphere. With those... Well, I have nothing to do with that. Then I immediately think, I don't need that. [#01:05:49-4#]	“
3	Analyse	"Such an analysis is then I think also nice to see, I think, what all. Well, that they analyze it, for 'in what situations your desire...'. That they can make an analysis of it. Then, of course, you'll probably have to fill in some as well, and then I think they'll.... Yes, that they might er... I do think that they have thought about it, that it is a scientific thing. Yeah, that's what I think then. "	Beschrijving

Functionaliteit App
• Tips om te stoppen met roken<br>• Motivatiekaarten<br>• Analyse (triggers)

Opmerking onderzoeker
On point 2 - This participant mentions as many as six times (slides 3, 10, 19, 23, 25 and 37) that he has nothing to do with 'motivational things', by which he means 'motivational messages' or 'motivational cards', 'push messages'. These are particularly the things he names as 'motivational'. He also says (slide 9), "Maybe game motivates", and (slides 11 & 15), "don't know if badges motivate". But for example, counters are also built in to motivate people to stop/stay stopped, and he doesn't name those (literally) as motivating. (He mainly indicates that he finds certain functionalities "funny".

5.4 Description

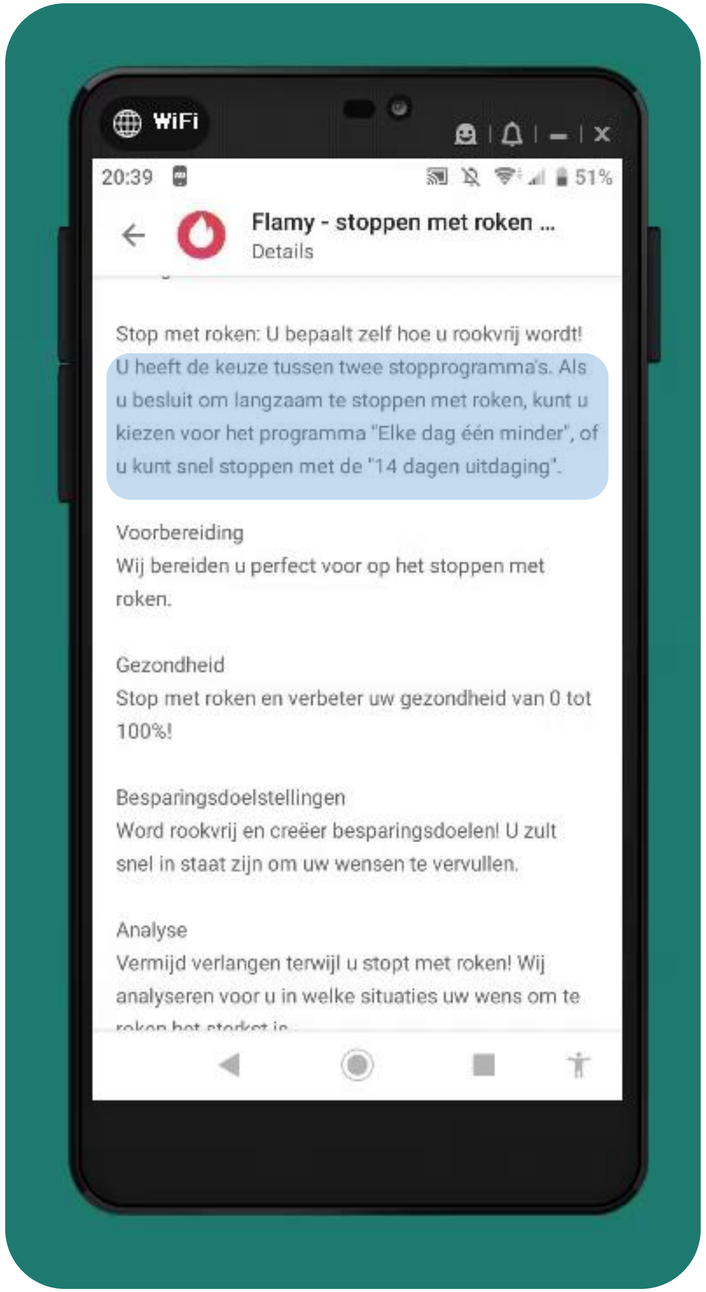

	Ziet/leest	Reageert	Information Cue
1	"als u besluit om langzaam te stoppen, u kunt kiezen voor het programma 'elke dag één minder' of je kunt snel stoppen."		Beschrijving
2	"de 14 dagen uitdaging"	Yeah, that's probably not smoking for the first 14 days then, or.... [#01:06:18-1#]	
3			

Functionaliteit App	Opmerking onderzoeker
• Langzaam stoppen<br>• 14-dagen uitdaging	–

Doet	Reden
• …	–

5.* Description

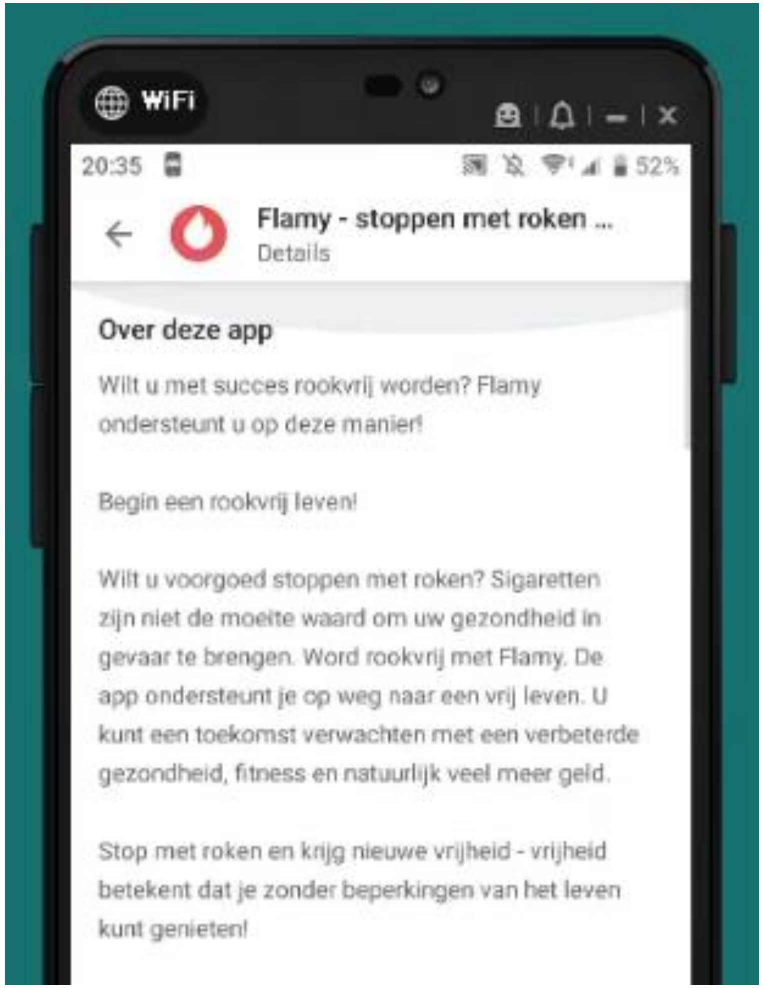

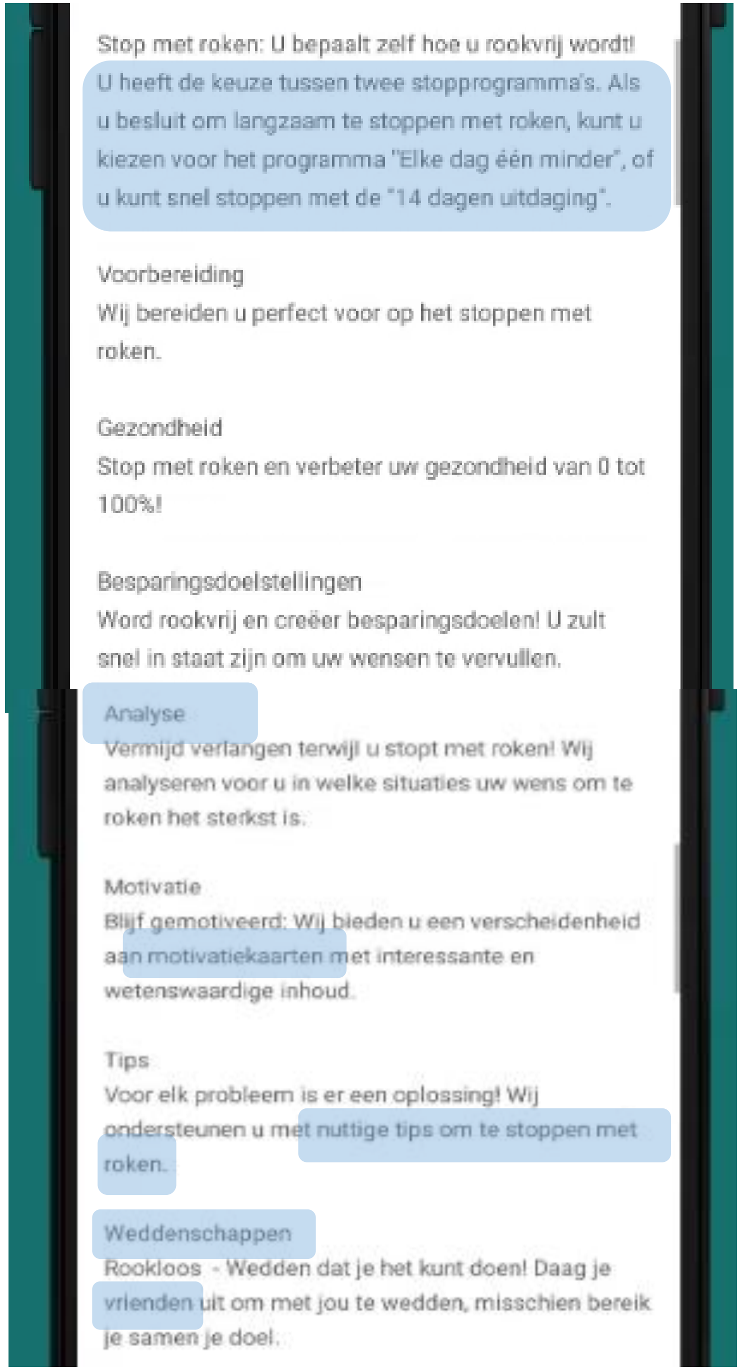

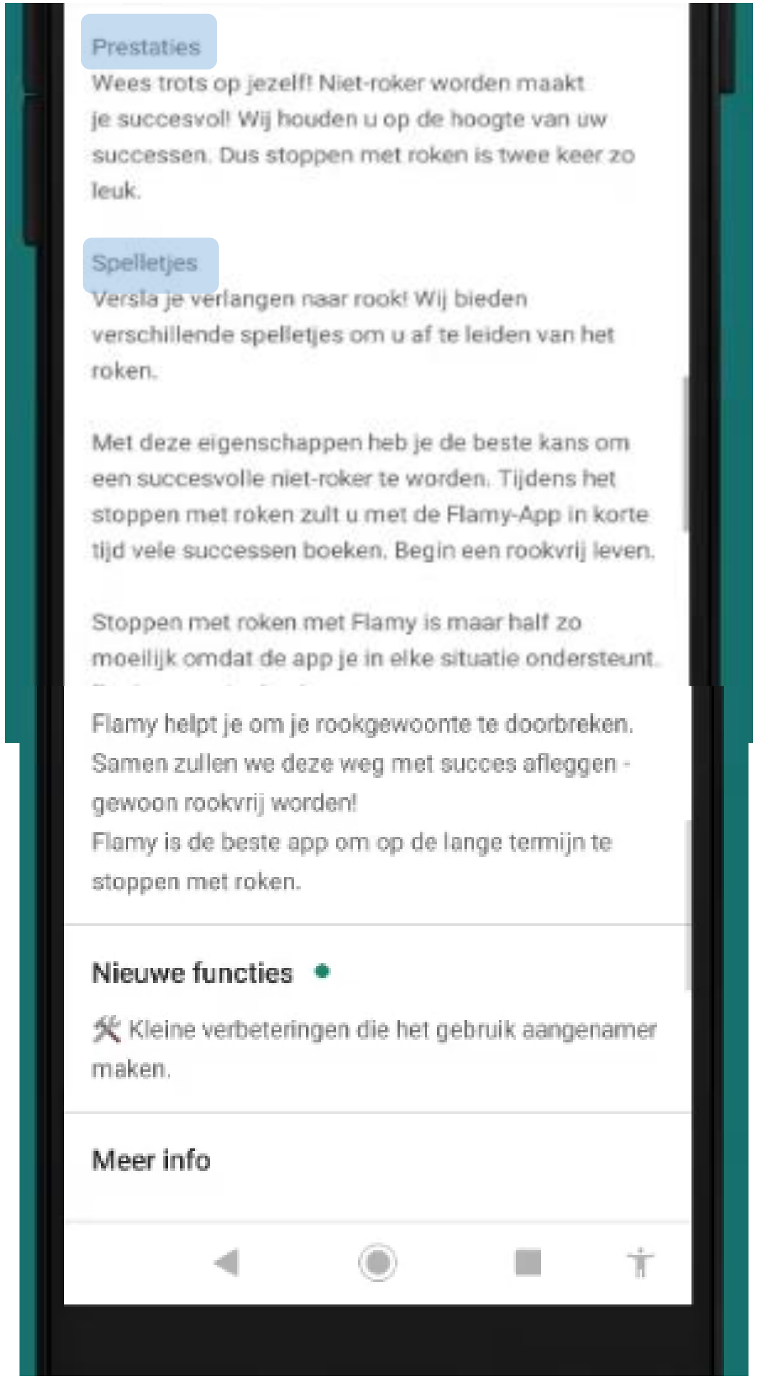

5.5 Detailed app info screen

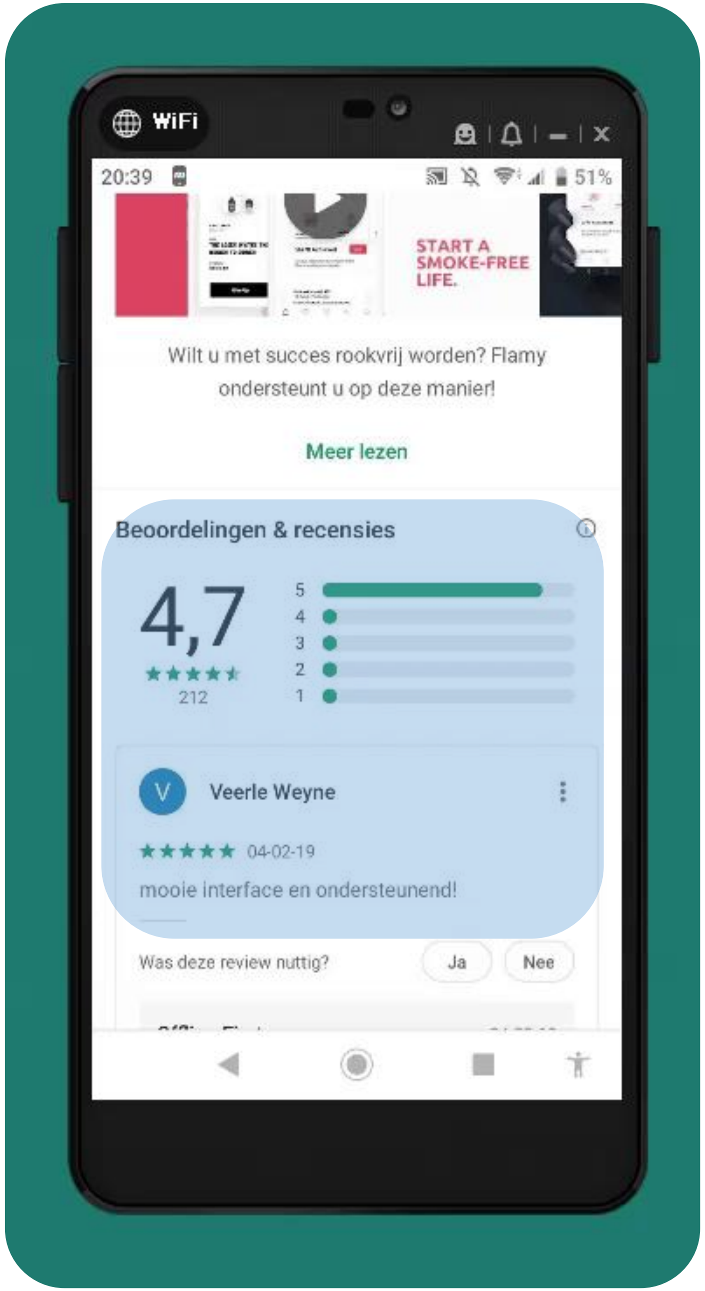

	Ziet/leest	Reageert	Information Cue
1		Then again, he does get good reviews, so that's a good thing.	Reviews
2			
3			

Functionaliteit App	Opmerking onderzoeker
• …	–

Doet	Reden
• …	–

5.6 Detailed app info screen

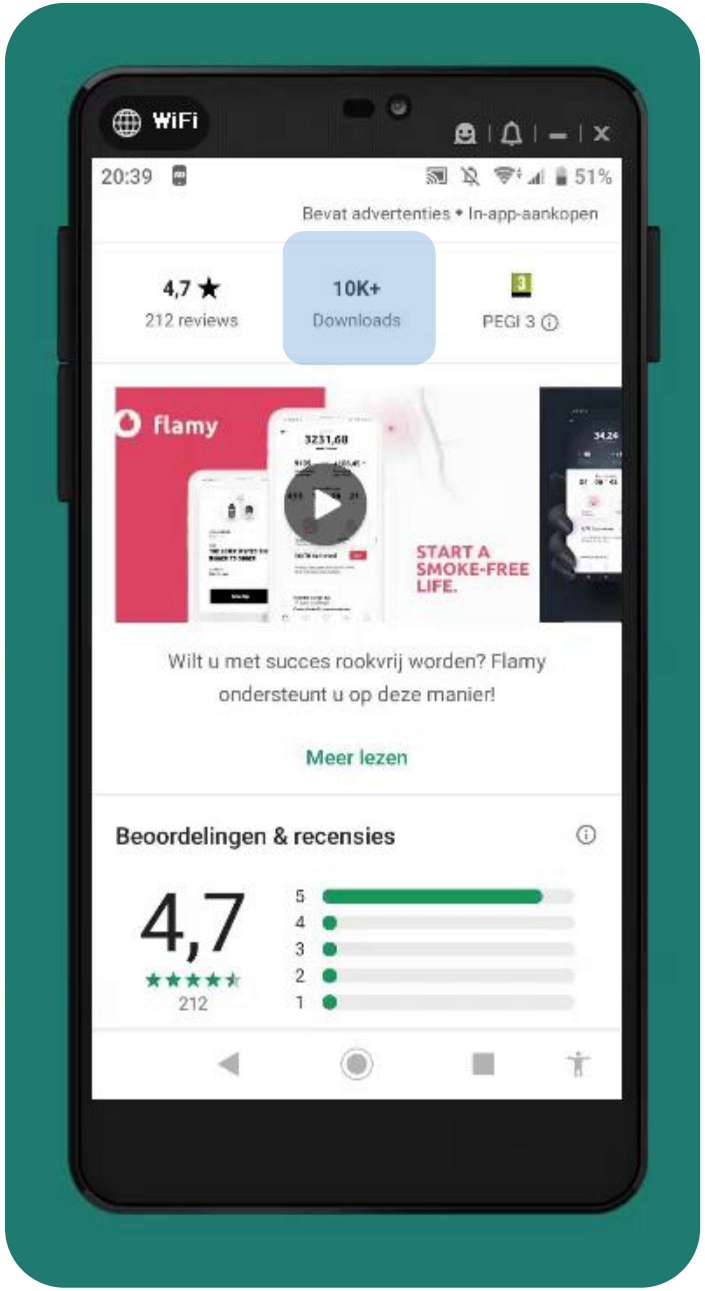

	Ziet/leest	Reageert	Information Cue
1	10K+ Downloads	It hasn't been downloaded that much yet, that's another thing.... But then that doesn't matter.	# Downloads
2			
3			

Functionaliteit App	Opmerking onderzoeker
• …	–

Doet	Reden
• …	–

5.7 Reviews

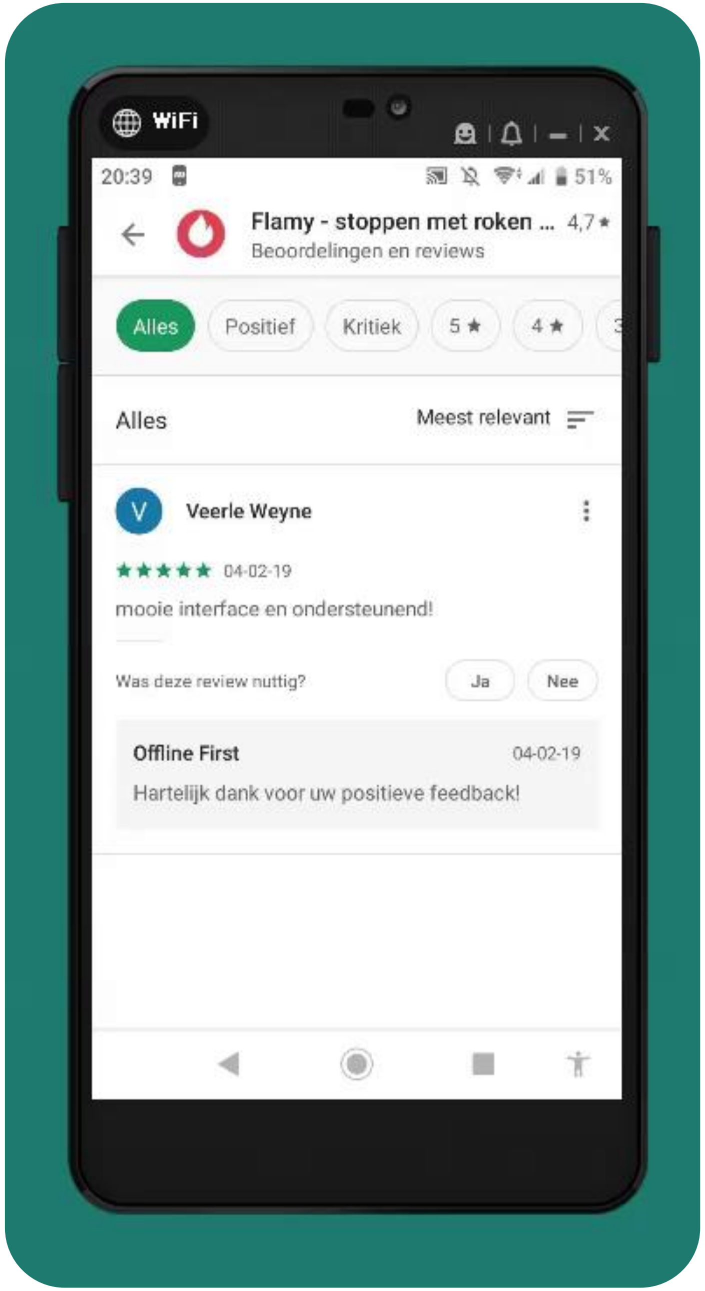

	Ziet/leest	Reageert	Information Cue
1	“mooie interface en ondersteunend”	Then again, this does say "nice interface and supportive," so that....<br><br>Oh, was that all? Or does it just seem...? [Laughs] He only has one [review], it seems. But it says '212' here anyway, so that's a bit weird. [#01:07:25-2#]<br><br>I: Yeah, I can't understand that either. But do you get a certain feeling about that? [#01:07:29-0#]<br><br>R: Yeah, then I think of, is this right, or...? R: Yeah. So that's a weird thing, too. What I just said, of, are they all a little weird.... Who give stars, but don't put anything underneath. Of course, an app developer has a company do that, [I can't quite make out, but something like:] already high in the scores, that everyone has already clicked on it once. Yes, you never know, of course, how that is, but...	Reviews<br>[& ratings]

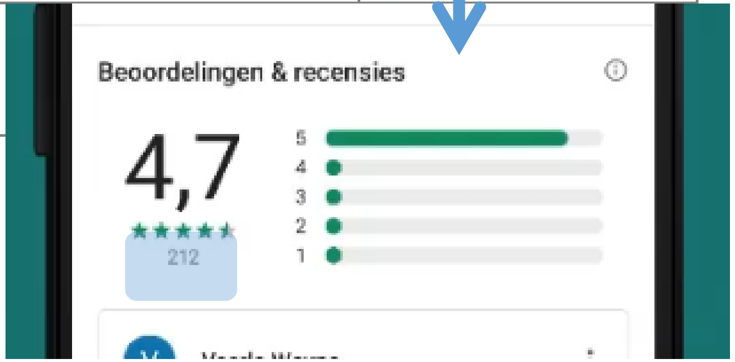

Functionaliteit App	Opmerking onderzoeker
• Mooie interface	Error in interpretation of information by participant? There are 212 reviews (ratings) and only 1 review. That's not so suspicious.

Doet	Reden
• Klikt op app van keuze (#1)<br>• Klikt op ‘Installeren’ en ‘Openen’	–

App #1 – EasyQuit free

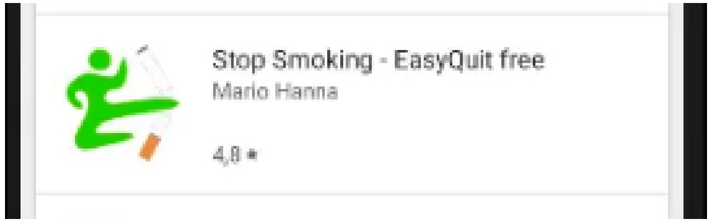

Reageert

I: I got a pretty good sense of what you're looking at. But I'm curious myself: would you look further now, or would you download something? [#01:08:09-9#]
R: Well, I would then I think first of all that ehh, where I get my first..., I would download that one first I think. Then when I've looked, and now I've already scrolled through that whole list.... [#01:08:21-0#]
I: You may also download more than one, but you... [#01:08:23-3#]
R: No, then I don't think I would either.... Because then I'll have too many things again I think. [#01:08:32-3#]...

R: [...] Then I would go for this one, I think. [#01:08:44-8#]
I: And then why, of all the ones you've looked at, why this one? [#01:08:48-8#]
R: Yeah, because I'd be the first one anyway.... Um, yeah. My first things, um, what I saw of, that triggers me, with one of those little things, puppet. But when I read it, I actually found some nice things in it that I thought were nice or interesting. Um. [#01:09:11-8#]
I: Do you still have certain doubts about this app? Doesn't have to be, but could be. [#01:09:20-0#]
R: Neu. Or yes, well, not yet. Because I still don't know then right now is how it really works. [#01:09:25-6#]
I: No, not in advance so? [#01:09:26-6#]
R: No, not in advance, no. Then what I just sat down and read from the other one.... Just saw the other one, the one I had just clicked last, there were also some things there that were the same, matched, but that ehh.... Yes, then you see that there are only a few reviews, maybe that's it... Then I think again, the first one I had, it has that too.... My first hunch often counts, of course, that that's the better one, than having looked at five different ones, I think. [#01:10:07-7#]
I: Okay. So is it that you actually opened that first one, and compared other apps to the first one? [#01:10:15-8#]
R: Well, kind of. But on the other hand, yeah... I also just naturally looked at a few things of such a hypnosis, or an ehh.... Then again I am looking at what it is then ehh.... [#01:10:28-8#]
I: That's kind of out of curiosity? [#01:10:29-8#]
R: Yes, that's also curiosity, of what would I do. But maybe, because this already appealed to me a bit, there were already some things in it that appealed to me, then I would er... Then you compare it with what eh... with the first one that you eh, yes... [#01:10:49-9#]

Functionaliteit App	Opmerking onderzoeker
• ...	

Doet	Reden
Installeert en opent de app	

App #1 – EasyQuit free

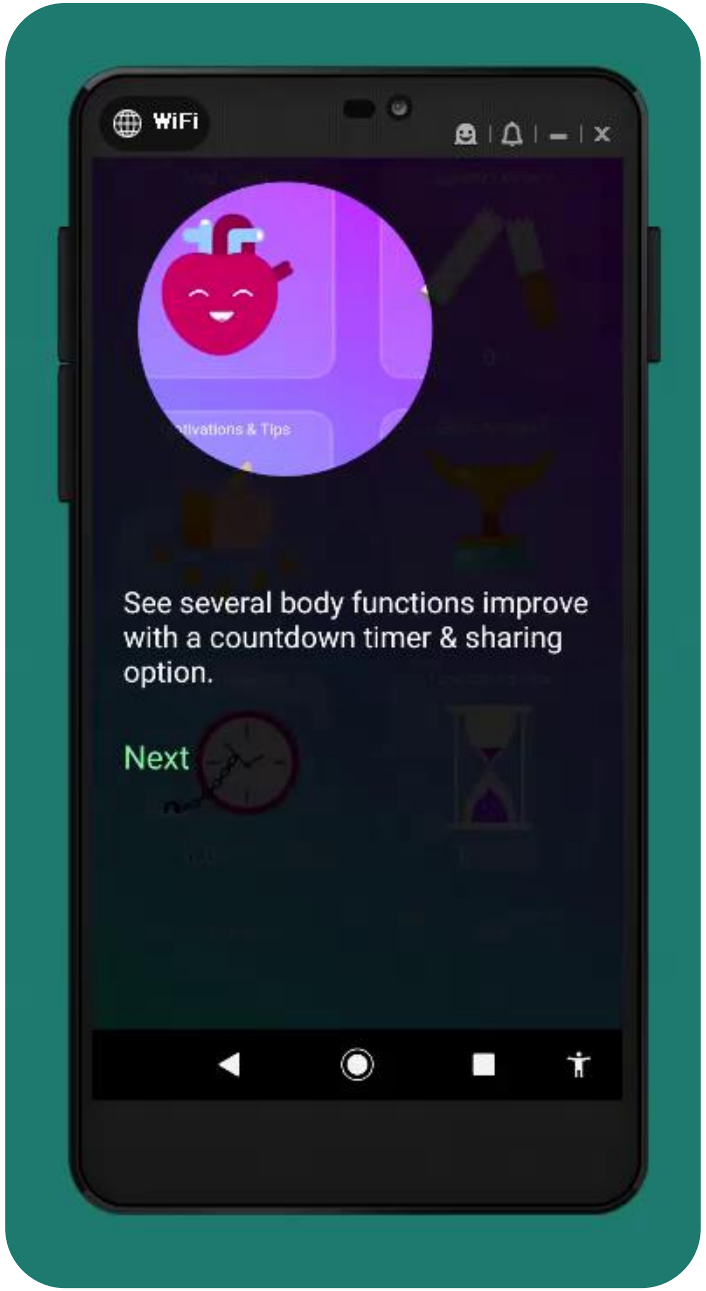

	Ziet/leest	Reageert	Information Cue
1		–	

Functionaliteit App	Opmerking onderzoeker
• …	

Doet	Reden
• …	–

App #1 – EasyQuit free

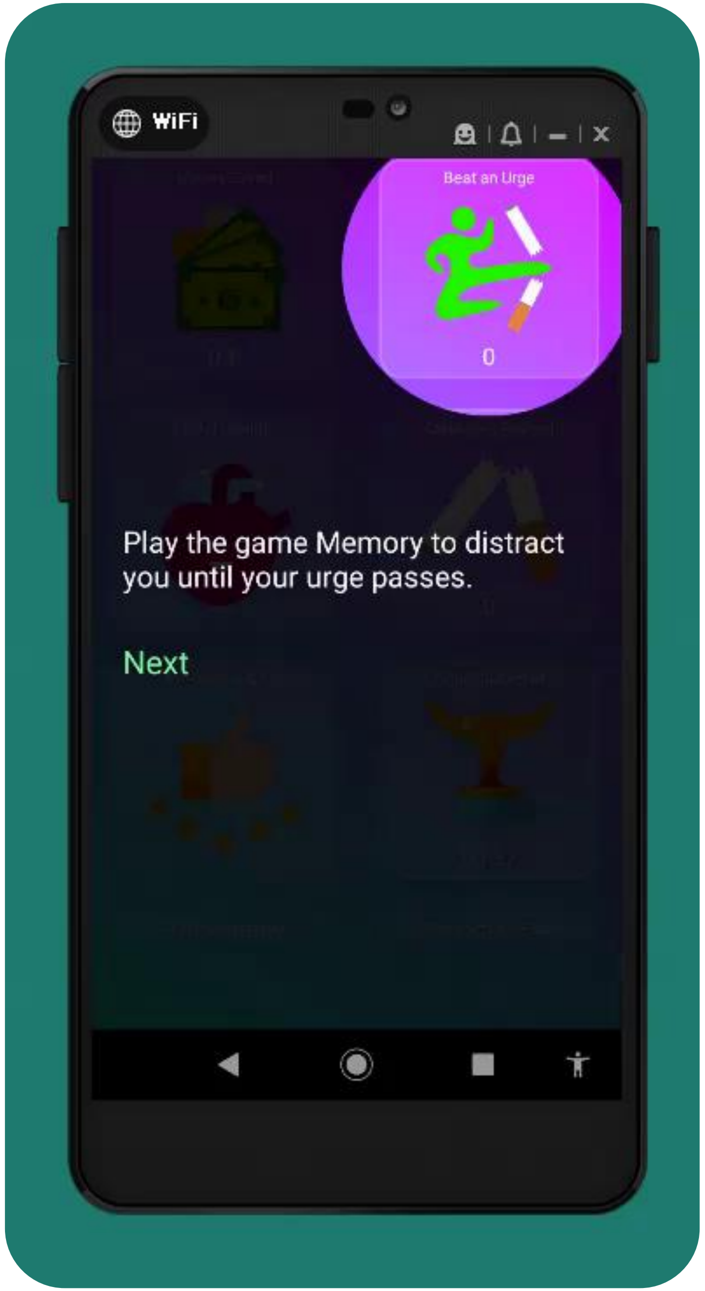

	Ziet/leest	Reageert	Information Cue
1		Oh, look, yes. So that's the game then, memory.	

Functionaliteit App	Opmerking onderzoeker
• …	

Doet	Reden
• …	–

App #1 – EasyQuit free

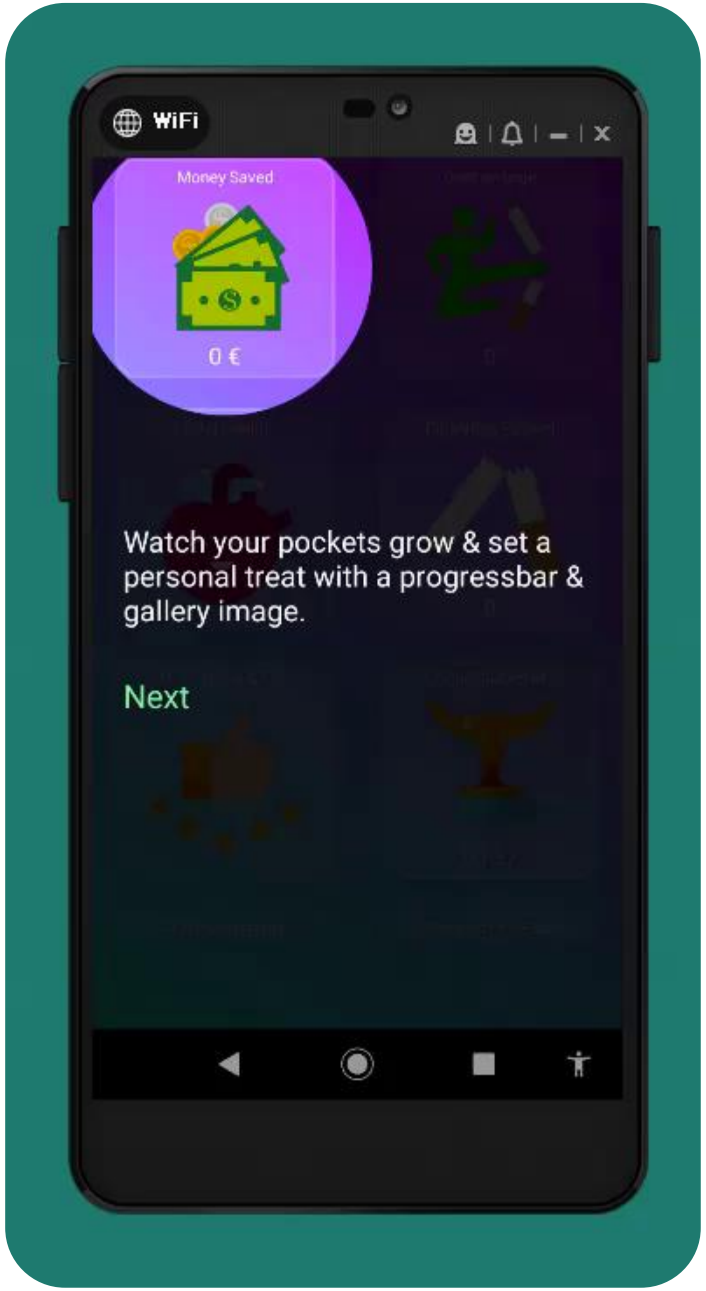

	Ziet/leest	Reageert	Information Cue
1		That's then your wallet, how much it gets filled when you quit smoking. I think that's kind of funny, kind of the older thingies, more from the eighties, nineties.... [#01:11:49-4#]<br><br>I: Icons? [#01:11:49-9#]<br><br>R: Yes, icons, that's what I find funny, that it doesn't all look very slick actually [laughs] A little Mario Bros.	

Functionaliteit App	Opmerking onderzoeker
• ...	

Doet	Reden
• ...	–

App #1 – EasyQuit free

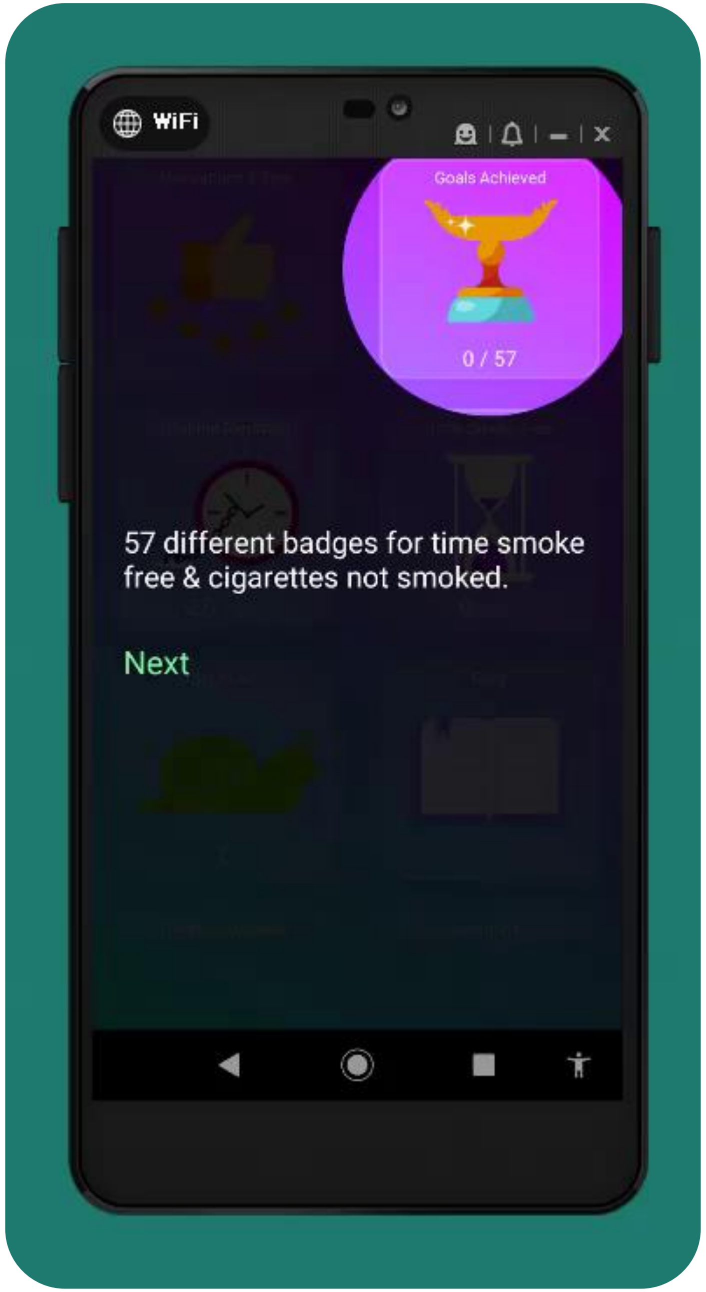

	Ziet/leest	Reageert	Information Cue
1		"These are the badges then. It does look pretty organized."	

Functionaliteit App	Opmerking onderzoeker
• …	

Doet	Reden
• …	–

App #1 – EasyQuit free

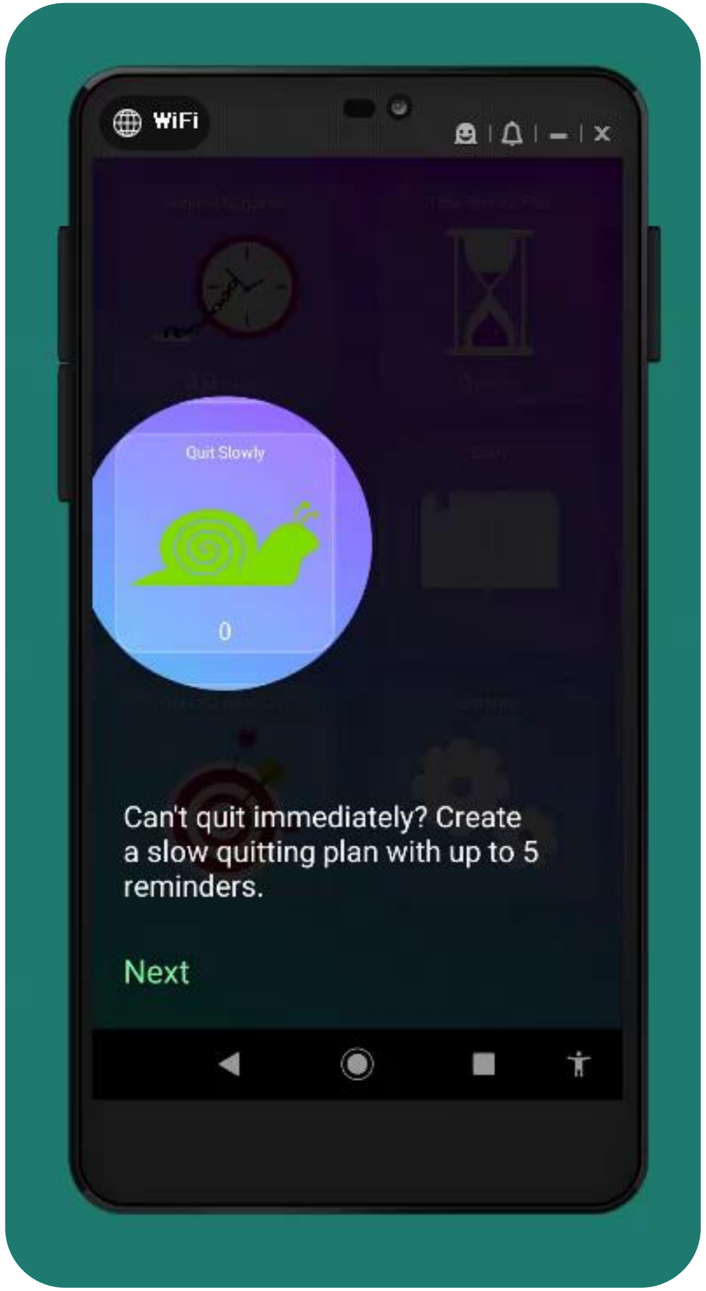

	Ziet/leest	Reageert	Information Cue
1		"And this is then um... That you can also, what's it called, slow, quietly stop. That you then have five reminders. That would be the nicest thing... That I don't get too much... That I'm not pushed all day long. That would drive me crazy, I think."	

Functionaliteit App	Opmerking onderzoeker
• ...	

Doet	Reden
• ...	–

App #1 – EasyQuit free

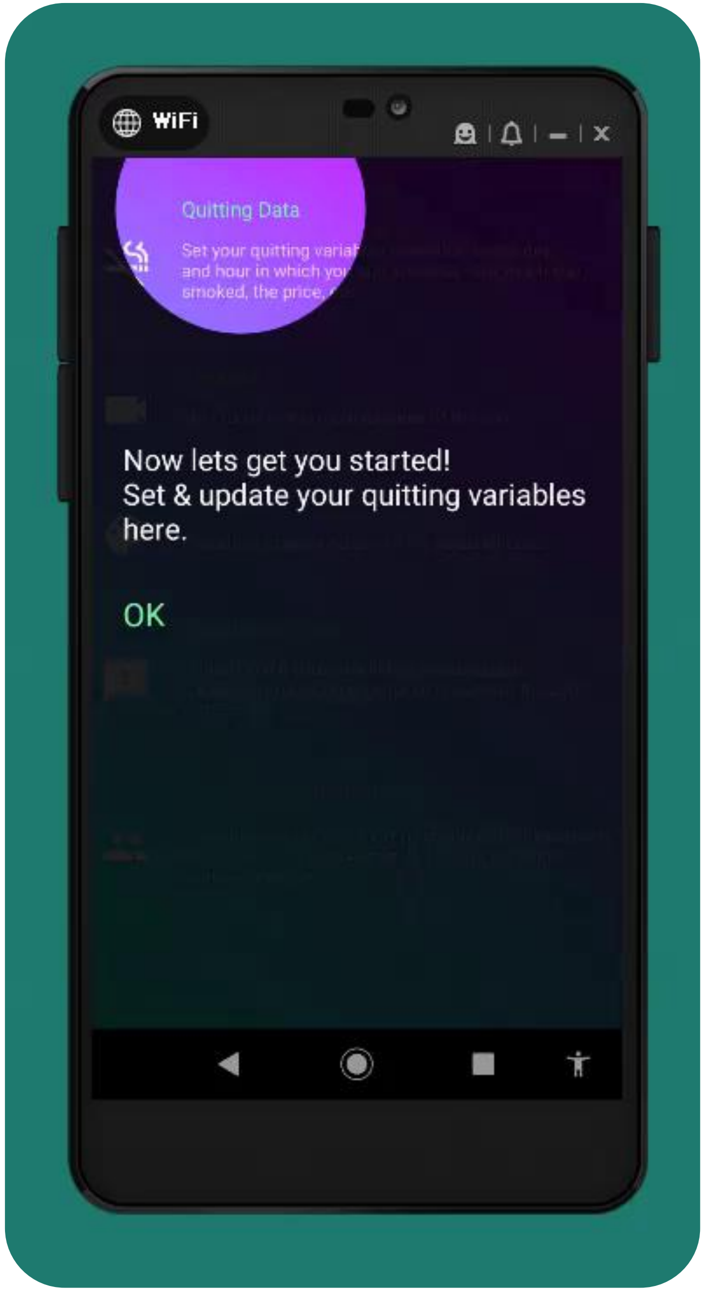

	Ziet/leest	Reageert	Information Cue
1		"Oh yeah, and then it actually starts right away with the app."	

Functionaliteit App	Opmerking onderzoeker
• ...	

Doet	Reden
• ...	–

App #1 – EasyQuit free

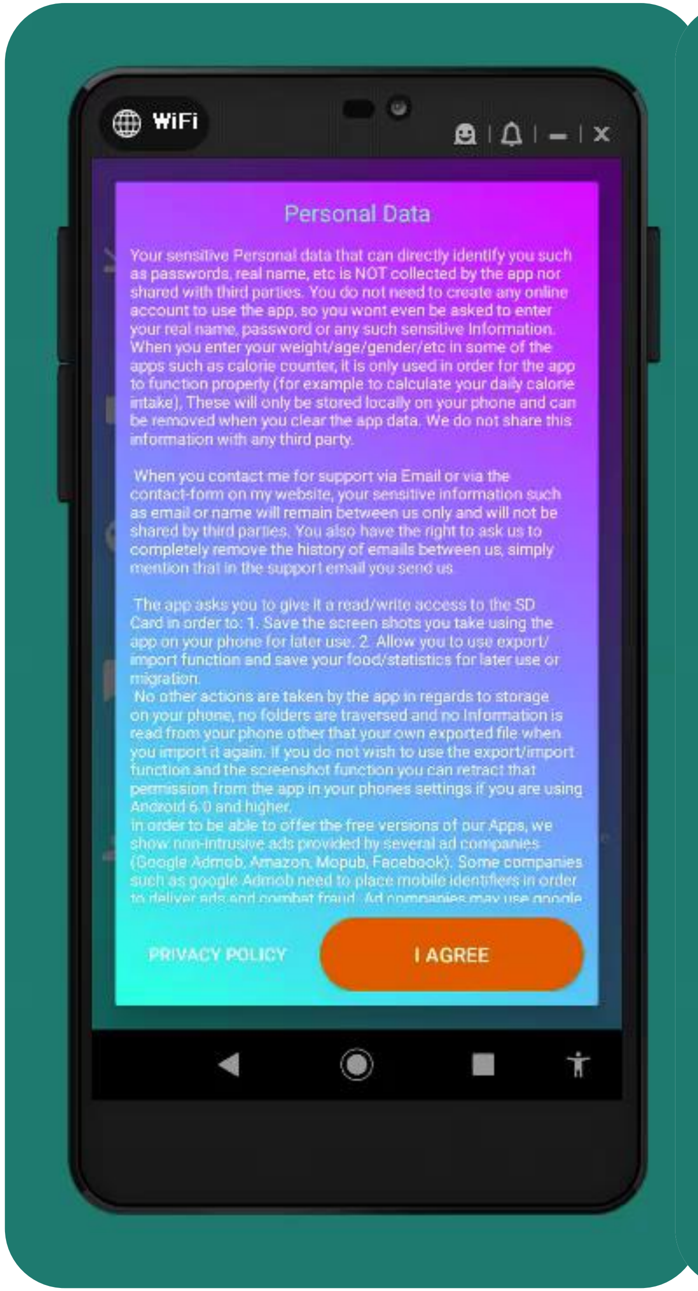

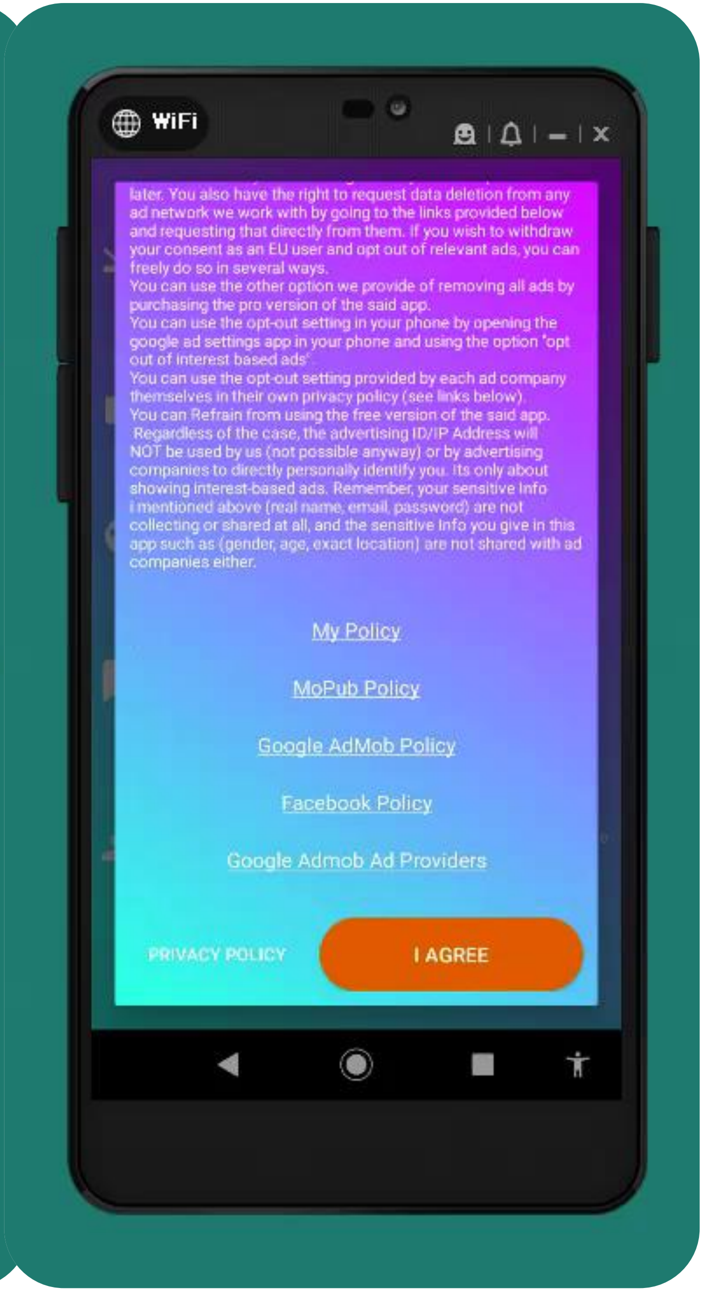

	Ziet/leest	Reageert	Information Cue
1		Hm. [silence] Yeah... [#01:13:07-9#]<br><br>I: What do you think about with this, with that screen? [#01:13:10-6#]<br><br>R: Yeah, I always do just 'agree' actually, often [laughs]. Then I don't quite feel like reading that all the way through, that ehh [#01:13:18-9#].<br><br>I: I think you kind of looked like, is it readable? [#01:13:19-6#]<br><br>R: Yeah, is it readable. Do I see something in there that I think, that stands out, maybe that's not quite.... But that ehh.... Yeah, I can always do that.... Then I think of, it'll be good. [#01:13:37-9#]	

Functionaliteit App	Opmerking onderzoeker
• ...	The participant had this text on the screen for 18 seconds before clicking 'I agree'.

Doet	Reden
• ...	–

App #1 – EasyQuit free

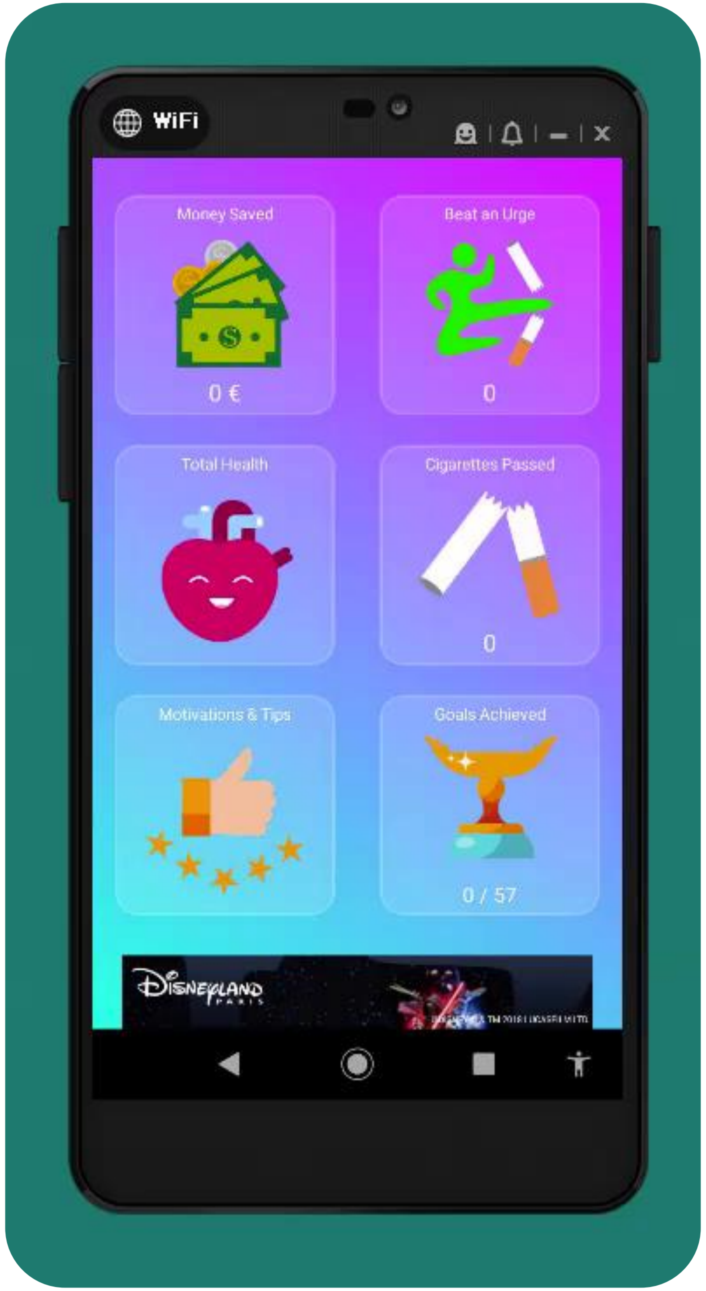

	Ziet/leest	Reageert	Information Cue
1			

Functionaliteit App	Opmerking onderzoeker
• …	

Doet	Reden
• …	–

App #1 – EasyQuit free

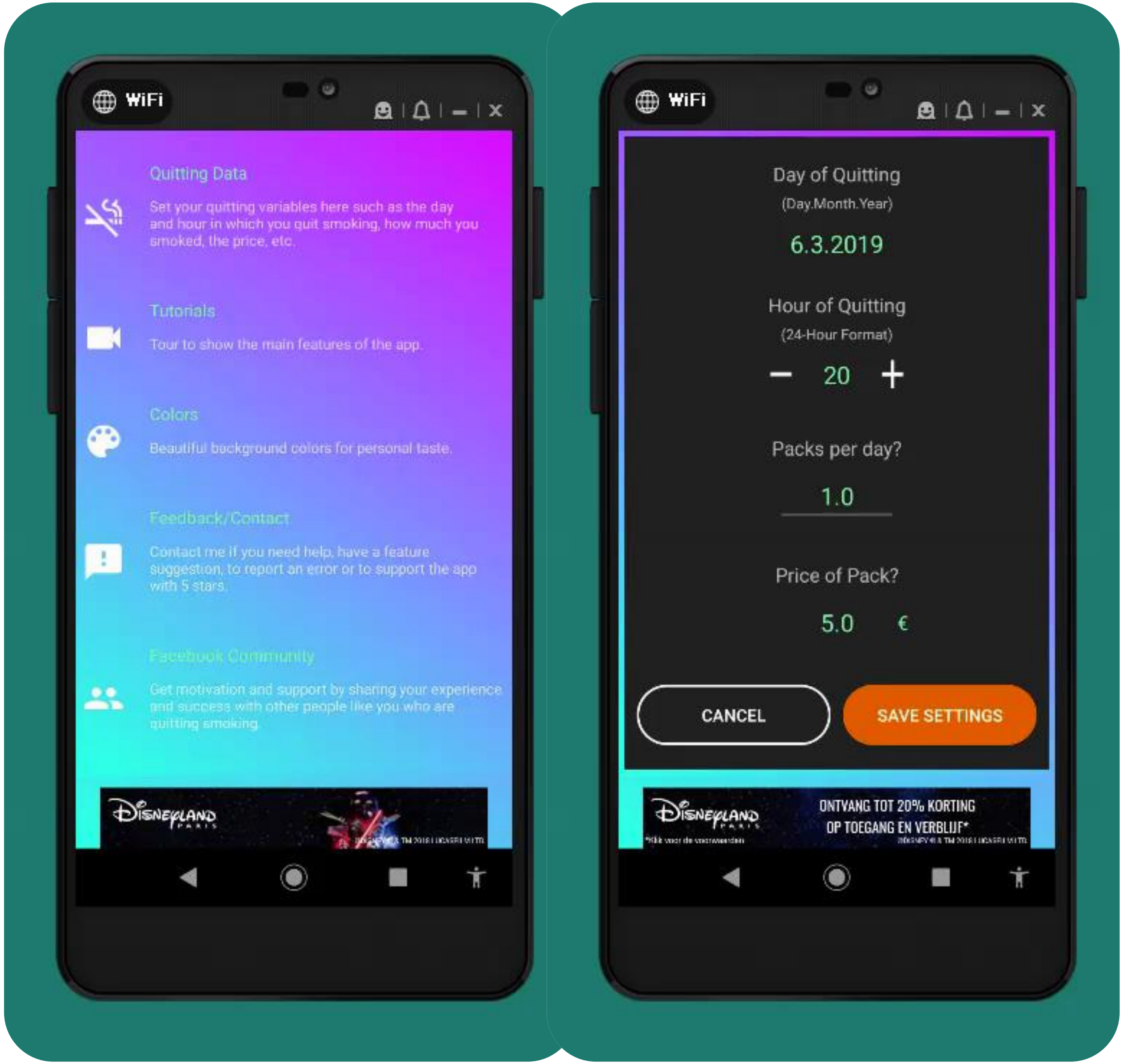

	Ziet/leest	Reageert	Information Cue
1		R: Yes, you can still adjust it completely of course. Jaha. [silence] Ehm. Because that's actually more the back, I think huh, that you can personalize it. Oh yes, so... 'Hour of quitting'. Oh yes, here you can 'm yes... Those are actually the settings. But yes, there you can so ... So I should really just look for it now, how it really, um... [#01:14:51-4#]<br><br>I: Looks... [#01:14:53-3#]<br><br>R: Yes, I would do that first then. Then I'd look at it first all the way still like that, how it works then and then I'd... [#01:15:00-3#]<br><br>I: Then set it up? [#01:15:02-3#]<br><br>R: Yes, of how many packs you would smoke, and ehh.... [#01:15:05-3#]	

Functionaliteit App	Opmerking onderzoeker
• ...	

Doet	Reden
• Klikt huidige scherm weg<br>• Zoekt app op 'bureaublad' op en opent de app	–

App #1 – EasyQuit free

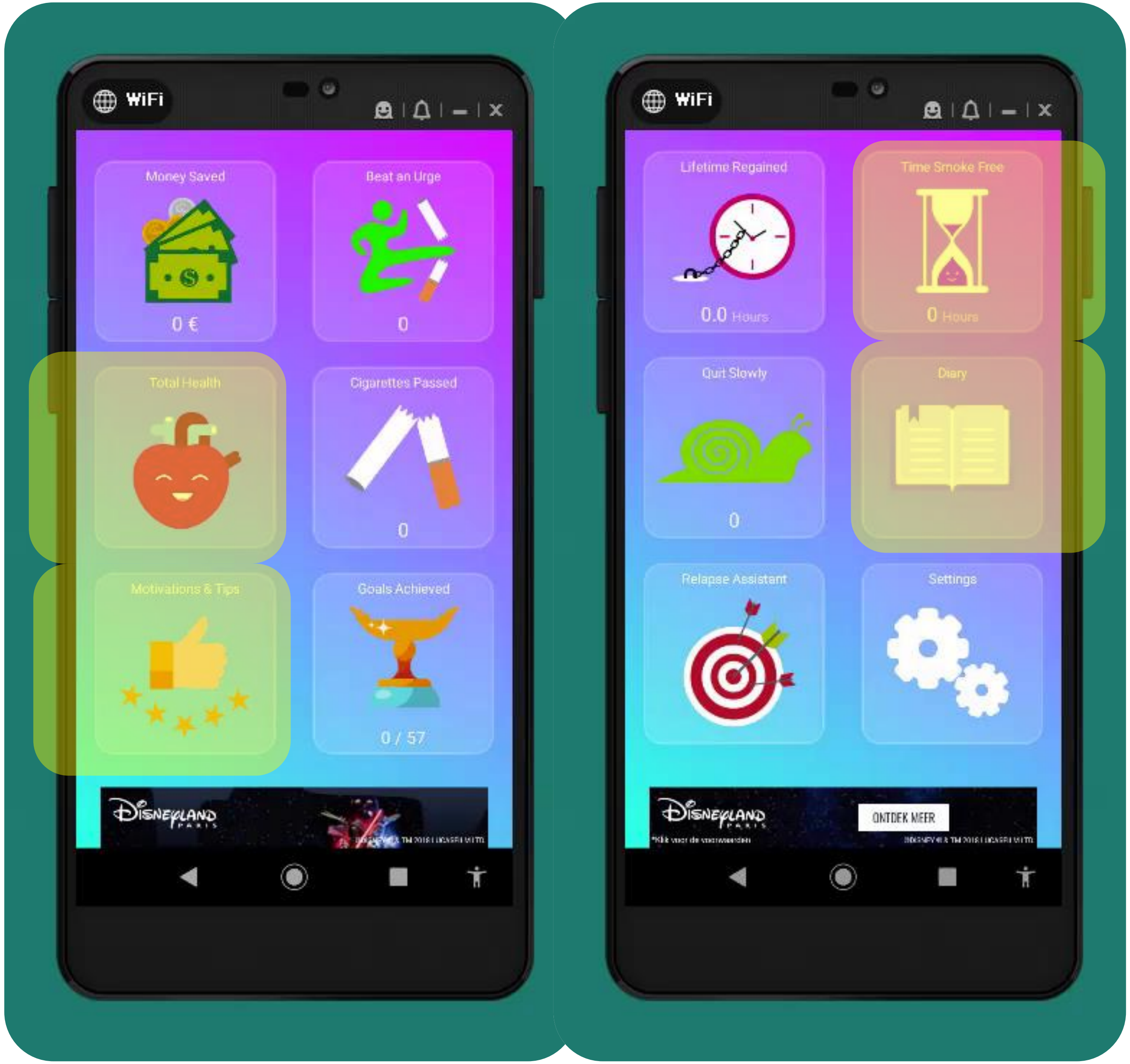

	Reageert
1	I: Okay. I think that does paint a picture, I think. I'm actually curious, if you have a first impression like that now, is it what you expected, based on the information you had beforehand, is the app as you expected, or are there things different? [#01:15:21-4#]<br><br>R: No, yes, it's clear. That's what I like about it, that you can actually see it in one scroll. See what it does. You don't have to fill it all in, keep a diary, or an ehh.... And here it's just an hourglass, so you can see how many hours, but it's not really prominent when you open it or something. Maybe it will do that later, when you have it fully booted up. [#01:15:53-9#]<br><br>I: Yeah. Okay. [#01:15:56-4#]<br><br>R: 'Tis well, um, yeah, not too many icons. Just six, twelve I mean. And also so your "Total Health. Yeah, I think that's kind of easy. It seems like, um.... [#01:16:16-1#]<br><br>I: What do you think you find easy about it, or nice? [#01:16:21-0#]<br><br>R: Yeah, that it's sort of organized, and that it's not too many things. And that you're "noncommittal... That you're not on everything... Not that there's all kinds of other things that you then... So, that it's automatically all motivational things, and... It seems to me, that if I say "Motivation & tips" here, then you get a...

Doet	Reden
• Klikt op button ´Motivation & Tips’	–

App #1 – EasyQuit free

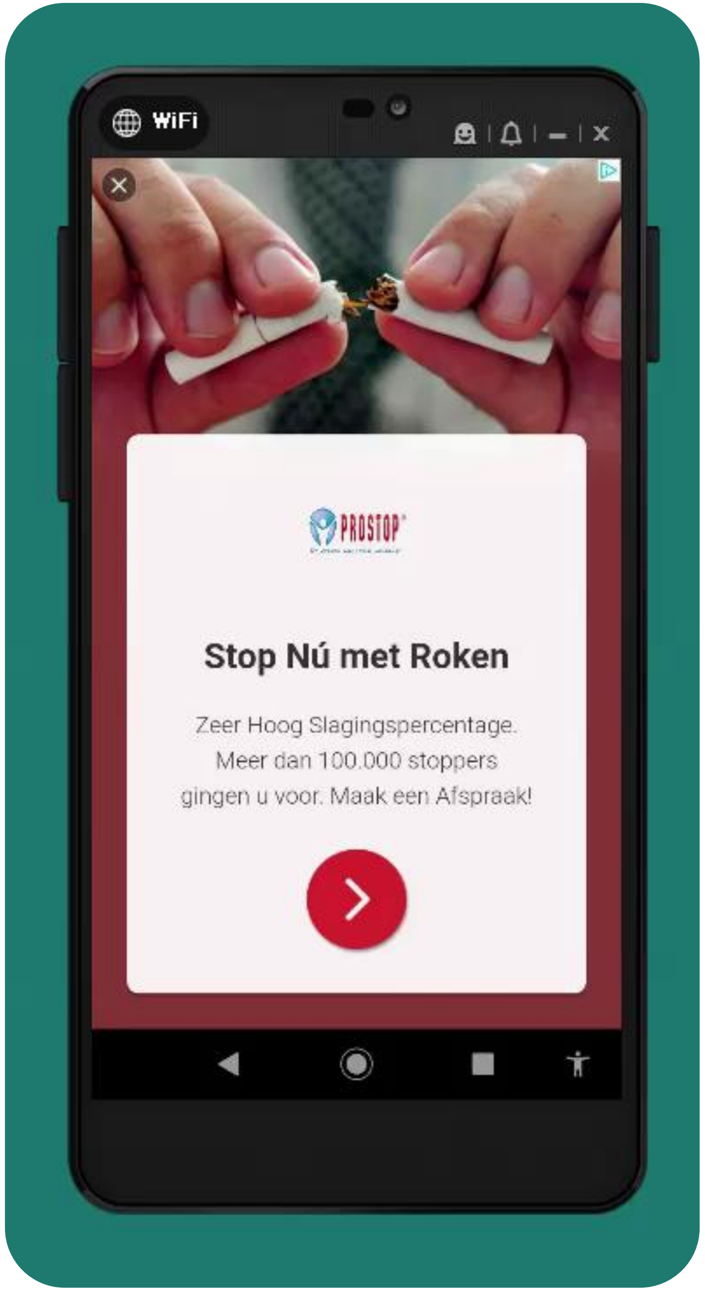

	Ziet/leest	Reageert	Information Cue
1		Um, yeah. Yes, here you get then now.... Oh here you get, wait, that's advertising of course [laughs].	

Functionaliteit App	Opmerking onderzoeker
• …	There is a bit of confusion here about what appears on the screen. It takes a while (11 seconds) for the participant to realize this is advertising and click away the add.

Doet	Reden
• Klikt op kruisje linksboven	–

App #1 – EasyQuit free

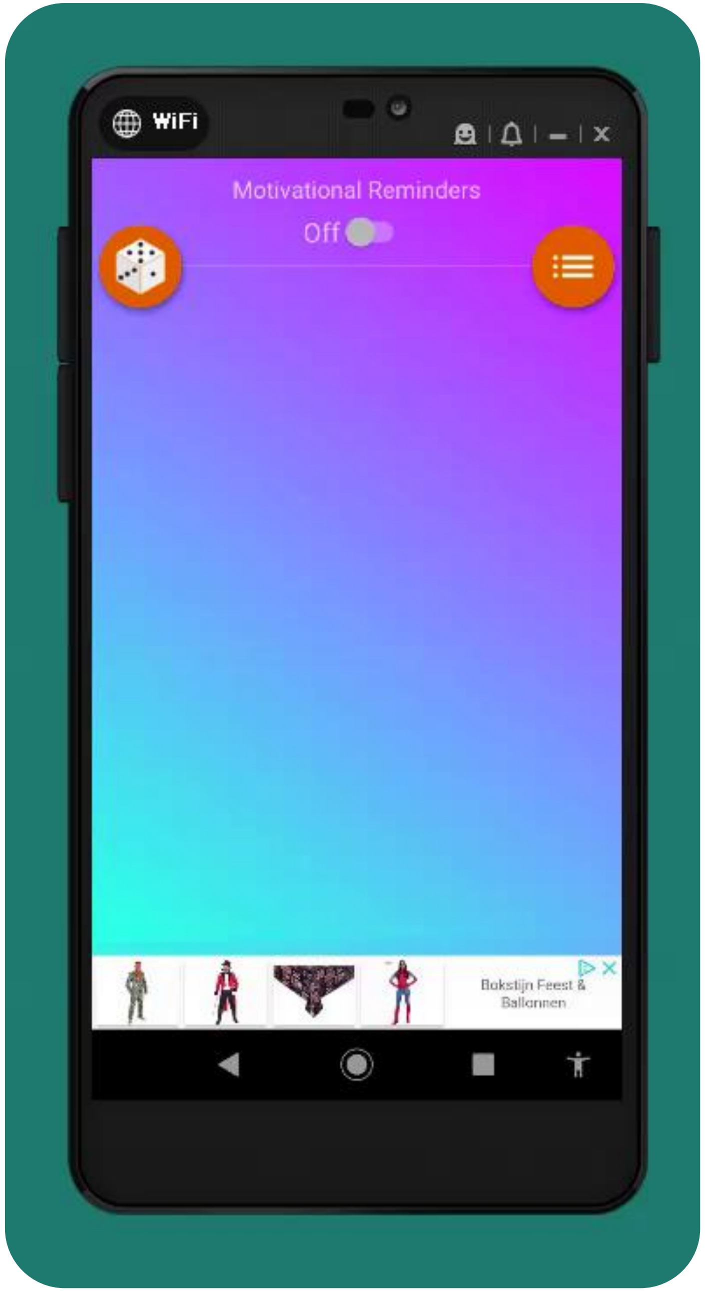

	Ziet/leest	Reageert	Information Cue
1		Here, look, it also says you can turn it on and off, the "motivational...". [#01:17:04-9#]	

Functionaliteit App	Opmerking onderzoeker
• ...	

Doet	Reden
• ...	–

App #1 – EasyQuit free

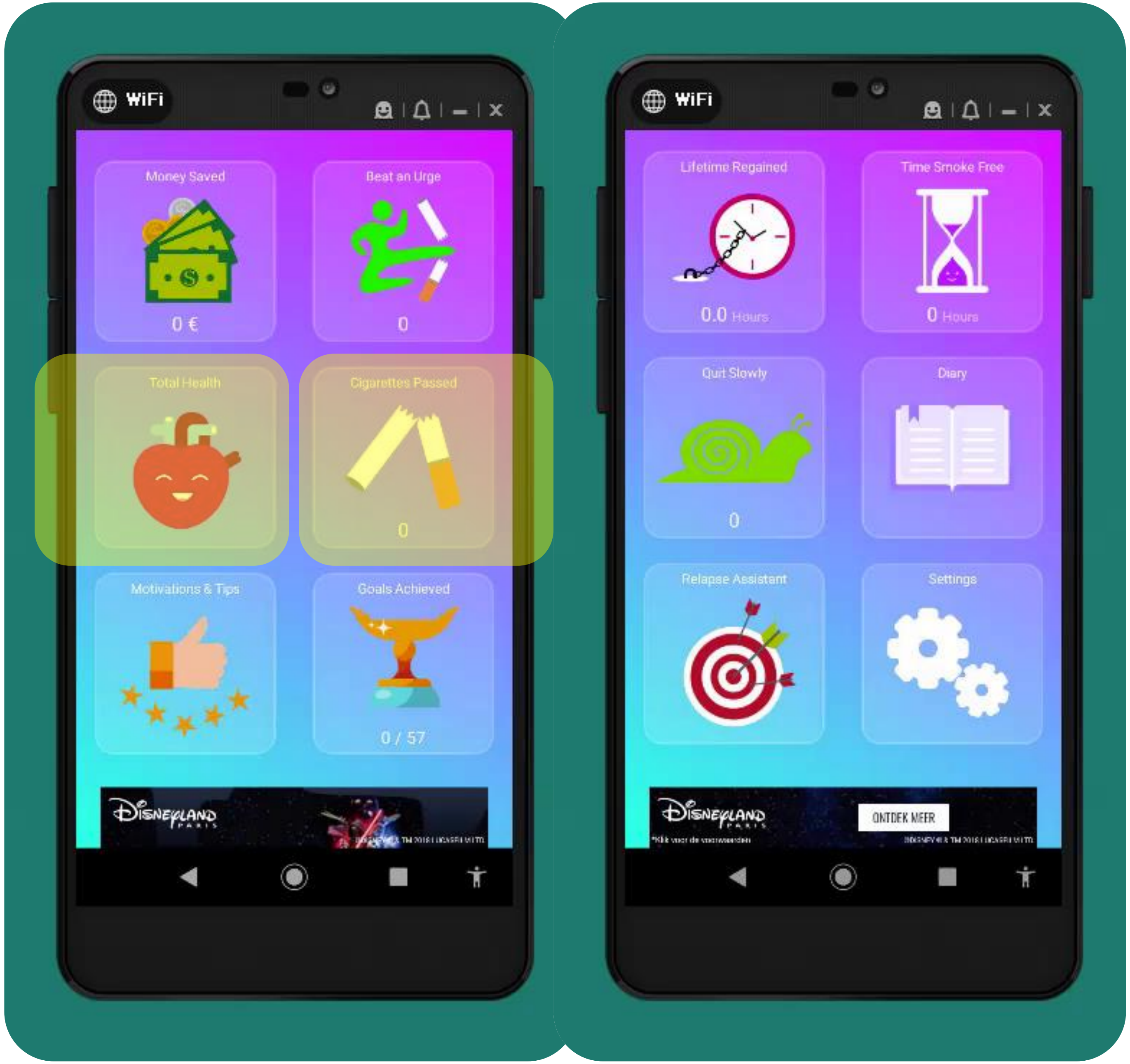

	Reageert
1	I: Is it kind of true that you like this, a palette of choices that you can put on and off, that you have control over. Is that what you mean to say? [#01:17:15-0#]<br><br>R: Yes, actually I do, I think, yes. That I do have control myself... [#01:17:21-9#]<br><br>I: And how do you think this app is going to help you quit smoking? [#01:17:28-2#]<br><br>R: Well, then if I maybe feel like having a cigarette, I open that thing, and then it does say how many cigarettes you've already so... Here it says 'cigarettes passed', it obviously counts up how many cigarettes you've already smoked less. Maybe you can get something out of it like, oh yeah, that's okay, I'll hang in there. And then maybe also 'Total Health', if you ehh....

Doet	Reden
• Klikt op button ‘Total Health’	–

App #1 – EasyQuit free

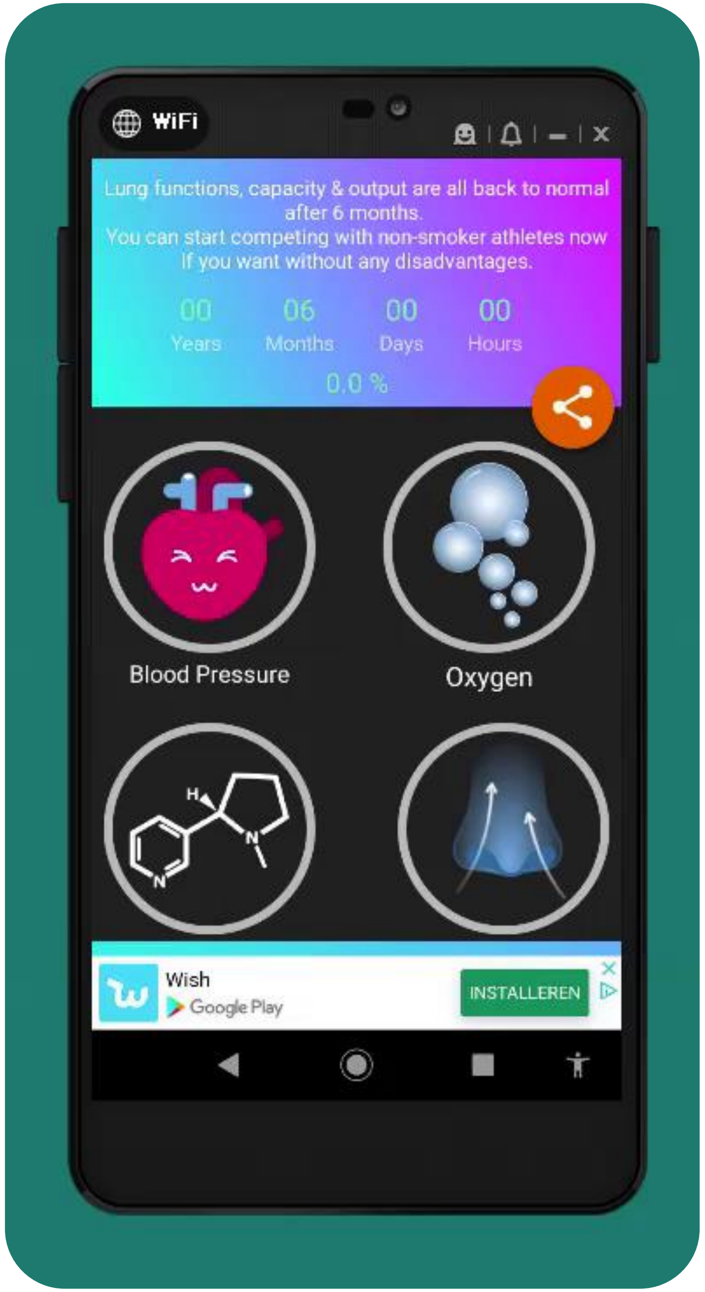

	Ziet/leest	Reageert	Information Cue
1		That there is also something on it, of, ehh, that it is good for your health of course. So that's nice, that you have all these funny icons here. They will all get better, of course, the longer you stop. [#01:18:25-3#]<br><br>I: Yeah, so the counter, and the health monitoring actually, those are things that are going to help you. From that list of features, are there any other things that you think, that would help? I don't know actually... Doesn't have to, but... [#01:18:45-1#]<br><br>R: Well... [#01:18:50-0#]<br><br>I: That game? [#01:18:50-9#]<br><br>R: Yes, that game maybe too. That's this one, I think.	

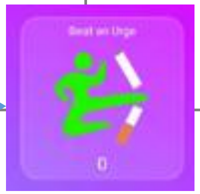

Functionaliteit App	Opmerking onderzoeker
• …	

Doet	Reden
• Gaat terug naar Homepage<br>• Klikt op button 'Beat an urge'	–

App #1 – EasyQuit free

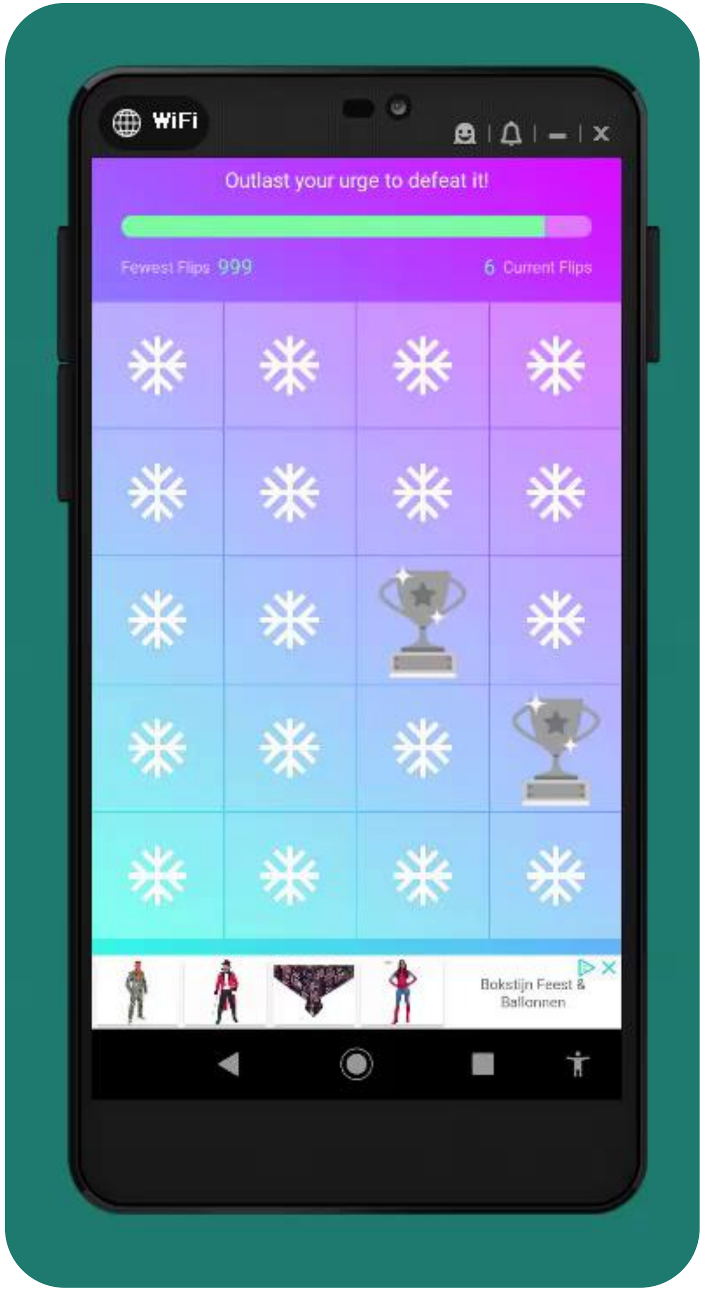

	Reageert
1	R: Oh yeah. That might still be funny then. [Laughs] Yeah, so you've been at that for three minutes.... Then of course that's funny. [#01:19:12-5#]<br><br>I: Yeah, the idea is funny, you mean. [#01:19:16-0#]<br><br>R: Yes, the idea is kind of funny. [#01:19:18-4#]<br><br>I: You've done it for a quick few seconds now. Do you still think it would help you now? [#01:19:22-3#]<br><br>R: Yeah, I don't know. Maybe though, that maybe then I might.... Yes, maybe when I'm lying on the couch and I think, oh, I'll have a cigarette. Maybe at the beginning... Yes, I don't know exactly. Maybe later on, you think... Not if I then... Yes, probably, if you stop, then the first time it's really difficult, then of course when you go to a bar, or you're somewhere with friends having a beer, then someone lights a cigarette, then of course those are more difficult... And then I don't say 'I'm going to play some memory games'. I don't think I'm going to do that. [laughs] [#01:20:12-6#]<br><br>I: So you think: in certain situations it would help, that memory, at home, and in the pub it wouldn't. [#01:20:19-4#]<br><br>R: Yeah, I think so, yeah. Would be funny, but no....<br><br>I: Okay. Yeah, I think we should start rounding up a little bit too, because we've been at it quite a long time. I was actually curious: is this your final choice or are you saying 'I didn't get to make a choice, I didn't choose an app. [#01:20:39-4#]<br><br>R: Oh no, I do want to fiddle with this for a while, I think. I think this is kind of funny actually. [#01:20:48-4#]

Functionaliteit App	Opmerking onderzoeker
• …	

Doet	Reden
• Klikt op een aantal velden	–

Factsheet participant #4

Naam app	Ontwikkelaar	Rating	# ratings/reviews	Ranking	# downloads	Gekozen/bekeken	Volgorde bekeken	Deelnemer	Store
Stop Smoking - EasyQuit free	Mario Hanna	4,8	54K	4	1mln.+	**gekozen**	1	4	Google Play Store
Stopstone	Trimbos Instituut	3,0	10	13	5K+	bekeken	2	4	Google Play Store
Quit Smoking Hypnosis Program	Mindifi LLC	4,1	335	29	10K+	bekeken	3	4	Google Play Store
Stop Smoking in 2 Hours	Juicemaster	4,2	1K	11	100K+	bekeken	4	4	Google Play Store
Flamy	Hartmut Offenwanger*	4,7	212	8	10K+	bekeken	5	4	Google Play Store

#	4
m/v	m
Leeftijd	36
Opleiding	MBO
Rookt sinds	vanaf 18de
Rookt hoeveel	4 á 20 / dag **
Gestopt op t2?	ja
App gebruikt?	ja
Eerder smr-app gebruikt	nee
Andere health apps?	ja, Strava
Belangrijk	7
Gereed	8
Zeker	7
Vertrouwen 'een app'	-
Belang 'goede keuze'	-
Vertrouwen 'deze app'	6 á 7
Intentie app te gebruiken	8

Aantal bekeken	5
Aantal gedownload	1
Gekozen app	#1
Gezocht op...	Sony xperia
Store	Google Play Store

Opmerking onderzoeker

* The respondent was shown a different name (Offline First) as the developer during the search. But this app was created by Hartmut Offenwanger.
** Participant usually smokes about 4 cigarettes a day, and about 20 on 'festive occasions'.

Bekeken / gebruikte information cues			
Zoekresultatenoverzicht		App-detailpagina	
Logo	✓	Logo	
Naam app	✓	Naam app	
Rating (*)	✓	Naam ontwikkelaar	
Naam ontwikkelaar	✓	Categorie	
Prijs	✓	Prijs ('Installeren' als gratis)	
		Info in-app aankopen	
		Rating (*)	✓
		Rating (#)	✓
		Downloads (#)	✓
		PEGI	
		Screenshots	
		Beschrijving	✓
		Rating (verdeling)	
		Reviews	✓
		Contactgegevens ontwikkelaar	
		Aanbevelingen	

Things I noticed about this respondent

- He 'seizes' whatever comes his way; makes use of opportunities to stop smoking (R&N, after 'too much', this survey)
- He does not want to 'have to' do anything, he wants to do things himself, own responsibility
- The scores on the 'motivation scale' to stop smoking do not seem to correspond completely with what he tells. He makes a rather phlegmatic impression towards smoking: 'doesn't necessarily have to stop', 'doesn't smoke that much', 'sometimes just stops when it has been too much for a while'.
- He seems driven by curiosity (and things he finds funny and interesting; things that excite him) and chooses to view apps with: - funny icon - hypnosis - quit in 2 hours - because the text of the subtitle is not quite legible in the results overview - developed by the Trimbos Institute Also in a previous quit attempt, curiosity ("see if anyone notices") drove him to stay quit
- He expects (wants) to get inspiration about 'what an app can do' or 'how an app can help me' during the search
- Elements he named aloud while searching and looking at the app: - game (memory) - information based on scientific insights - counters: money, time and number of cigarettes - choice to use or not certain components - badges (+/-) - tips for quitting - situation analysis - progress information on health - interface (appearance of the app, clarity, user-friendliness (?) - diary - motivational messages)push messages) (-) - social network as a helpline (involve friends and family, bets) (-) - possibility to quit slowly
- It seems like every time he sees something new, he wonders if it is something for him or not; if he sees himself using it; what he thinks of it.
- He is very "text oriented" and reads mostly the descriptions and reviews. In the former he seems to be always looking for 'effective elements' and in the latter he is looking for confirmation of the presence of those elements.
- Here and there he fills in things about an app, based on fairly minimal information. For example: on the basis of the icon with the drawn female and the title (Quit Buddy) he immediately imagines all sorts of things about how the app works. He does not look at the details page for that app. Another example: an app has hundreds of ratings but only one review and on that basis the respondent seems to think that the information is not reliable.
- Only when asked, he tells something about the importance (to him) of aspects such as: -price of an app -PG13 -number of downloads -screenshots and movies -privacy. These things (therefore) seem less important to him than the texts (of description and reviews).
- He indicates that he is suspicious of ratings and reviews, and info about the app (in descriptions but also, for example, in promo films).
