# Supplementary material for: Process and Information Needs When Searching for and Selecting Apps for Smoking Cessation: Qualitative Study Using Contextual Inquiry
Source: JMIR Hum Factors. 2022 Apr 14;9(2):e32628. doi: 10.2196/32628 (PMC9052019; doi:10.2196/32628)
Supplement: Multimedia Appendix 4 [file humanfactors_v9i2e32628_app4.pdf]

Standards for Reporting Qualitative Research (SRQR)

[This is a Multimedia Appendix to a full manuscript published in the J Med Internet Res. For full copyright and citation information see http://dx.doi.org/10.2196/jmir.32628]

[This report is based on Table 2 in Supplemental Digital Appendix 1 (found at http://links.lww.com/ACADMED/A218) to O'Brien et al. (2014) [50]]

No.	Topic	Item	Reference to page number or additional notes regarding current article
**Title and abstract**			
S1	Title	Concise description of the nature and topic of the study Identifying the study as qualitative or indicating the approach (e.g., ethnography, grounded theory) or data collection methods (e.g., interview, focus group) is recommended.	p.1 – The title of our article contains both the topic of the study (the process and information needs when searching for and selecting apps for smoking cessation) as well as an indication of the approach (qualitative study). We chose to use this broad term instead of 'contextual inquiry' in the title because the latter may not be a known method in this field.
S2	Abstract	Summary of key elements of the study using the abstract format of the intended publication; typically includes background, purpose, methods, results, and conclusions.	p.1 – In accordance with guidelines provided by O'Brien et al. (2014) we feel that the abstract of our article can be read "independent of the manuscript [to] get a sense of the background, purpose, methods, main results and implications that will be described in greater detail in the manuscript. The information presented in the abstract should be consistent with the information presented in the full text."
**Introduction**			
S3	Problem formulation	Description and significance of the problem/phenomenon studied; review of relevant theory and empirical work; problem statement.	p.1-3 – The "theoretical and/or practical issues or concerns that make the study necessary" are described in the Introduction under the headings: Background, Challenges in searching and selecting health apps and Related research.
S4	Purpose or research question	Purpose of the study and specific objectives or questions.	p.3,4 – The "statement of study intent" can be found in the Introduction under the heading Objectives
**Methods**			
S5	Qualitative approach and research paradigm	Qualitative approach (e.g., ethnography, grounded theory, case study, phenomenology, narrative research) and guiding theory if appropriate; identifying the research paradigm (e.g., postpositivist, constructivist/ interpretivist) is also recommended; discuss rationale[1].	* p.4 – Explanation why the chosen approach (contextual inquiry) is appropriate for the research question<br>* The starting point of this study is to identify the different ways people search for a smoking cessation app. We assumed in advance that there would be diversity in beliefs, interpretation of information,

[1] The rationale should briefly discuss the justification for choosing that theory, approach, method, or technique rather than other options available, the assumptions and limitations implicit in those choices, and how those choices influence study conclusions and transferability. As appropriate, the rationale for several items might be discussed together.

			and personal information needs. This implies a belief that there is no one 'truth'. We focus on 'understanding', use inductive reasoning, and believe that meaning is constructed in the researcher-participant interaction in the natural environment. All of this aligns with an Interpretivist paradigm (Bunniss & Kelly, 2010 [90]).
S6	Researcher characteristics and reflexivity	Researchers' characteristics that may influence the research, including personal attributes, qualifications/experience, relationship with participants, assumptions, and/or presuppositions; potential or actual interaction between researchers' characteristics and the research questions, approach, methods, results, and/or transferability.	The research was primarily designed, developed, and conducted by YH. YH is a PhD student, who has experience with both quantitative and qualitative research through doing two master's studies. Through some journalistic work experience, the researcher was used to and familiar with interviewing. Contextual Inquiry, however, she had not done before. In the context of 'Researcher characteristics and reflexivity', two things are good to mention. First of all, despite the intrpretivist character of the studie, YH tends rather toward the post-positivist paradigm. This means that she has tried to pursue objectivity by recognizing the possible effects of her own theories, background, knowledge and values. During the interviews she thus took care not to bias the work with her own opinions. Secondly, it should be noted that due to YH's personality, all interviews were conducted in a very relaxed, laid-back manner, often with an almost personal connection between the researcher and the participants. This, regardless of the goal for objectivity, probably did influence the results.
S7	Context	Setting/site and salient contextual factors; discuss rationale.	The setting/site(s) in which the study was conducted is described on p.5 under Procedure & data collection. We reflect on pros and cons of this choice on p. 25, 26 under Strengths & limitations.
S8	Sampling strategy	How and why research participants, documents, or events were selected; criteria for deciding when no further sampling was necessary (e.g., sampling saturation); discuss rationale.	On p.4 under Sampling of participants, we describe how and why research participants were selected for the study. We reflect on pros and cons of this choice on p. 25, 26 under Strengths & limitations.
S9	Ethical issues pertaining to human subjects	Documentation of approval by an appropriate ethics review board and participant consent, or explanation for lack thereof; other confidentiality and data security issues.	p.7: Ethical approval for the study was obtained from the institutional review board of the first author's university: the Ethics Review Board of the Tilburg School of Social and Behavioral Sciences (reference EC-2018.92).
S10	Data collection methods	Types of data collected; details of data collection procedures including (as appropriate) start and stop dates of data collection and analysis, iterative process,	Data was collected from December 2018 until August 2019. Description of data collection methods can be found on p.5,6 under Procedure & data collection.

		triangulation of sources/methods, and modification of procedures in response to evolving study findings; discuss rationale.	
S11	Data collection instruments and technologies	Description of instruments (e.g., interview guides, questionnaires) and devices (e.g., audio recorders) used for data collection; if/how the instrument(s) changed over the course of the study.	Description of data collection instruments and technologies can be found on p.5,6 under Procedure & data collection.
S12	Units of study	Number and relevant characteristics of participants, documents, or events included in the study; level of participation (could be reported in results)	See p. 8 'Sample descriptive', as well as the paragraphs 'Quitting smoking' and 'Experience with smoking cessation aids and apps' on p. 6-9.
S13	Data processing	Methods for processing data prior to and during analysis, including transcription, data entry, data management and security, verification of data integrity, data coding, and anonymization/deidentification of excerpts.	The description of methods for processing data, including the instruments and technology that were used, can be found on p. 6,7 under Data analysis.
S14	Data analysis	Process by which inferences, themes, etc., were identified and developed, including the researchers involved in data analysis; usually references a specific paradigm or approach; discuss rationale.	See table 1 on p. 7
S15	Techniques to enhance trustworthiness	Techniques to enhance trustworthiness and credibility of data analysis (e.g., member checking, audit trail, triangulation); discuss rationale.	Independent co-coding of first transcript (p. 7), feedback of peers was sought in diverse research meetings (such as lab groups meetings), multiple iterations of the report were shared, discussed, and refined by all authors (p. 7), member checking by jointly creating a summary of the whole search process (p.6). We reflect on trustworthiness and validity in Strengths & limitations p. 25, 26.
**Results/findings**			
S16	Synthesis and interpretation	Main findings (e.g., interpretations, inferences, and themes); might include development of a theory or model, or integration with prior research or theory.	The main findings are described in the Results section (p. 7-22). We have chosen to separate (as much as possible) literal findings (what we observed) and interpretation of these events. Interpretation of, and integration with prior literature and theory, was done mainly in the Discussion (p. 23-28).<br>We have identified several facets that are involved in searching for, and selecting an app for smoking cessation. These (sub)themes are reported in table 2 (p.7) and under Principal Findings (p. 23-25). The Results section is however structured based on the various process steps, and not (as is usually done) on themes and subthemes. The

			clarification for this choice, can be found in the first paragraph of the Results section (p. 7).
S17	Links to empirical data	Evidence (e.g., quotes, field notes, text excerpts, photographs) to substantiate analytic findings.	We have provided a plethora of quotes. Furthermore, we have provided one process flow chart in the Multimedia Appendices as an illustration of both the method of analysis, as well as an example of the data we used.
**Discussion**			
S18	Integration with prior work, implications, transferability, and contribution(s) to the field	Short summary of main findings; explanation of how findings and conclusions connect to, support, elaborate on, or challenge conclusions of earlier scholarship; discussion of scope of application/generalizability; identification of unique contribution(s) to scholarship in a discipline or field.	A short summary of the main findings can be found directly under Principal findings. The description of the results is interwoven with some interpretation of the data in the context of previous findings (p. 23-25).
S19	Limitations	Trustworthiness and limitations of findings	See Strengths & limitations, p. 25, 26.
**Other**			
S20	Conflicts of interest	Potential sources of influence or perceived influence on study conduct and conclusions; how these were managed.	p.28
S21	Funding	Sources of funding and other support; role of funders in data collection, interpretation, and reporting.	This study was funded by the University of Tilburg as part of the principal researchers PhD project.

References

[51] O'Brien BC, Harris IB, Beckman TJ, Reed DA, Cook DA. Standards for reporting qualitative research: a synthesis of recommendations. Acad Med 2014 Sep;89(9):1245-1251 [doi: 10.1097/ACM.0000000000000388] [Medline: 24979285]

[52] Bunniss S, Kelly D. Research paradigms in medical education research. Med Educ 2010 Apr;44(4):358-366. [doi: 10.1111/j.1365-2923.2009.03611.x] [Medline: 20444071]
