# Supplementary material for: Process and Information Needs When Searching for and Selecting Apps for Smoking Cessation: Qualitative Study Using Contextual Inquiry
Source: JMIR Hum Factors. 2022 Apr 14;9(2):e32628. doi: 10.2196/32628 (PMC9052019; doi:10.2196/32628)
Supplement: Multimedia Appendix 5 [file humanfactors_v9i2e32628_app5.pdf]

Search queries

[This is a Multimedia Appendix to a full manuscript published in the J Med Internet Res. For full copyright and citation information see http://dx.doi.org/10.2196/jmir.32628]

Used search queries	Language	Translation
Stoppen met roken	Dutch	Quit smoking
Stoppen met roken apps	Dutch	Quit smoking apps
Quit smoking	English	Stoppen met roken
Quit smoking apps	English	Stoppen met roken apps
Smoking	English	Roken
Beste stoppen met roken apps	Dutch	Best quit smoking apps

Various search queries that were used by the participants.
